# Platform Synthetic Lectins for Divalent Carbohydrate Recognition in Water

**DOI:** 10.1002/anie.201603082

**Published:** 2016-06-17

**Authors:** Tom S. Carter, Tiddo J. Mooibroek, Patrick F. N. Stewart, Matthew P. Crump, M. Carmen Galan, Anthony P. Davis

**Affiliations:** ^1^ School of ChemistryUniversity of BristolCantock's CloseBristolBS8 1TSUK

**Keywords:** carbohydrates, dendrimers, molecular recognition, receptors, supramolecular chemistry

## Abstract

Biomimetic carbohydrate receptors (“synthetic lectins”) have potential as agents for biological research and medicine. However, although effective strategies are available for “all‐equatorial” carbohydrates (glucose, etc.), the recognition of other types of saccharide under natural (aqueous) conditions is less well developed. Herein we report a new approach based on a pyrene platform with polar arches extending from aryl substituents. The receptors are compatible with axially substituted carbohydrates, and also feature two identical binding sites, thus mimicking the multivalency observed for natural lectins. A variant with negative charges forms 1:2 host/guest complexes with aminosugars, with *K*_1_>3000 m ^−1^ for axially substituted mannosamine, whereas a positively charged version binds the important α‐sialyl unit with *K*_1_≈1300 m ^−1^.

Carbohydrate recognition is a central biological phenomenon that mediates a range of cellular processes.^1^ Carbohydrate‐binding molecules are important as research tools for investigating these processes, and potentially as diagnostic and therapeutic agents in medicine.^1^, ^2^, ^3^ Studies in this area most commonly use lectins, the major class of saccharide‐binding proteins, but lectins often lack the desired selectivities and tend to show low affinities (generally 10^3^–10^4^ m ^−1^ for monosaccharides).^4^ Moreover, as proteins, their therapeutic potential is limited by issues such as immunogenicity.^3^ There is consequently much interest in small‐molecule receptors, which could complement lectins and perhaps be developed for new types of application.^1^, ^3^, ^5^ However, the design of such molecules has proved difficult, especially for biomimetic systems based on noncovalent bonding.^6^ Although a variety of structures have been shown to be active in organic solvents,^7^ there are few which can operate in the natural but challenging environment of water.^8^

We have approached this problem by constructing symmetrical cavities with an aromatic “roof” and “floor” separated by polar spacers (e.g. **1**, Figure 1 a).^9^ The designs are complementary to saccharides with all‐equatorial substitution patterns (e.g. **2**), and have yielded encouraging results. Selectivities are good, and some affinities are above 10^4^ m ^−1^, even for uncharged substrates.9a,9b However, the selectivity for all‐equatorial carbohydrates is a constraint on potential applications, as many substrates of interest do not belong to this family. Herein we report an alternative design strategy which rationally targets carbohydrates with axial substituents and which, for the first time, mimics the multivalency exhibited by many lectins.^1^

**Figure 1:**
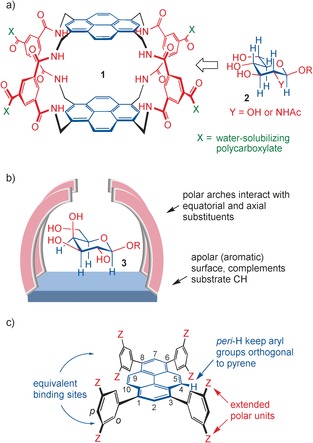
a) A receptor **1** for all‐equatorial carbohydrates **2** (see Ref. [9a]). The symmetrical cavity matches the polar (red) and apolar (blue) groups in the substrate. b) A strategy for binding saccharides with axial substituents, illustrated for the β‐galactosyl group **3** as the substrate. c) General design of the pyrene‐based receptors described herein.

While all‐equatorial saccharides possess two roughly similar hydrophobic patches, other carbohydrates tend to be facially amphiphilic. The inversion of a stereocenter, as in β‐galactosyl **3**, adds to the polarity of one face while decreasing the polarity of the other. A complementary binding site should therefore contain just one extended apolar surface, the remainder being mainly polar.^10^ An approach to such structures might involve an aromatic platform with polar substituents that can arch over a bound carbohydrate (e.g. Figure 1 b). When considering options for realizing this architecture, we noted the potential of 1,3,6,8‐tetraarylpyrenes (Figure 1 c). These compounds are readily prepared from tetrabromopyrene (**8**) by Suzuki–Miyaura methodology, and are forced to adopt nonplanar conformations owing to interactions between aryl and *peri*‐H groups.^11^ The *meta* positions on the aryl groups could provide anchor points for the polar “arches”. Stereoisomers would be possible in the general case, but could be avoided by using symmetrically substituted aryl groups, as shown in Figure 1 c. Interestingly, this arrangement would generate two equivalent binding sites, thus mimicking the multivalency common in lectins.^1^ Groups Z could be used to confer water‐solubility as well as to provide polar interactions. For example, polyionic dendrimers, which are highly water solubilizing and capable of hydrogen bonding to polar carbohydrate substituents, could be readily installed.9c

As a prototype for this design, we chose the tetracosacarboxylate **9**. Modeling^12^ showed that the dendrimers in **9**, though relatively small, possessed sufficient reach to interact with axial groups on a substrate. Protonated mannosamine **10**⋅H^+^, with an axial NH_**3**_^+^ group, was found to be an especially promising substrate (Figure 2). Receptor **9** was prepared in 23 % yield over four steps from diacid **4**, amine **5**,^13^ and 1,3,6,8‐tetrabromopyrene (**8**;^14^ Scheme 1).^12^ The anionic receptor **9** dissolved freely in water to give well‐resolved ^1^H NMR spectra, which were concentration‐independent below 1.2 mm, thus implying a monomeric species. Fluorescence spectra showed an emission maximum at 425 nm (excitation wavelength: 380 nm), detectable down to nanomolar concentrations and with intensity directly proportional to concentration.

**Figure 2:**
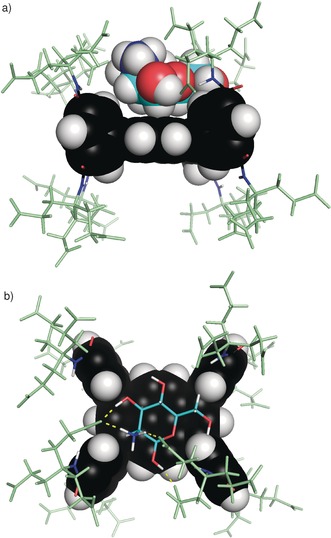
Model of receptor **9** bound to the β anomer of protonated mannosamine **10**⋅H^+^. Aromatic portions of the receptor are shown in space‐filling mode, side chains in pale green, mannosamine carbon atoms cyan. a) View from the side, with mannosamine in space‐filling mode. b) View from above, with mannosamine in stick mode. Hydrogen bonds are shown in yellow.

**Scheme 1:**
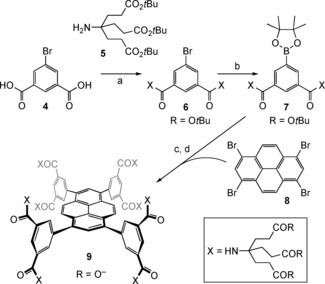
Synthesis of receptor **9**. a) SOCl_2_, reflux; then **5**, EtN*i*Pr_2_, THF, 53 %; b) [Pd(dppf)Cl_2_], bis(pinacolato)diboron, KOAc, dioxane, 80 °C, 80 %; c) **8**, [Pd(dppf)Cl_2_], Cs_2_CO_3_, dioxane, H_2_O, 90 °C, 53 %; d) CF_3_CO_2_H, SiHEt_3_, CH_2_Cl_2_; then aqueous NaOH, quantitative. dppf=1,1′‐bis(diphenylphosphino)ferrocene.

Carbohydrate recognition was studied by fluorescence and ^1^H NMR titrations of **9** with a range of sugars.^12^ Solutions were adjusted to pH 7, and the pH value was confirmed to be unchanged after each experiment. Titrations with aminosugars **10**–**12** (protonated at pH 7) 
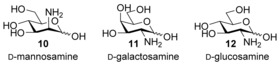
yielded clear evidence of complex formation. For example, in NMR titrations, the addition of mannosamine (**10**) was accompanied by downfield changes in the positions of the receptor aromatic signals (see Figure 3 a). A Job plot^15^ based on the receptor 4‐H signal displacements gave a maximum at **9**/**10**=1:2, thus confirming divalency (see Figure S14 in the Supporting Information). Analysis of the receptor signals, assuming the 1:2 binding model, gave stepwise binding constants *K*_1_=3120 m ^−1^ and *K*_2_=540 m ^−1^ (see Figure S9).^16^ As *K*_1_=4 *K*_2_ for noncooperative two‐site binding,^17^ it seems the two associations are almost independent. The results were supported by fluorescence titrations of **9** with **10**, which showed large increases in receptor emission intensity and could be analysed to give almost identical binding constants (Figure 4 and Table 1).

**Figure 3:**
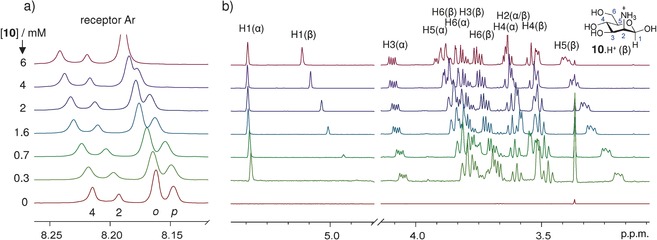
Partial ^1^H NMR titration spectra of receptor **9** (0.20 mm) with d‐mannosamine (**10**) in D_2_O at pH 7 and 298 K. a) Signals from receptor aromatic hydrogen atoms. For numbering, see Figure 1 c. b) Mannosamine signals, with intensities normalized to compensate for the increase in concentration. The mannosamine H1(β) signal is affected by the water‐suppression sequence and is artificially depressed in the early stages of the titration.

**Figure 4:**
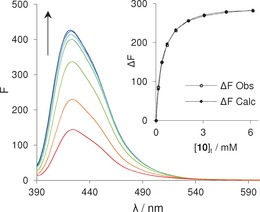
Fluorescence emission spectra and binding curve of receptor **9** (0.33 μm) titrated with d‐mannosamine (**10**) in H_2_O at pH 7 and 298 K. Excitation wavelength: 380 nm.

**Table 1:** Cumulative association constants (*K*_1_, *K*_2_) for 1:1 and 1:2 binding of receptor **9** with aminosugars in aqueous solution, as determined by ^1^H NMR and fluorescence titrations.^[a]^

Carbohydrate	*K*_1_ [m ^−1^]	*K*_2_ [m ^−1^]
d‐mannosamine (**10**)	3120,^[b]^ 3120^[c]^	540,^[b]^ 600^[c]^
d‐galactosamine (**11**)	1800,^[b]^ 2000^[c]^	200,^[b]^ 180^[c]^
d‐glucosamine (**12**)	1040,^[b]^ 1100^[c]^	410,^[b]^ 360^[c]^

[a] pH 7, *T*=298 K. [b] ^1^H NMR titration. [c] Fluorescence titration.

Changes in the positions of carbohydrate signals during the ^1^H NMR titration were also informative (Figure 3 b). All the signals from **10** moved downfield during the titration implying that, as expected, these protons are shielded in the complex.^18^ The spectra provide separate information for α and β anomers, present in the ratio 1 : 1.9.^19^ Signals for hydrogen atoms on the α face of the β anomer [for example, H1(β), H5(β)] showed especially large movements, consistent with the modeled structure (Figure 2). From the α anomer, the proton H5(α) signal also moved substantially. NOE data (see Figure S15) confirmed that **9** and the carbohydrate are closely associated. Binding could not be quantified reliably because of the complexity of the system (two substrates, both forming 1:1 and 1:2 complexes). However, the signal movements were consistent with *K*_1_≈3000 m ^−1^ for both anomers.

Titrations with galactosamine (**11**) and glucosamine (**12**) gave similar changes in both ^1^H NMR and fluorescence spectra. Analysis of the data gave the binding constants shown in Table 1. Affinities were somewhat lower, suggesting that the binding sites of **9** favor axial NH_3_^+^ substitution. Uncharged monosaccharides did not appear to bind, but titrations with the disaccharides cellobiose, lactose, and maltose, and also methylamine, gave evidence of weak‐complex formation (*K*_1_≤16 m ^−1^).^12^ It thus seems that the high affinities for aminosugars result from combining carbohydrate‐specific interactions (hydrophobic, CH–π, hydrogen bonding) with electrostatic attraction.

Whereas cationic sugars are relatively uncommon in nature, anionic carbohydrates are widespread and play important roles. Especially significant is the α‐linked *N*‐acetylneuraminic acid (α‐sialyl) unit **13**. 
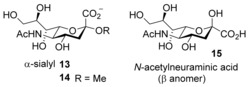
This moiety commonly appears as a terminus of oligosaccharides, accessible for binding and therefore an important potential target.^20^ The substitution pattern in **13** is all‐equatorial with an additional axial negatively charged substituent. This compound should be nicely complementary to our platform design (Figure 1 b), provided that the polar arches are positively charged.

To test this concept, we prepared the cationic receptor **16**, possessing 24 guanidinium units (see the Supporting Information). 
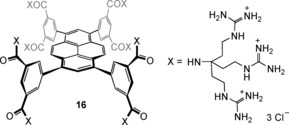
Guanidinium substituents were chosen to ensure that the receptor would be fully protonated at pH 7.^21^ Spectroscopic studies implied that receptor **16** is monomeric in water below a concentration of 1.2 mm. As expected, the ^1^H NMR spectrum of **16** was unaffected by the pH value in the range 6–8, confirming full protonation.

Studies of **16** as a receptor for the α‐sialyl unit **13** required a model substrate. The parent saccharide *N*‐acetylneuraminic acid is readily available, but exists mainly (>90 %) as the β anomer **15**, and is thus unrepresentative of **13**. Simple α‐sialosides are not commercially available, and we therefore synthesized the methyl derivative **14** through a variation of a literature procedure.^12^ The binding of **14** to **16** was studied by ^1^H NMR and fluorescence spectroscopy. NOESY cross‐peaks between substrate and receptor aromatic proton signals supported complex formation (see Figure S55). On the other hand, NMR titrations yielded relatively small changes in the signal positions, implying a looser geometry than for **9**+**10**. A Job plot based on a receptor aromatic signal confirmed the expected **16**/**14**=1:2 binding stoichiometry (see Figure S54). For quantitative analysis, we employed a titration in which receptor **16** was added to substrate **14**. The carbohydrate signals moved upfield as expected, and several could be followed throughout (see Figure S57). Simultaneous analysis of four of these signals was consistent with three successive binding events, with *K*_1_, *K*_2_, and *K*_3_=1310, 570, and 30 m ^−1^, respectively. Given the high density of positive charge on **16**, it is reasonable to suppose that a third (and possibly a fourth) molecule of **14** might bind to the receptor.

Fluorescence titration of **16** with **14** yielded a surprisingly strong effect (Figure 5); receptor emission was reduced almost to zero by addition of the carbohydrate. Analysis of the changes assuming a 1:2 binding model (see Figure 5) gave stepwise binding constants *K*_1_, *K*_2_=1300, 790 m ^−1^, consistent with the NMR data. The decrease in emission was found to depend on the chloride counterions; when these were replaced with trifluoroacetate, fluorescence increased on binding. We presume that the addition of **14** to **16** causes a rearrangement of the counterions, thus promoting fluorescence quenching.

**Figure 5:**
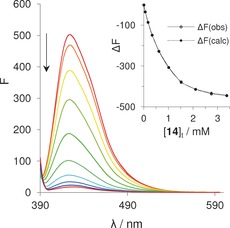
Fluorescence emission titration spectra and binding model fit of receptor **16** (0.50 μm) with methyl sialoside (**14**) in H_2_O at pH 7 and 298 K. Excitation wavelength: 380 nm.

Fluorescence titrations were also performed for receptor **16** with several uncharged monosaccharides.^12^ Efficient quenching was again observed, although analysis suggested that binding was much weaker than for **14**. Moderate affinities were estimated for methyl β‐d‐glucoside (*K*_1_=43 m ^−1^), methyl β‐d‐galactoside (*K*_1_=46 m ^−1^), and galactose (*K*_1_=17 m ^−1^). In these cases, NMR shifts were too small for analysis, but association between the receptor and the carbohydrate was confirmed by NOE enhancements.^22^ Sodium acetate was also bound weakly (*K*_1_=17 m ^−1^). The affinity for glucose was too small to be quantified implying that, as intended, the platform design can reverse the selectivity shown by our earlier synthetic lectins.

In conclusion, we have reported a rational design for synthetic lectins which mimic the multivalency shown by natural lectins and, unlike earlier systems, can accommodate substrates with axial substituents. There is scope for varying the system, for example, by altering/extending the side chains or changing one aryl substituent. The potential for binding the β‐sialyl group **13** is especially significant, and will be a focus of future efforts.

## Supporting information

As a service to our authors and readers, this journal provides supporting information supplied by the authors. Such materials are peer reviewed and may be re‐organized for online delivery, but are not copy‐edited or typeset. Technical support issues arising from supporting information (other than missing files) should be addressed to the authors.

Supplementary
