# Supplementary material for: Platform Synthetic Lectins for Divalent Carbohydrate Recognition in Water
Source: Angew Chem Int Ed Engl. 2016 Jun 17;55(32):9311–5. doi: 10.1002/anie.201603082 (PMC5006853; doi:10.1002/anie.201603082)
Supplement: Supplementary file 1 — Supplementary [file ANIE-55-9311-s001.pdf]

Angewandte Chemie
Eine Zeitschrift der Gesellschaft Deutscher Chemiker

Supporting Information

Platform Synthetic Lectins for Divalent Carbohydrate Recognition in Water

*Tom S. Carter, Tiddo J. Mooibroek, Patrick F. N. Stewart, Matthew P. Crump, M. Carmen Galan, and Anthony P. Davis**

anie_201603082_sm_miscellaneous_information.pdf

Electronic Supplementary Information

Contents

1. Synthetic Procedures 3

General Experimental 3

Anionic Receptor **9** 4

1,3,6,8-Tetrabromopyrene (**8**) 4

[Di-tert-butyl 3-[2-(tert-Butoxycarbonyl)ethyl]-4-nitroheptanedicarboxylate] (**A**) 4

[Tri-tert-butyl 3,3′,3″-(1-aminomethanetriyl)tripropanoate] (Behera's amine) (**5**) [2] 6

Aryl Bromide **6** 7

Aryl Boronate **7** 8

O-Protected Receptor **B** 9

Anionic Receptor **9** 10

Cationic Receptor **16** 11

3-(2-Aminoethyl)-3-nitropentane-1,5-diamine.3HCl (**C**) 11

Nitromethanetris(2-tert-butoxycarbonylaminoethane) (**D**) [3] 12

Aminomethanetris(2-tert-butoxycarbonylaminoethane) (**E**) [3] 13

N-Boc Protected Aryl Bromide **F** 14

N-Boc Protected Aryl Boronate **G** 15

N-Boc Protected Tetracosa-amine **H** 16

N-Boc Protected Receptor **I** 17

Cationic Receptor **16** 19

Methyl 5-acetamido-3,5-dideoxy-D-glycero-α-D-galacto-2-nonulosonic acid **14** 20

N-Acetyl neuraminic acid methyl ester **J** 20

Methyl 5-acetamido-4,7,8,9-tetra-O-acetyl-2-chloro-2,3,5-trideoxy-D-glycero-D-galacto-2-nonulopyranosonate (**K**) [4] 21

Methyl (methyl 5-acetamido-3,5-dideoxy-α-D-glycero-D-galacto-2-nonulopyranosid)onate (**L**) [4] 22

Methyl 5-acetamido -3,5-dideoxy-D-glycero-α-D-galacto-2-nonulosonic acid (**14**) 24

2. Spectroscopic and Binding Studies 25

General Methods for Binding Studies 25

Anionic Receptor **9** – Spectroscopic Studies. 27

Anionic Receptor **9** – Binding Studies. 30

Mannosamine **10** 30

Galactosamine **11** 38

Glucosamine **12** 43

Cellobiose 48

Lactose 52

Maltose 52

Cationic Receptor **16** – Spectroscopic Studies 55

Cationic Receptor **16** – Binding Studies. 58

Methyl α-sialoside **14** 58

Methyl β-D-glucoside 65

Methyl β-D-galactoside 68

Glucose 71

Mannose 72

Galactose 73

Molecular Modelling **78**

References **80**

1. Synthetic Procedures

General Experimental

Commercial reagents were purchased from Sigma-Aldrich, Alfa-Aesar or Acros Organics and were used without further purification unless otherwise specified. Carbohydrates employed in binding studies were purchased from Sigma-Aldrich or Carbosynth Ltd.

All air and moisture sensitive manipulations were carried out using standard vacuum line and Schlenk techniques, or in a drybox containing a purified argon atmosphere. Solvents for air and moisture sensitive manipulations were obtained from an Anhydrous Engineering Solvent Purification System or distilled and dried over activated molecular sieves.

Column chromatography was performed using silica gel 60 (Sigma Aldrich) and a suitable eluent. TLC was performed using aluminium backed TLC plates (Merck-Keiselgel 60 F_{254}) and visualised using UV fluorescence and/or developed using ninhydrin, potassium permanagante, $EtOH/H_2SO_4$, vanillin, $Pd(OAc)_2/H_2O$ or iodine.

HPLC chromatography was performed using a Waters 600 Controller with a Waters 2998 Photodiode Array Detector. For analytical runs a XSELECT CSH C18 5 µm (4.6x150 mm) column was used and for preparative runs a XSELECT CSH Prep C18 5 µm OBD (19x250mm) column was used, normally with an Acetone-Water solvent mixture.

^{1}H and ^{13}C NMR spectra were recorded on Varian VNMR 400 MHz, Jeol Eclipse 400 MHz, Varian VNMR 500 MHz or Varian VNMRS600 Cryo 600 MHz spectrometers. All spectra were obtained at ambient temperature unless stated otherwise. All ^{1}H and ^{13}C NMR chemical shifts are reported relative to tetramethylsilane as an internal standard and in $CDCl_3$ unless otherwise stated, with ^{1}H (residual) and ^{13}C chemical shifts of the solvent as a secondary standard. The ^{1}H NMR spectra are provided for all compounds as evidence of purity.

IR spectra were recorded on Perkin-Elmer Spectrum One FT-IR spectrometer with an ATR accessory and frequencies reported in wavenumbers (cm^{-1}). ESI-LRMS (electrospray ionisation low resolution mass spectrometry) was performed on a VG Analytical Quattro, ESI-HRMS (electrospray ionisation high resolution mass spectrometry) was performed on a Bruker Daltonics Apex IV and MALDI-MS (matrix-assisted laser desorption/ionisation) was performed on an Applied Biosystems 4700. Elemental analysis was performed on a EuroVector EA3000 Elemental Analyser.

Anionic Receptor 9

1,3,6,8-Tetrabromopyrene (8) [1]

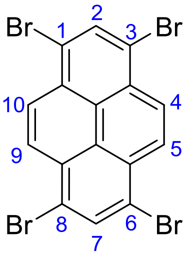

8

A two-necked 1 L roundbottom flask equipped with a condenser and a mechanical stirrer was charged with pyrene (10.1 g, 50.0 mmol) and 350 ml nitrobenzene. A dropping funnel was then used to add Br_2 (0.24 M in nitrobenzene, 205 mmol) to the stirring suspension. After the addition was complete, the yellow suspension was heated at 120 °C for 18 hours and then cooled to RT. The precipitate was then collected by Büchner filtration and washed with a copious amount of ethanol (1.50 L). The residue was dried *in vacuo* to yield 1,3,6,8-tetrabromopyrene (**8**) as a pale yellow-green solid (24.3 g, 94%). The product was found to be insoluble in all common organic solvents, limiting characterisation. Reactivity (borylation or carbonylation) was consistent with the proposed structure. **Elem. Anal**. Calc'd for $C_{16}H_{16}Br_4$ = C, 37.11; H, 1.17; Br, 61.72; Found: C, 37.65; H, 1.20; **FT-IR** (main absorptions): *υ* 1590, 1464, 1452, 1226, 1053, 986, 872, 810, 690, 673 cm^{-1}; **LRMS-MALDI**: *m/z* calculated for $C_{16}H_{16}Br^{79}{}_4{}^+$ $[M]^+$: 513.72, found 513.90 [lit[2] LRMS (EI): m/z (%) = 522 (12), 520 (50), 518 (70), 516 (48), 514 (12) $[M^+]$]

[Di-tert-butyl 3-[2-(tert-Butoxycarbonyl)ethyl]-4-nitroheptanedicarboxylate] (A) [2]

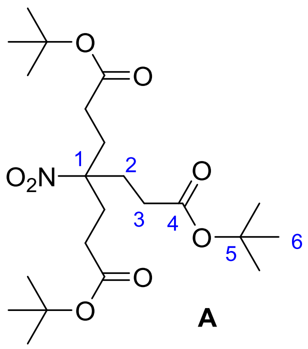

A

A solution of nitromethane (11.4 mL, 200 mmol) in 1,2-dimethoxyethane (50 mL) was prepared and heated to 65 °C before the addition of Triton-B (2.00 mL, 4.40 mmol, 40% in MeOH). Very carefully, tert-Butyl acrylate (92.0 mL, 620 mmol) was then added dropwise (caution exothermic), ensuring the reaction temperature was 75-85 °C. The addition was complete after approx. 40 mins, Triton B (1.00 mL, 2.20 mmol, 40% in MeOH) was then added and the reaction was stirred at 75 °C for 2 hours. After cooling, the solution

was decanted from insolubles and concentrated *in vacuo.* The resulting oil was dissolved in diethyl ether (200 mL), washed with HCl (10% in water, 2 x 100 mL), sat. $NaHCO_3$ (2 x 100 mL) and water (2 x 100 mL). The organics were dried over $MgSO_4$ and solvent removed under reduced pressure to give an amorphous solid. The solid was recrystallised from warm ethanol (150 mL) and the crystals washed with ice-cold methanol (400 mL) and dried to give triester **A** (83.6 g, 188 mmol, 94%) as colourless needles. **^{1}H NMR** (500 MHz, $CDCl_3$) δ = 2.22 – 2.17 (m, 12H, H-2, H-3), 1.43 (s, 27H, H-6) [lit.[2] ^{1}H NMR ($CDCl_3$, 250 MHz) δ = 2.21 (m, 12H), 1.45 (s, 27H)]; **^{13}C NMR** (126 MHz, $CDCl_3$) δ = 171.2 (C-4), 92.3 (C-1), 81.3 (C-5), 30.5 (C-2), 29.9 (C-3), 28.2 (C-6); **LRMS-ESI** [M + Na]$^+$ Calc. for $C_{22}H_{39}NaNO_6$ 468.3, Found 468.3.

^{1}H NMR (500 MHz, $CDCl_3$):

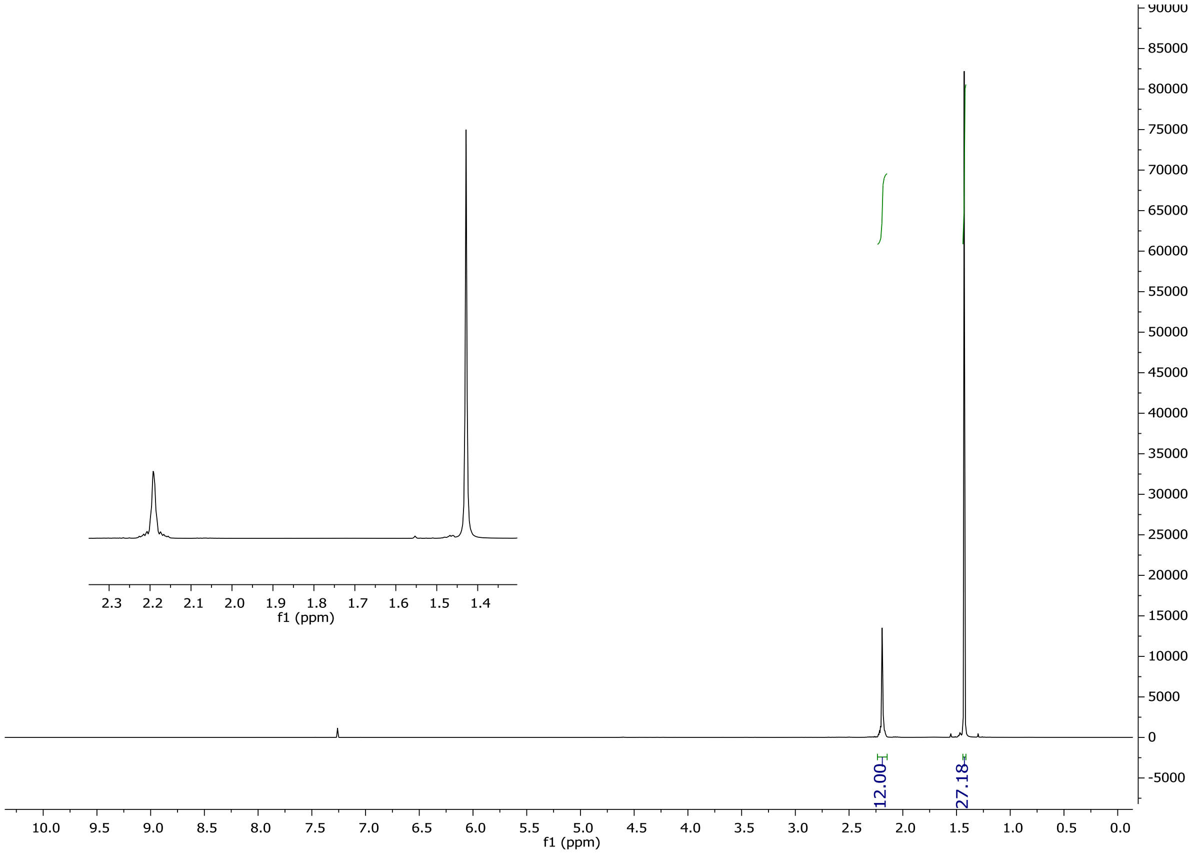

[Tri-tert-butyl 3,3′,3″-(1-aminomethanetriyl)tripropanoate] (Behera's amine) (5) [2]

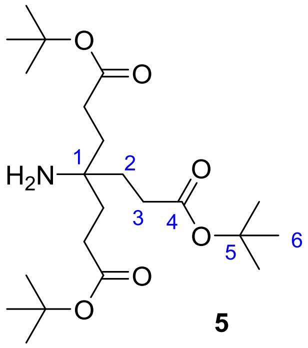

5

An aqueous suspension of Raney Nickel (5 mL) was added to a solution of di-tert-butyl-4-[2-(tert-butoxycarbonyl)ethyl]-4-nitroheptanedicarboxylate **A** (5.00 g, 11.2 mmol) in ethanol (10 mL). This was then pressurised with H_2 (50 bar) and stirred at 50 °C for 18 hours. The suspension was then filtered through Celite, eluting with DCM (200 mL) and MeOH (200 mL). The solvent was then removed from the filtrate under reduced pressure to yield amine **5** (4.40 g, 10.6 mmol, 95%) as a white solid. **^{1}H NMR** ($CDCl_3$, 400 MHz) δ = 2.26 – 2.20 (m, 6H, H-3), 1.63 – 1.55 (m, 6H, H-2), 1.43 (s, 27H, H-6). [lit.[2] ($CDCl_3$, 250 MHz): δ = 1.78 (m, 12H, C*H*$_2$), 1.44 (s, 27H, C*H*$_3$)]; **^{13}C NMR** ($CDCl_3$, 100 MHz) δ = 173.2 (C-4), 80.4 (C-5), 52.5 (C-1), 34.5 (C-3), 30.1 (C-2), 28.2 (C-6); **LRMS-ESI** $[M + H]^+$ Calc. for $C_{22}H_{42}NO_6$ 416.3, Found 416.3.

^{1}H NMR ($CDCl_3$, 400 MHz):

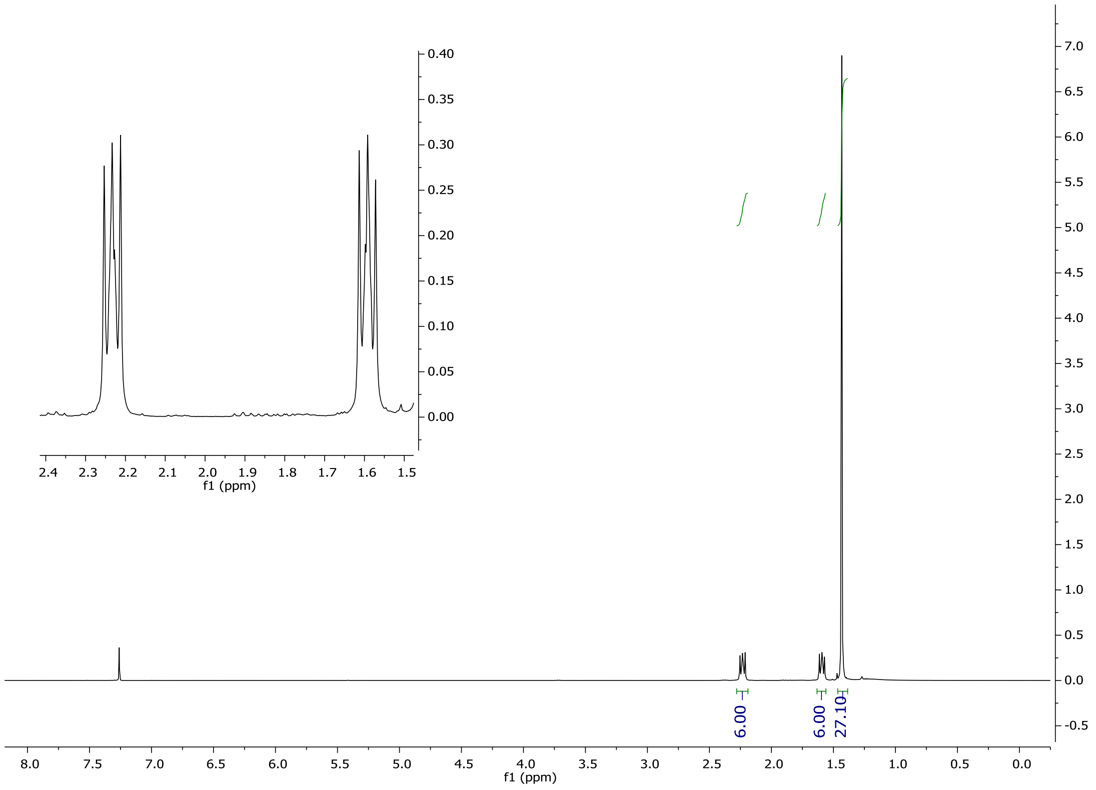

Aryl Bromide 6

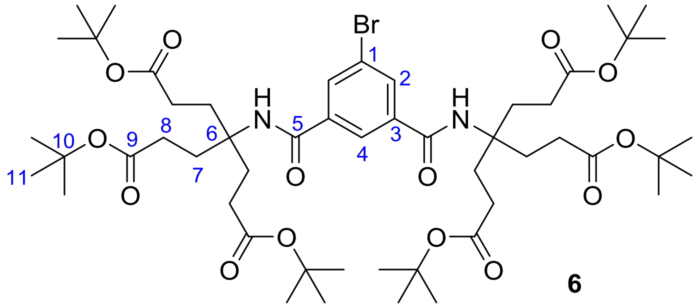

6

5-Bromoisophthalic acid (**4**) (472 mg, 1.93 mmol) was suspended in thionyl chloride (13 mL) and heated to reflux. After 3 hours the reaction was cooled and the volatiles removed under reduced pressure, giving the acyl chloride as brown oil. To this was added a solution of Behera's amine **5** (2.00 g, 4.81 mmol) and DIPEA (1 mL) in anhydrous THF (7 mL). The reaction was stirred for 17 hours, before concentration in vacuo and purification by column chromatography (DCM/MeOH, 99:1) to give the aryl bromide **6** (1.05 mg, 53%) as a white solid. $\boldsymbol{R_f}$ (EtOAc/MeOH, 9:1) 0.55; **^{1}H NMR** ($CDCl_3$, 400 MHz): δ = 8.23 (t, *J* = 1.5 Hz, 1H, H-4), 8.09 (d, *J* = 1.5 Hz, 2H, H-2), 7.34 (s, 2H, NH), 2.30 (t, *J* = 7.6 Hz, 12H, H-8), 2.11 (t, *J* = 7.6 Hz, 12H, H-7), 1.43 (s, 54H, H-11); **^{13}C NMR** ($CDCl_3$, 126 MHz): δ = 173.3 (C-9), 164.5 (C-5), 137.1 (C-3), 132.9 (C-2), 124.5 (C-4), 123.0 (C-1), 81.1 (C-10), 58.3 (C-6), 30.4 (C-7), 30.1 (C-8), 28.2 (C-11); **FT-IR** (main absorptions): υ 3349, 3180, 2978, 2933, 1728, 1652, 1537, 1367, 1151, 954, 849 758 cm^{-1}; **HRMS-ESI** $[M + Na]^+$ Calc. for $C_{52}H_{83}{}^{79}BrN_2NaO_{14}$ 1061.4925, Found 1061.4953.

^{1}H NMR ($CDCl_3$, 400 MHz):

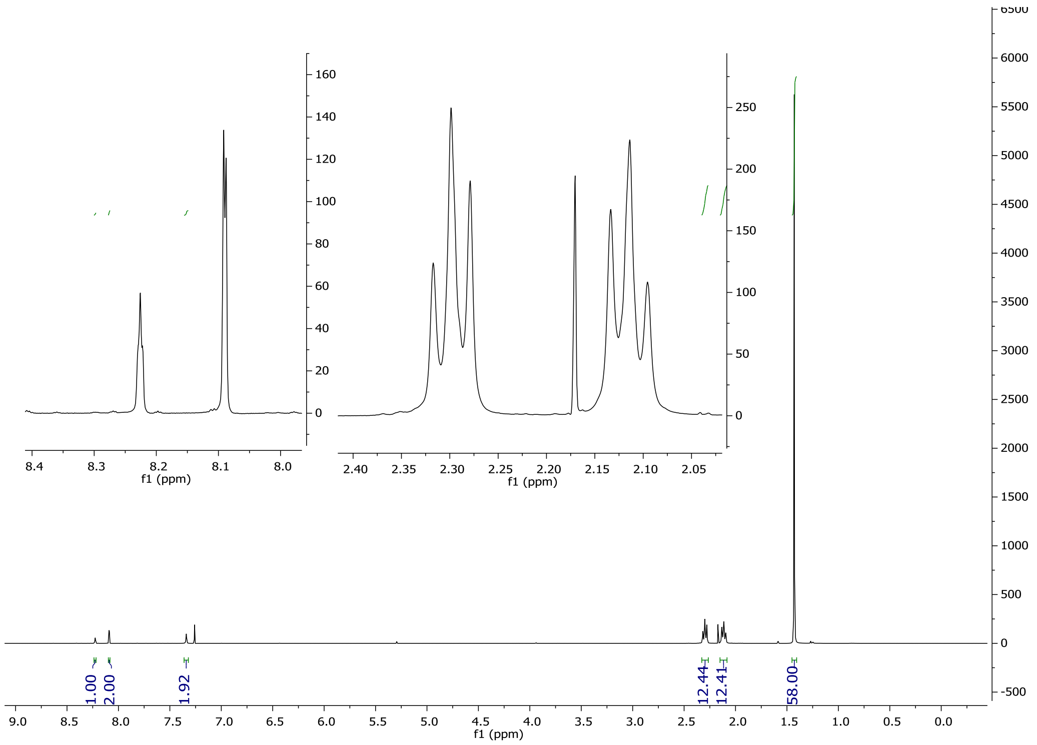

Aryl Boronate 7

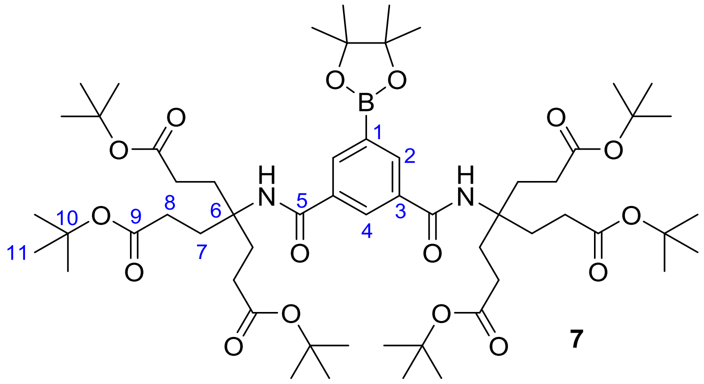

A solution of aryl bromide **6** (1.00 g, 0.96 mmol), Pd(dppf)Cl_2 (21.1 mg, 28.8 µmol), bis(pinocolato)diboron (0.37 g, 1.44 mmol) and potassium acetate (0.28 g, 2.89 mmol) in degassed anhydrous dioxane (5 mL) was heated to 80 °C, sealed and stirred for 3 days. The solvent was then removed under reduced pressure and the residue purified by column chromatography (DCM/MeOH, 98.5:1.5) to give the crude product as an orange foam. The excess B_2Pin_2 was then sublimed onto a cold finger by heating to 60 °C under high vacuum, leaving the boronate **7** (0.84 g, 0.77 mmol, 80%) as an orange solid. **^{1}H NMR** ($CDCl_3$, 301 MHz) δ 8.38 (t, *J* = 1.8 Hz, 1H, H-4), 8.31 (d, *J* = 1.8 Hz, 2H, H-2), 6.76 (s, 2H, NH), 2.28 (t, *J* = 7.6 Hz, 12H, H-8), 2.11 (t, *J* = 7.6 Hz, 12H, H-7), 1.43 (s, 58H, H-11), 1.36 (s, 12H, BPin); **^{13}C NMR** (101 MHz, $CDCl_3$) δ 172.3 (C=O), 165.8 (C=O), 135.7 (C-2), 128.7 (C-4), 84.5 (Bpin), 80.9 (C-10), 58.2 (C-6), 30.1 (C-7), 30.0 (C-8), 28.2 (C-11), 25.0 (Bpin); **FT-IR** (main absorptions): υ 3357, 2979, 2934, 1726, 1666, 1526, 1367, 1145, 966, 847, 736, 703 cm^{-1}; **HRMS-ESI** $[M + Na]^+$ Calc. for $C_{58}H_{95}BN_2NaO_{16}$ 1109.6672, Found 1109.6667.

^{1}H NMR ($CDCl_3$, 301 MHz):

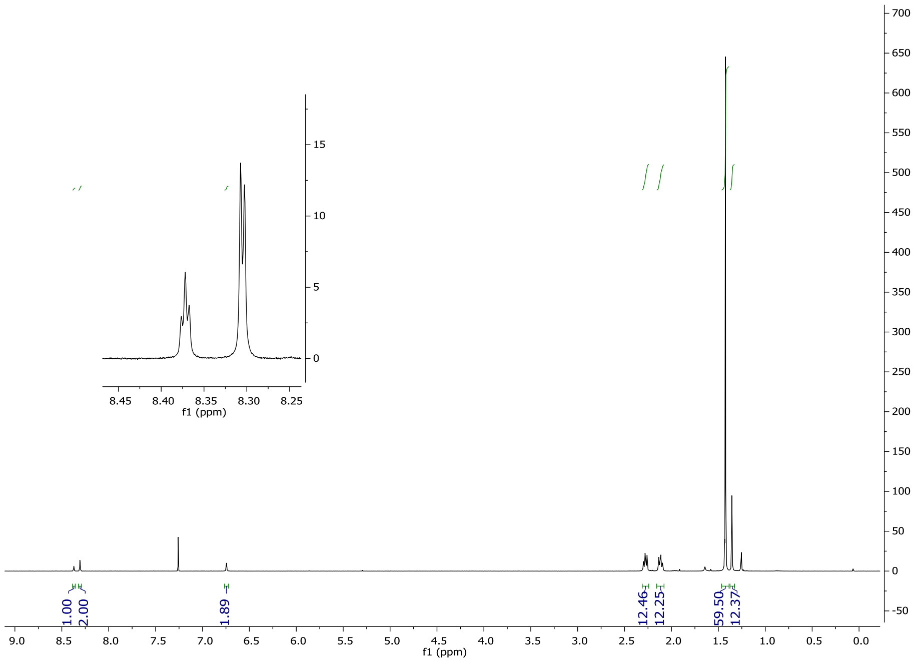

O-Protected Receptor B

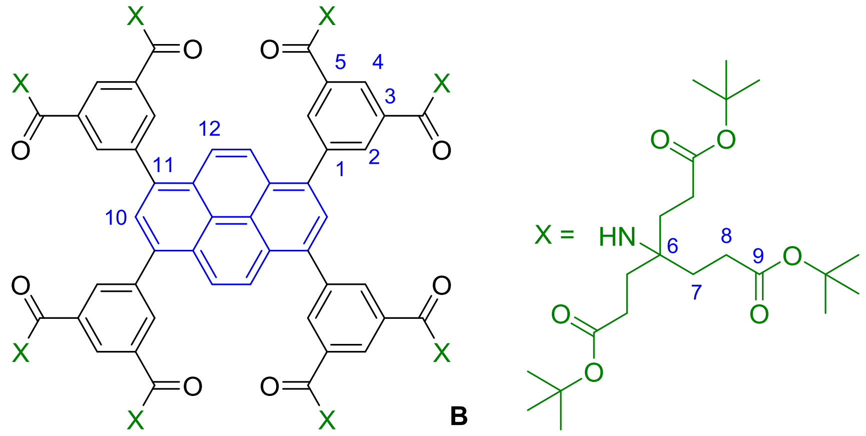

A solution of 1,3,6,8-tetrabromopyrene **8** (7.9 mg, 0.0153 mmol), aryl boronate **7** (100 mg, 0.092 mmol), Pd(dppf)Cl_2 (0.34 mg, 0.46 µmol) and caesium carbonate (50.0 mg, 0.153 mmol) in degassed dioxane (1.5 mL) and water (200 µL) was prepared. The flask was then sealed before heating to 90 °C and stirring for 4 days. The solvent was removed under reduced pressure and the residue purified by prep-HPLC (Flow rate: 17 mL/min, Run: [H_2O/Acetone 15:85]→45min→[Acetone], UV/Vis: 254 nm, 214 nm, 334 nm, Product = 23 min) to give the pyrene **B** (33.0 mg, 53%) as a colourless solid. **^{1}H NMR** ($CDCl_3$, 500 MHz) δ 8.39 (t, *J* = 1.6 Hz, 4H, H-4), 8.19 (d, *J* = 1.6 Hz, 8H, H-2), 8.11 (s, 4H, H-12), 8.02 (s, 2H, H-10), 7.31 (s, 8H, NH), 2.29 (t, *J* = 7.7 Hz, 48H, H-8), 2.12 (t, *J* = 7.6 Hz, 48H, H-7), 1.32 (s, 216H, C(C$\underline{H}_3$)$_3$); **^{13}C NMR** ($CDCl_3$, 126 MHz) δ 173.13 (C-9), 165.52 (C-5), 141.71 (*q*-C), 136.14 (*q*-C), 135.60 (*q*-C), 131.95 (C-2), 128.71 (C-10), 125.90 (C-12), 124.70 (C-4), 80.85 ($\underline{C}$(CH_3)$_3$), 58.18 (C-6), 30.41 (C-8), 30.09 (C-7), 28.13 (C($\underline{C}H_3$)$_3$); **HRMS-ESI** [M + 3Na]$^{3+}$ Calc. for $C_{224}H_{338}N_8O_{56}Na_3$ 1368.4513, Found 1368.4466.

^{1}H NMR ($CDCl_3$, 500 MHz):

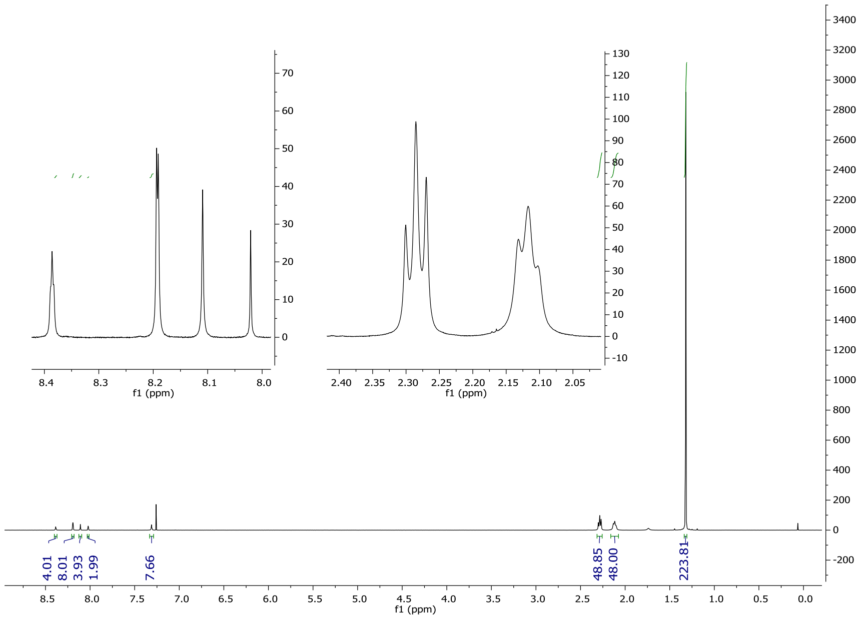

Anionic Receptor 9

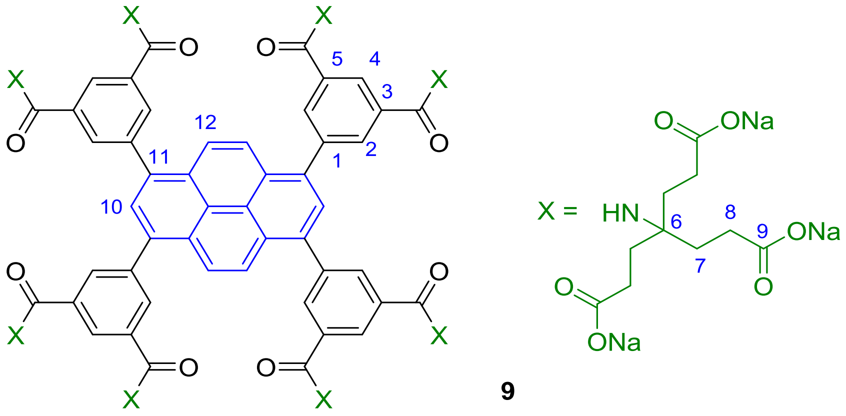

O-Protected receptor **B** (62.0 mg, 15.4 µmol) was stirred in TFA (2.00 mL, 26 mmol), triethylsilane (70 µL, 0.92 mmol) and DCM (3 mL) for 18 hours before the volatiles were removed under reduced pressure. The resulting film was then dissolved in a small amount of methanol (0.1 mL) and precipitated by addition of water (5 mL), the suspension was then freeze dried. The resulting solid was then dissolved in water (3 mL) and neutralised with NaOH before freeze drying to yield receptor **9** (49.6 mg, 15.4 µmol, quant.) as a colourless solid. **^{1}H NMR** (500 MHz, D_2O) δ 8.22 (s, 4H, H-12), 8.20 (s, 4H, H-4), 8.16 (s, 8H, H-2), 8.14 (s, 2H, H-10), 2.33 (t, *J* = 7.9 Hz, 48H, H-8), 2.14 (t, *J* = 7.9 Hz, 48H, H-7); **^{13}C NMR** (126 MHz, D_2O) δ = 182.4 (C-9), 169.6 (C-5), 141.3 (*q*-C), 136.1 (*q*-C), 132.0 (C-2), 129.6 (C-10), 128.4 (C-q), 125.7 (C-12), 125.1 (C-4), 124.9 (C-q), 59.2 (C-6), 31.4 (C-8), 30.4 (C-7).

^{1}H NMR (500 MHz, D_2O):

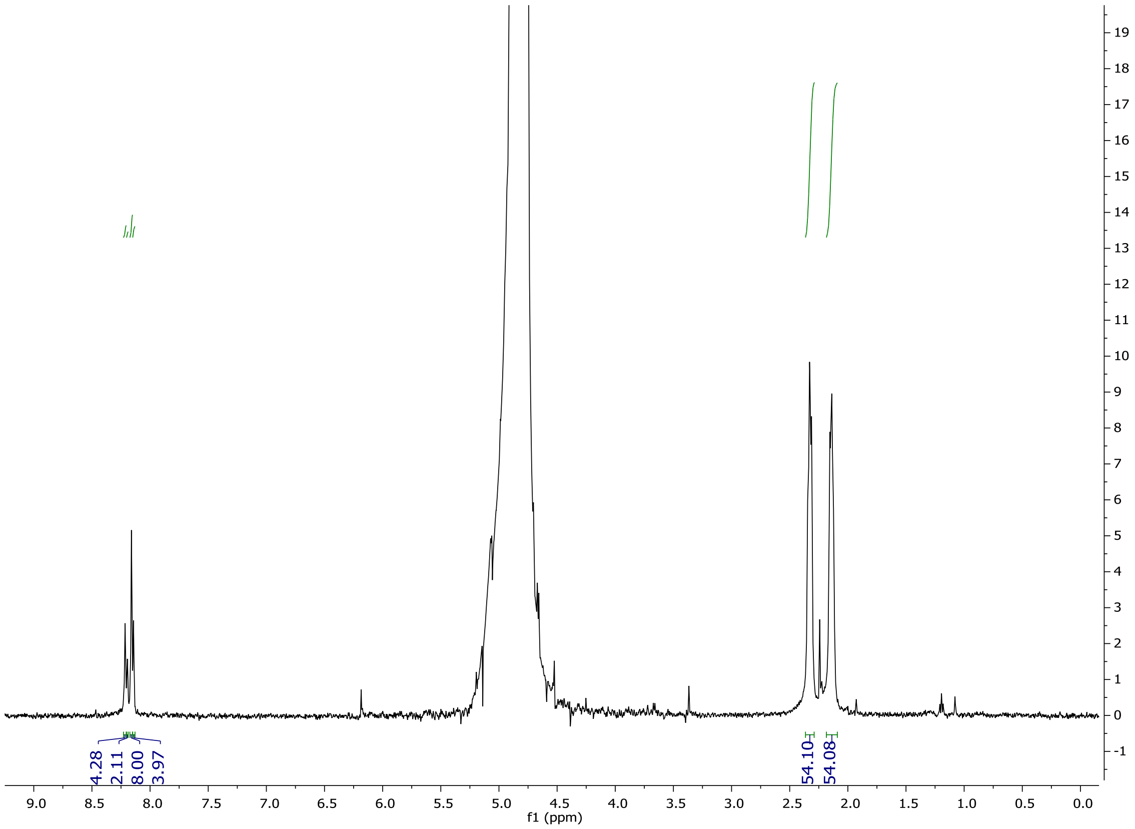

Cationic Receptor 16

3-(2-Aminoethyl)-3-nitropentane-1,5-diamine.3HCl (C) [3]

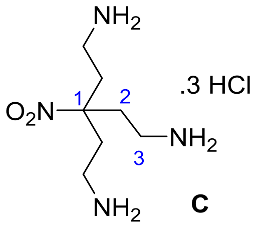

C

Nitromethanetrispropionic acid (8.88 g, 32.0 mmol) and DMF (4 drops) were dissolved in thionyl chloride (60 mL) and refluxed for 2 hours. The volatiles were then removed under high vacuum at 70 °C for 1 hour before cooling to RT and dissolution in dioxane (60 mL). Trimethylsilylazide (12.6 mL, 96 mmol) was then added, caution was taken as gas evolved. While keeping the gas evolution at acceptable levels, the temperature was gradually increased to 80 °C, after which the reaction was stirred for 30 min. The reaction was then cooled to 45 °C before the addition of acetone (100 mL) and slow addition of HCl (conc., 25 mL), upon which gas was evolved. After stirring for 1 hour the reaction mixture was filtered and the filtrand washed with cold acetone (50 mL) giving the triamine **C** (10.4 g, 32.0 mmol, quant.) as a tan solid. The filtrate was quenched by addition of ceric ammonium nitrate. **^{1}H NMR** (301 MHz, DMSO-D_6) δ = 8.33 (br s, 9H, NH), 2.80 (br s, 6H, H-2), 2.31 (br m, 6H, H-3) [lit.[3] (DMSO-D_6) δ = 8.39 (s, 9H), 2.81 (t, 6H), 2.33 (t, 6H)]; **^{13}C NMR** (101 MHz, DMSO-D_6) δ = 89.4 (C-1), 33.8 (C-2), 31.8 (C-3); **LRMS-ESI** [M +H -3(HCl)]$^+$ Calc. for $C_7H_{19}N_4O_2$ 191.1, Found 191.2.

^{1}H NMR (301 MHz, DMSO-D_6):

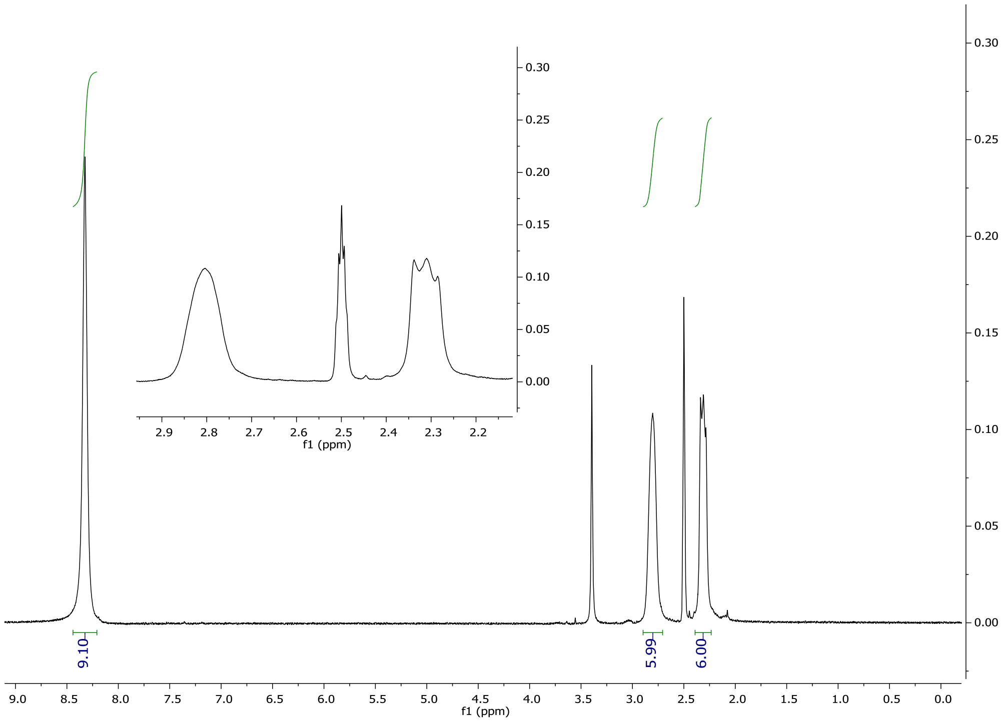

Nitromethanetris(2-tert-butoxycarbonylaminoethane) (D) [3]

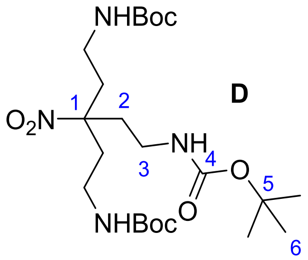

Di-tert-butyl dicarbonate (5.00 g, 23.0 mmol) was added to a solution of 3-(2-aminoethyl)-3-nitropentane-1,5-diamine.3HCl **C** (1.50 g, 5 mmol) in triethylamine (12.5 mL) and acetonitrile (37.5 mL). The solution was heated to reflux and stirred for 24 hours. The reaction mixture was then diluted with ethyl acetate (75 mL) and washed with water (75 mL). The aqueous phase was then extracted with ethyl acetate (75 mL) and the combined organic phases dried over $MgSO_4$. The suspension was then filtered and the solvent removed under reduced pressure to give the tris-carbamate **D** (2.42 g, 4.93 mmol, 99%) as a white solid. **^{1}H NMR** (400 MHz, $CDCl_3$) δ = 4.80 (brs, NH), 3.15 (q, *J* = 7.1 Hz, 6H, H-3), 2.18 (t, *J* = 7.4 Hz, 6H, H-2), 1.43 (s, 27H, H-6) [lit.[3] ($CDCl_3$) δ = 4.81 (s, 3H), 3.13 (m, 6H), 2.16 (t, 6H), 1.40 (s, 27H)]; **^{13}C NMR** (101 MHz, $CDCl_3$) δ 156.0 (C-4), 90.7 (C-1), 79.8 (C-5), 36.0 (C-3), 35.8 (C-2), 28.5 (C-6); **HRMS-ESI** $[M + Na]^+$ Calc. for $C_{22}H_{42}N_4NaO_8$ 513.2895, Found 513.2884.

^{1}H NMR (400 MHz, $CDCl_3$):

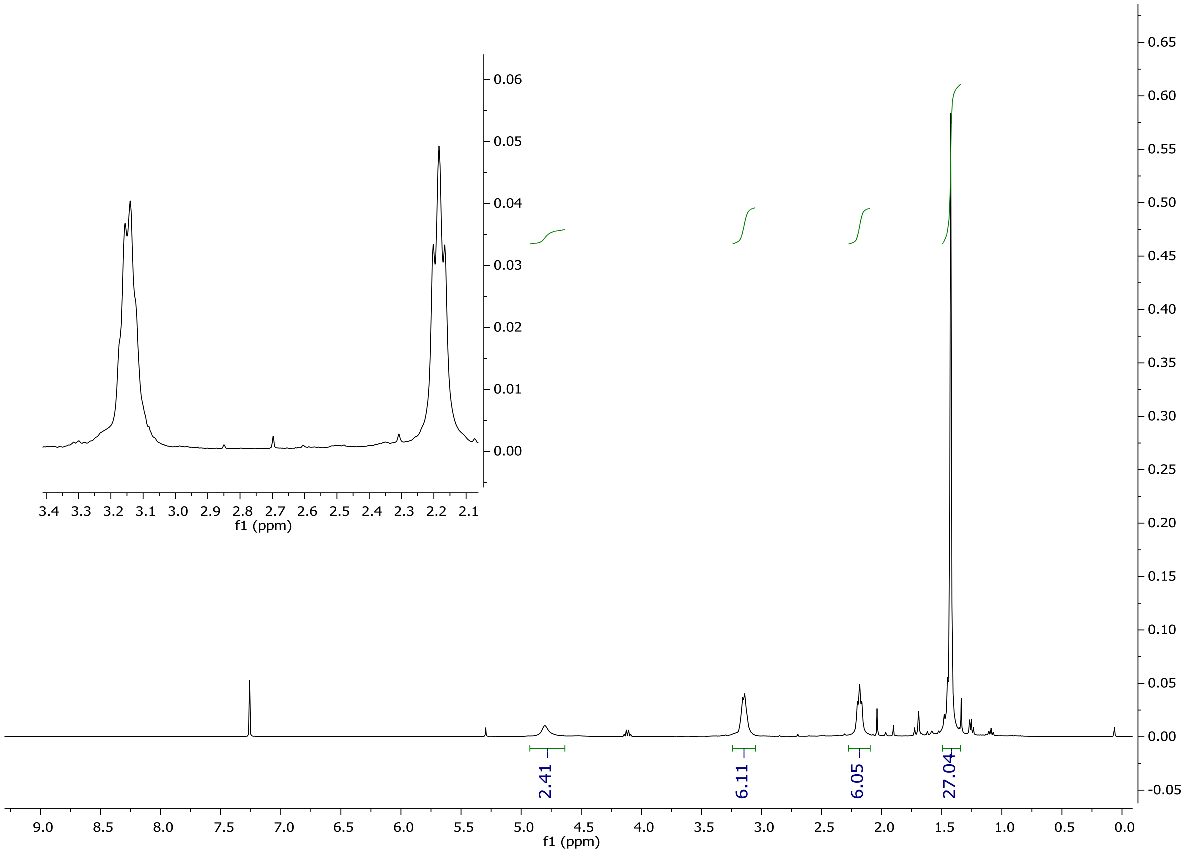

Aminomethanetris(2-tert-butoxycarbonylaminoethane) (E) [3]

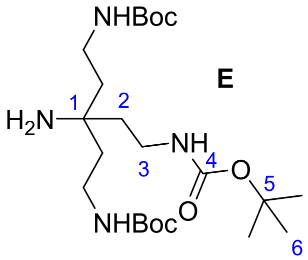

An aqueous suspension of Raney-Nickel (50%, 5 mL) was added to a solution of nitromethanetris(2-tert-butoxycarbonylaminoethane) **D** (1.80 g, 3.67 mmol) in ethanol (10 mL). The reaction mixture was then pressurised with H_2 (50 bar) and heated to 50 °C. After stirring for 18 hours the reaction was vented and cooled before filtering through Celite, eluting with DCM (200 mL) and methanol (200 mL). The solvent was then removed under reduced pressure to yield the amine **E** (1.55 g, 3.37 mmol, 92%) as a glassy colourless solid. **^{1}H NMR** (400 MHz, CD_3OD) δ = 3.18 – 3.05 (m, 6H, H-3), 1.64 – 1.53 (m, 6H, H-2), 1.43 (s, 27H, H-6) [lit.[3] ($CDCl_3$) δ = 5.06 (s, 3H), 3.18 (m, 6H), 1.78 (s, 2H), 1.56 (t, 6H), 1.41 (s, 27H)]; **^{13}C NMR** (126 MHz, CD_3OD) δ = 158.5 (C-4), 80.1 (C-5), 53.7 (C-1), 39.2 (C-3), 36.4 (C-2), 28.8 (C-6); **HRMS-ESI** $[M + H]^+$ Calc. for $C_{22}H_{45}N_4O_6$ 461.3334, Found 461.3340.

^{1}H NMR (400 MHz, CD_3OD):

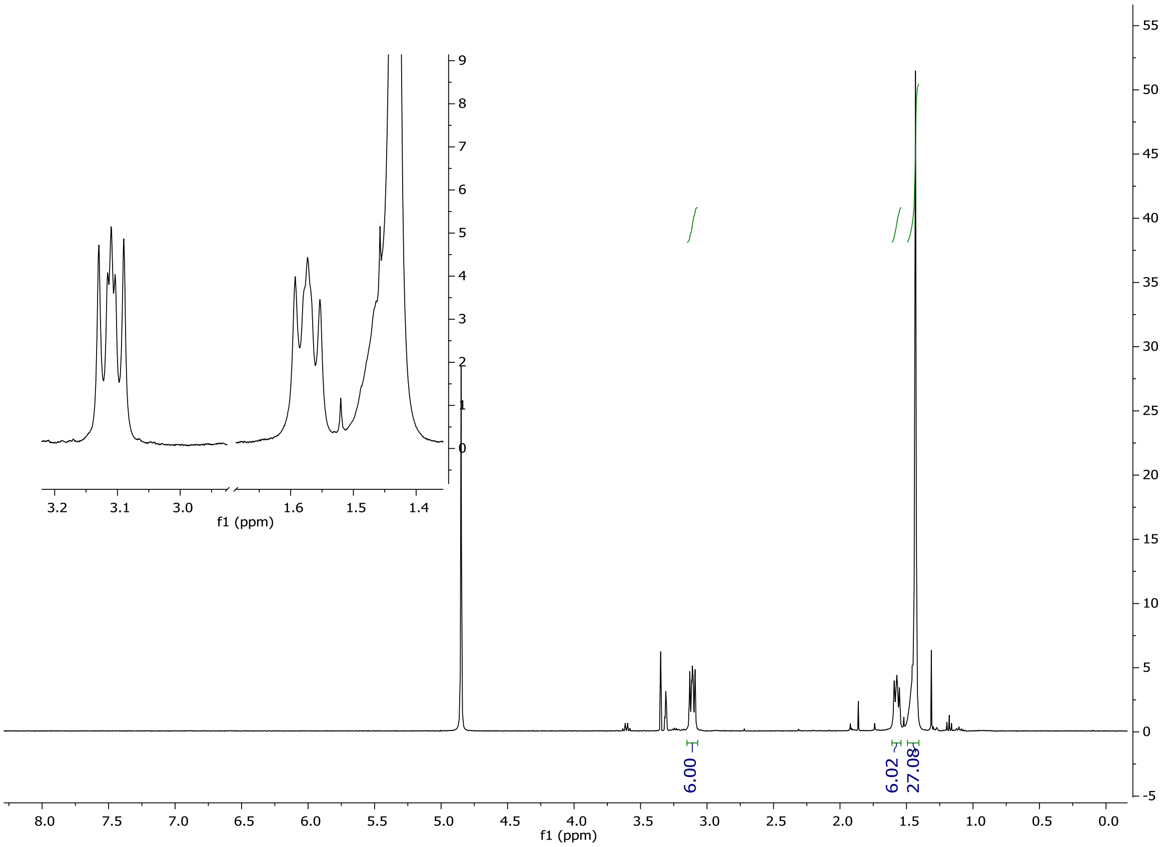

***N*-Boc Protected Aryl Bromide F**

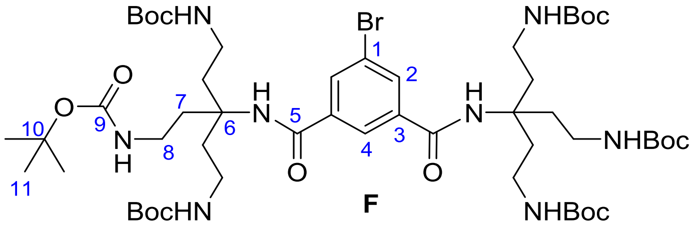

5-Bromoisophthalic acid **4** (0.11 g, 0.45 mmol) was dissolved in thionyl chloride (5 mL) and DMF (3 drops) before stirring at reflux for 3 hours. The volatiles were then removed under high vacuum at 60 °C (≈ 1 hour). In a separate flask a solution of aminomethanetris(2-tert-butoxycarbonylaminoethane) **E** (0.50 g, 1.09 mmol) and DIPEA (0.37 mL, 2.25 mmol) in DCM (2 mL) was prepared. Both flasks were cooled to 0 °C before the addition of the amine solution to the acyl chloride. This was then warmed to RT after 30 min and stirred for 48 hours. The volatiles were then removed under reduced pressure and the residue purified by column chromatography (MeOH/DCM, 2.5:97.5) to give the aryl bromide **F** (0.28 g, 0.25 mmol, 56%) as a white solid. R_f = 0.20 (MeOH/DCM, 2.5:97.5); **^{1}H NMR** (400 MHz, CD_3OD) δ 8.13 (br s, 3H, H-2, H-4), 3.23 – 3.05 (m, 12H, H-8), 2.18 – 1.98 (m, 12H, H-7), 1.42 (s, 54H, H-11); **^{13}C NMR** (101 MHz, $CDCl_3$) δ 156.4 (C-9), 133.5 (C-2), 131.6 (C-4), 79.6 (C-10), 57.4 (C-6), 36.2 (C-8), 36.1 (C-7), 28.4 (C-11); **FT-IR** (main absorptions): υ 3322, 2976, 2934, 1682, 1515, 1365, 1248, 1164, 960, 867, 720 cm^{-1}; **HRMS-ESI** $[M + Na]^+$ Calc. for $C_{52}H_{89}{}^{79}BrN_8O_{14}Na$ 1151.5574, Found 1151.5560.

^{1}H NMR (400 MHz, CD_3OD):

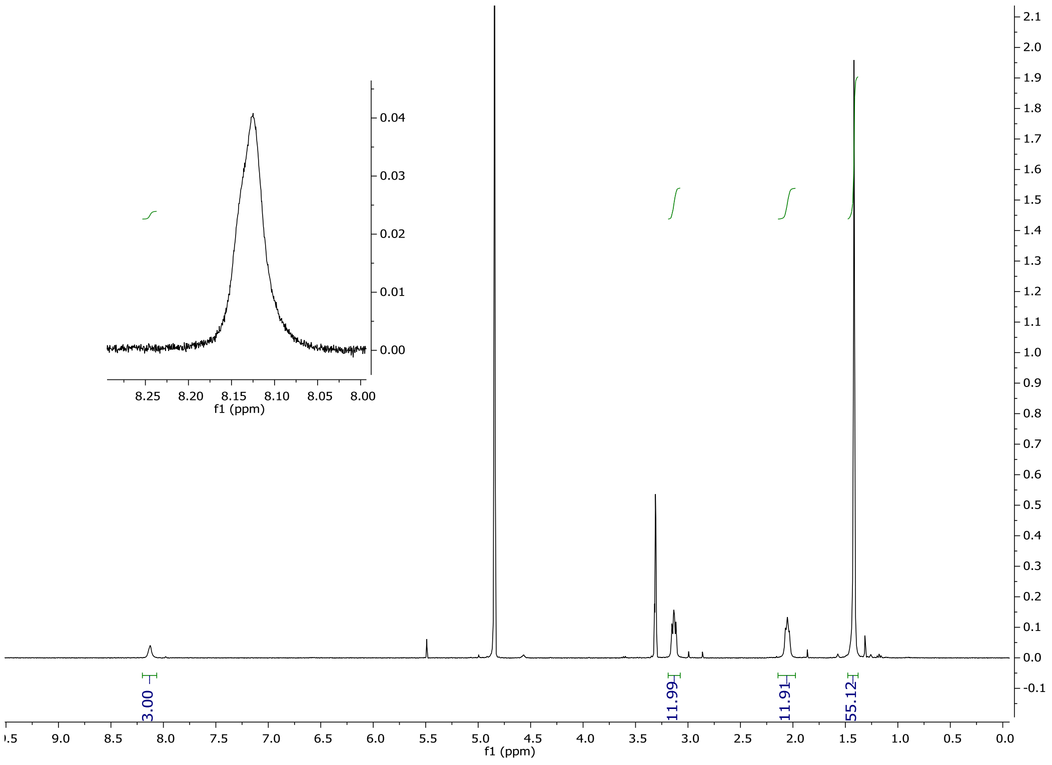

N-Boc Protected Aryl Boronate G

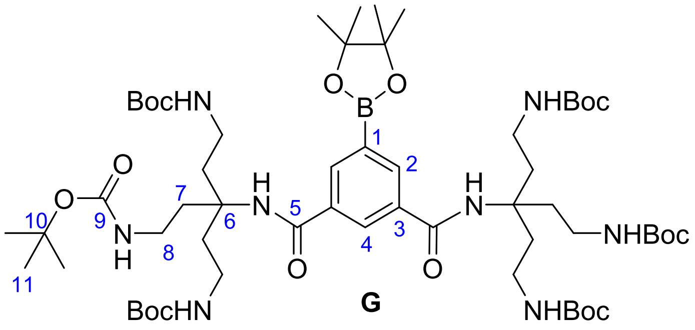

A solution of aryl bromide **F** (2.00 g, 1.82 mmol), $Pd_2(dba)_3$ (25.0 mg, 27.3 µmol), X-Phos (52.0 mg, 109 µmol), bis(pinocolato)diboron (1.38 g, 5.44 mmol) and potassium acetate (0.53 g, 5.44 mmol) in degassed anhydrous dioxane (5 mL) was stirred for 30 min. The solution was then heated to 80 °C, sealed and stirred for 24 hours. The solvent was then removed under reduced pressure and the residue dissolved in methanol (40 mL), filtered and washed with heptane (6 x 40 mL). The solvent was then removed from the methanolic fraction before purification by column chromatography (DCM/MeOH, 95:5). The crude product was then dissolved in methanol (40 mL) and further washed with heptane (3 x 40 mL). The methanolic fraction was then concentrated to dryness, giving the boronate **G** (1.39 g, 1.18 mmol, 65%) as a colourless solid. **^{1}H NMR** (400 MHz, MeOD) δ 8.27 (s, 2H, H-2), 8.22 (s, 1H, H-4), 6.56 (s, NH), 3.15 (t, J = 9.4, 6.2 Hz, 12H, H-8), 2.07 (t, J = 9.5, 6.0 Hz, 12H, H-7), 1.41 (s, 54H, H-11), 1.37 (s, 12H, BPin); **^{13}C NMR** (101 MHz, $CDCl_3$) δ 169.7 (C-5), 158.4 (C-9), 137.3 (C-2), 136.6 (C-3), 130.2 (C-4), 85.7 (BPin), 80.0 (C-10), 58.4 (C-6), 36.7 (C-8), 35.9 (C-7), 28.8 (C-11), 25.3 (Bpin); **FT-IR** (main absorptions): υ 3444, 2978, 2934, 2487, 1683, 1423, 1367, 1162, 984, 855, 780 cm^{-1}; **HRMS-ESI** $[M + Na]^+$ Calc. for $C_{58}H_{101}BN_8O_{16}Na$ 1199.7331, Found 1199.7324.

^{1}H NMR (400 MHz, MeOD):

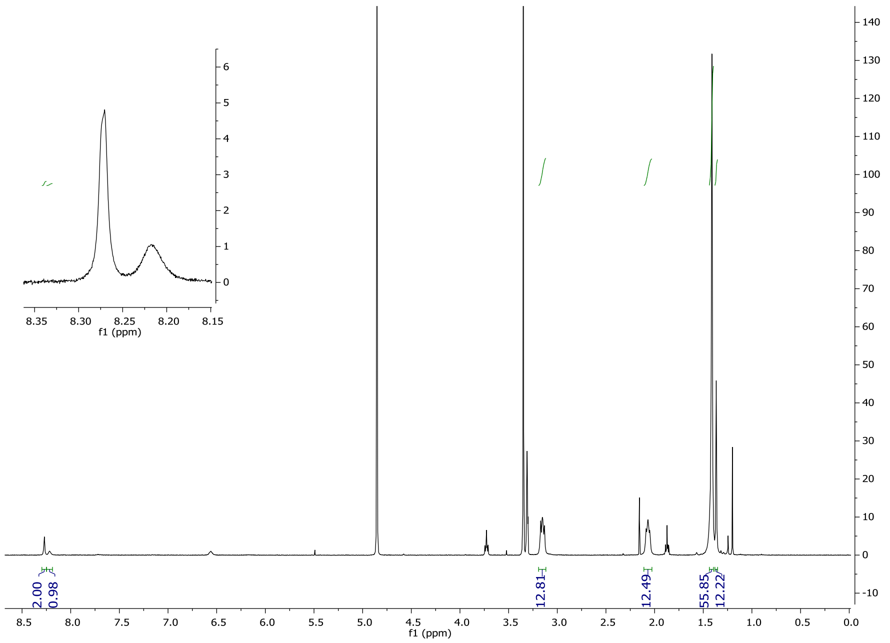

***N*-Boc Protected Tetracosa-amine H**

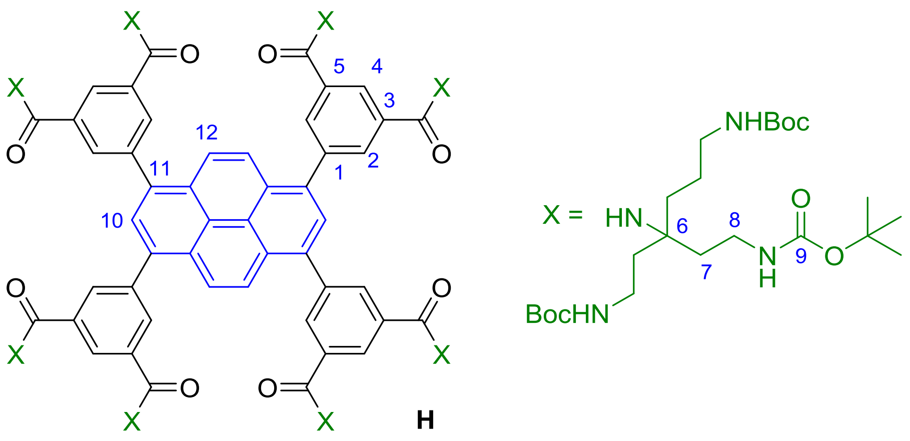

A solution of 1,3,6,8-tetrabromopyrene **8** (29.3 mg, 56.6 µmol), aryl boronate **G** (400 mg, 0.34 mmol), Pd(dppf)Cl_2 (1.24 mg, 1.70 µmol) and caesium carbonate (186 mg, 0.57 mmol) in degassed dioxane (6 mL) and water (200 µL) was prepared. The flask was then sealed before heating to 80 °C and stirring for 4 days. The solvent was removed under reduced pressure and the residue purified by prep-HPLC (Flow rate: 20 mL/min, Run: [H_2O/Acetone 20:80]→30min→[H_2O/Acetone 10:90], UV/Vis: 238 nm, 340 nm, 380 nm) to give the pyrene **H** (166 mg, 66%) as a colourless solid. **^{1}H NMR** (400 MHz, CD_3OD) δ 8.35 (s, 4H, H-4), 8.30 (s, 8H, H-2), 8.20 (s, 4H, H-12), 8.15 (s, 2H, H-10), 3.15 (t, *J* = 9.0, 5.9 Hz, 48H, H-8), 2.13 – 2.01 (m, 48H, H-7), 1.31 (s, 216H, C(C<u>H</u>$_3$)$_3$); **^{13}C NMR** (126 MHz, MeOD) δ 169.0 (C-5), 158.4 (C-9), 142.5 (C-1), 137.7 (*q*-C), 137.3 (C-3), 133.3 (C-2), 131.1 (C-10), 129.9 (C-11), 127.1 (*q*-C), 126.8 (C-12), 126.8 (C-4), 79.9 (<u>C</u>(CH_3)$_3$), 58.5 (C-6), 36.7 (C-8), 36.3 (C-7), 28.9 (C(<u>C</u>H$_3$)$_3$); **HRMS-ESI** [M + 3Na]$^{3+}$ Calc. for $C_{224}H_{362}N_{32}O_{56}Na_3$ 1489.2074, Found 1489.2069.

^{1}H NMR (400 MHz, CD_3OD):

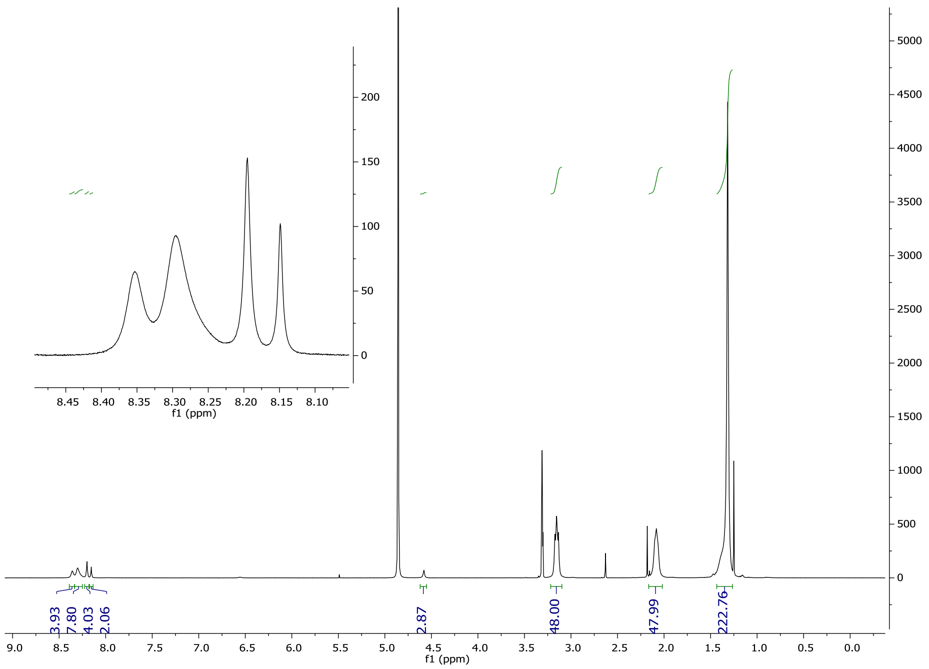

N-Boc Protected Receptor I

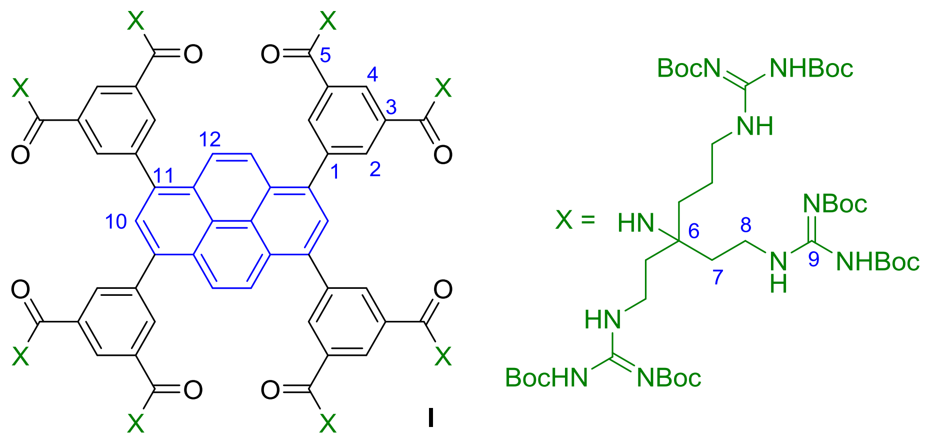

I

A solution of **H** (23.2 mg, 5.28 µmol) in DCM (2 mL) and TFA (0.5 mL) was stirred for 5 hours before the volatiles were evaporated under a flow of nitrogen. The crude material was then co-evaporated with chloroform (3 x 3mL) and freeze dried from water (2 x 3 mL) to give the amine TFA salt. (^{1}H NMR (400 MHz, CD_3OD) δ = 8.36 (t, *J* = 1.7 Hz, 4H, H-4), 8.27 (d, *J* = 1.6 Hz, 8H, H-2), 8.11 (s, 2H, H-10), 8.10 (s, 4H, H-12), 3.16 – 3.05 (m, 48H, H-8), 2.33 (t, *J* = 8.5 Hz, 48H, H-7)). The solid was suspended in sodium hydroxide (1 mL, 5.50 mg/ml in H_2O) and stirred for 2 hours, the water was then removed by freeze drying. The resulting solid was dissolved in water (0.8 mL), then dioxane (4 mL) and 1,3-di-Boc-2-(trifluoromethylsulfonyl)guanidine (200 mg, 0.51 mmol) were added in portions ensuring a clear solution was maintained. The reaction was stirred for 10 minutes before the addition of triethylamine (0.20 mL), the mixture was then stirred for a further 5 days. The volatiles were evaporated under a flow of nitrogen and the crude material purified by prep-HPLC (Flow rate: 17 mL/min; Run: [H_2O/Acetone 20:80]→50min→[H_2O/Acetone 7:93] →40min→[H_2O/Acetone 5:95]; UV/Vis: 340 nm, 380 nm; product @ 78 min) to give the pyrene **I** (12.5 mg, 30%) as a colourless solid. **^{1}H NMR** (400 MHz, CD_3OD) δ 8.85 (s, 4H, NH), 8.56 (s, 4H, H-4), 8.43 (s, 8H, H-2), 8.28 (s, 4H, H-12), 8.09 (s, 2H, H-10), 3.53 (s, 48H, H-8), 2.25 (s, 48H, H-7), 1.42 (s, 216H, C(C<u>H</u>$_3$)$_3$), 1.32 (s, 216H, C(C<u>H</u>$_3$)$_3$); **^{13}C NMR** (126 MHz, CD_3OD) δ 168.6 (C-5), 164.4 (C-9), 157.3 (N(<u>C</u>O)O), 153.0 (N(<u>C</u>O)O), 142.0 (C-1), 137.4 (C-3), 134.4 (C-2), 131.3 (C-10), 129.4 (C-11), 127.0 (C-11a), 127.8 (C-4), 126.4 (C-12), 84.3 (<u>C</u>(CH$_3$)$_3$), 80.2 (<u>C</u>(CH$_3$)$_3$), 37.5 (H-8), 35.9 (H-7), 28.7 (C(<u>C</u>H$_3$)$_3$), 28.4 (C(<u>C</u>H$_3$)$_3$); **HRMS-ESI** [M + 4Na]$^{4+}$ Calc. for $C_{368}H_{602}N_{80}O_{104}Na_4$ 1975.5995, Found 1975.5963.

^1H NMR (400 MHz, CD_3OD):

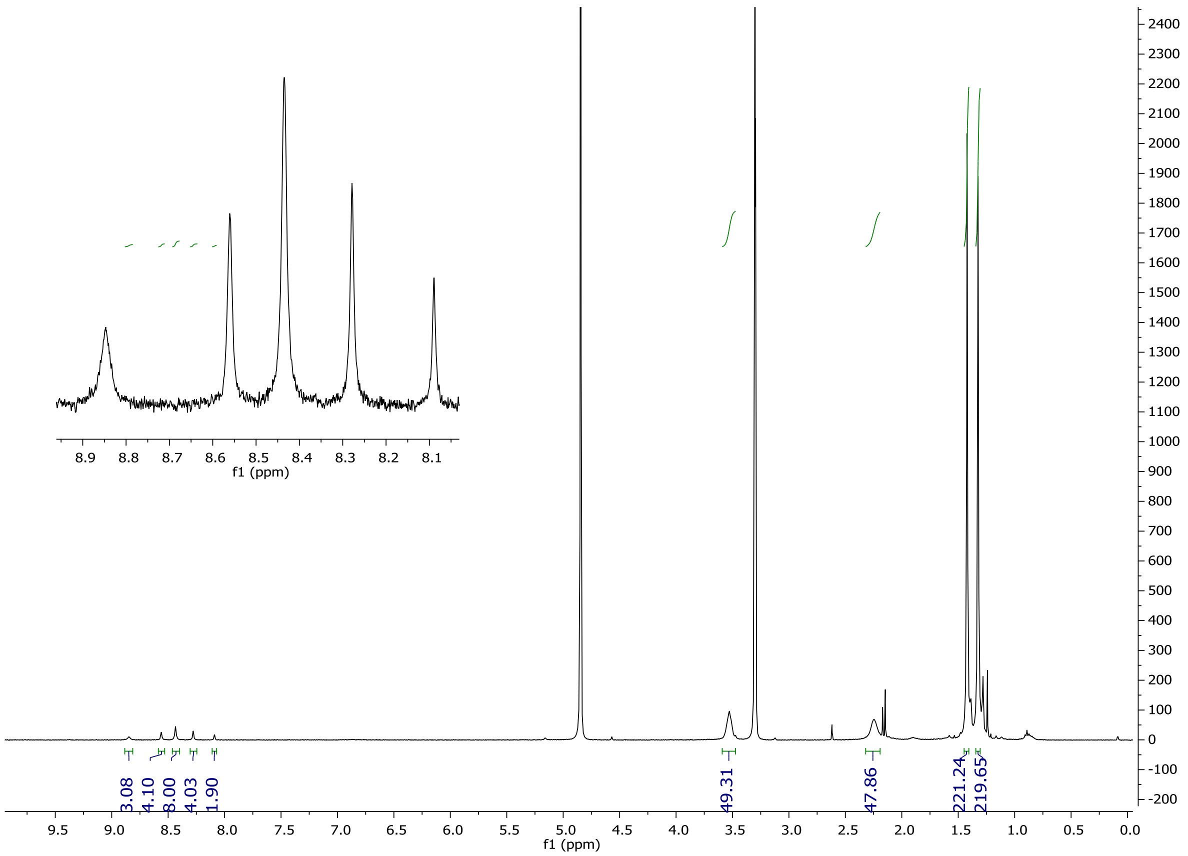

Cationic Receptor 16

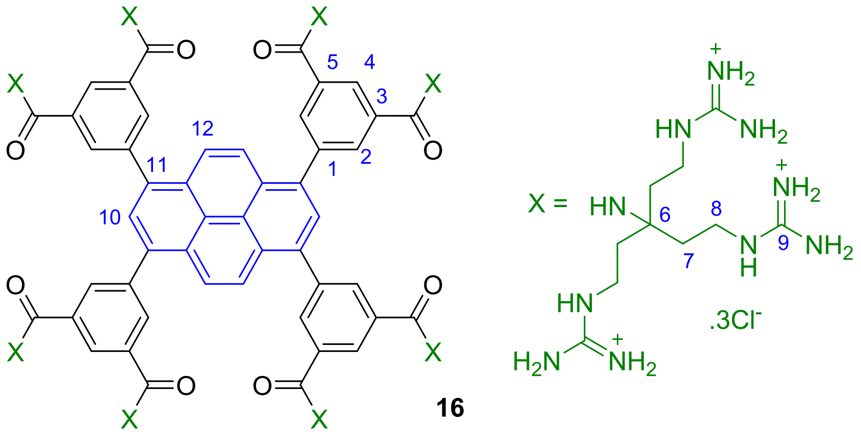

16

N-Boc protected receptor **I** (10.6 mg, 1.36 µmol) was suspended in aqueous hydrochloric acid (1 mL, 6 M) and methanol (1 mL). The mixture was stirred overnight then the volatiles evaporated under a flow of nitrogen. The solid was dissolved in water (1.5 mL) and Amberlyst A26 hydroxide form resin was added portionwise until pH > 7 (pH meter). The mixture was further stirred gently for 15 minutes before HCl (0.04 or 0.40 M in H_2O) was added gradually until pH = 7. The resin was removed by filtration and the solution freeze dried to give the receptor **16** (4.8 mg, 91%) as a colourless solid. **^{1}H NMR** (500 MHz, D_2O) δ 8.37 (s, 4H, H-4), 8.26 (s, 8H, H-2), 8.24 (s, 2H, H-10), 8.21 (s, 4H, H-12), 3.38 (t, *J* = 7.7 Hz, 48H, H-8), 2.30 (t, *J* = 7.7 Hz, 48H, H-7); **^{13}C NMR** (126 MHz, D_2O) δ 174.3 (C-5), 161.4 (C-9), 146.1 (C-1), 140.7 (C-3), 137.5 (C-2), 134.4 (C-10), 133.2 (C-11), 130.5 (C-4), 130.4 (C-12), 62.1 (C-6), 41.3 (C-8), 38.0 (C-7).

^{1}H NMR (500 MHz, D_2O):

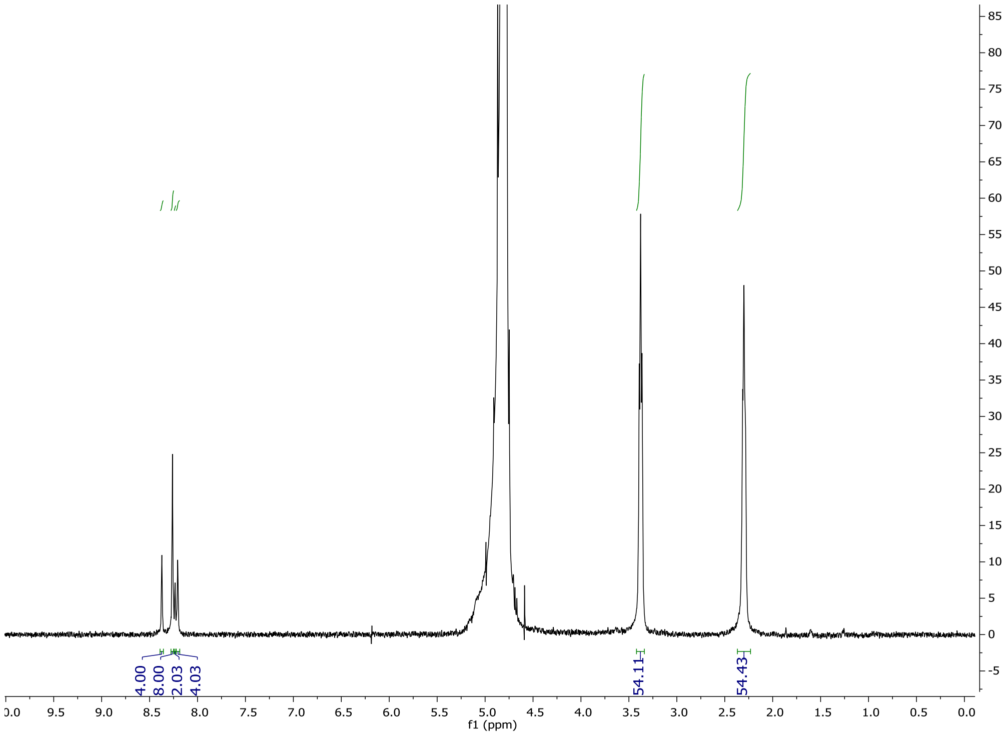

Methyl 5-acetamido-3,5-dideoxy-D-glycero-α-D-galacto-2-nonulosonic acid 14

N-Acetyl neuraminic acid methyl ester J [4]

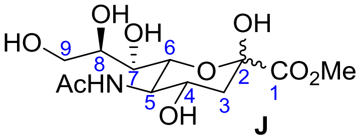

Trifluoroacetic acid (3.00 mL, 38.8 mmol) was added to a suspension of *N*-acetylneuraminic acid (10.0 g, 32.3 mmol) in anhydrous methanol (250 mL). The reaction was stirred for 72 hours, giving a clear solution. The solution was then concentrated under reduced pressure and **J** was precipitated as a white solid by addition of diethyl ether (10.1 g, 96%). **^{1}H NMR** (D_2O, 400 MHz): δ = 4.12 – 4.02 (m, 2H, H-4, H-6), 3.93 (t, *J* = 10.2 Hz, 1H, H-5), 3.85 (s, 3H, CO_2Me), 3.87 – 3.82 (m, 1H, H-9b), 3.74 (ddd, *J* = 9.0, 6.2, 2.6 Hz, 1H, H-8), 3.62 (dd, *J* = 11.8, 6.3 Hz, 1H, H-9a), 3.56 (dd, *J* = 9.2, 1.2 Hz, 1H, H-7), 2.32 (dd, *J* = 13.1, 4.9 Hz, 1H, H-3_{eq}), 2.06 (s, 3H, NH*Ac*), 1.92 (dd, *J* = 13.1, 11.6 Hz, 1H, H-3_{ax}) [lit.,[4] 3.82 (s, 3H, CO_2Me), 2.31 (dd, *J* = 5.0, 13.0 Hz, 1H, H-3e), 1.97 (dd, *J* = 11.5, 13.0 Hz, 1 H, H-3a]; **^{13}C NMR** (101 MHz, D_2O) δ = 174.8 (NH*Ac*), 171.3 (C-1), 95.3 (C-2), 70.3 (C-4), 70.0 (C-8), 68.1 (C-7), 66.6 (C-6), 63.1 (C-9), 53.4 (COO*C*H_3), 52.0 (C-5), 38.6 (C-3), 22.0 (NH*Ac*); **LRMS-ESI** $[M + Na]^+$ Calc. for $C_{12}H_{21}NO_9Na$ 346.11, Found 346.11. **Elem. Anal.** Calc'd for $C_{12}H_{21}NO_9$: C, 44.58; H, 6.55; N, 4.33; Found: C, 44.94; H, 6.64, N, 4.52.

^{1}H NMR (D_2O, 400 MHz):

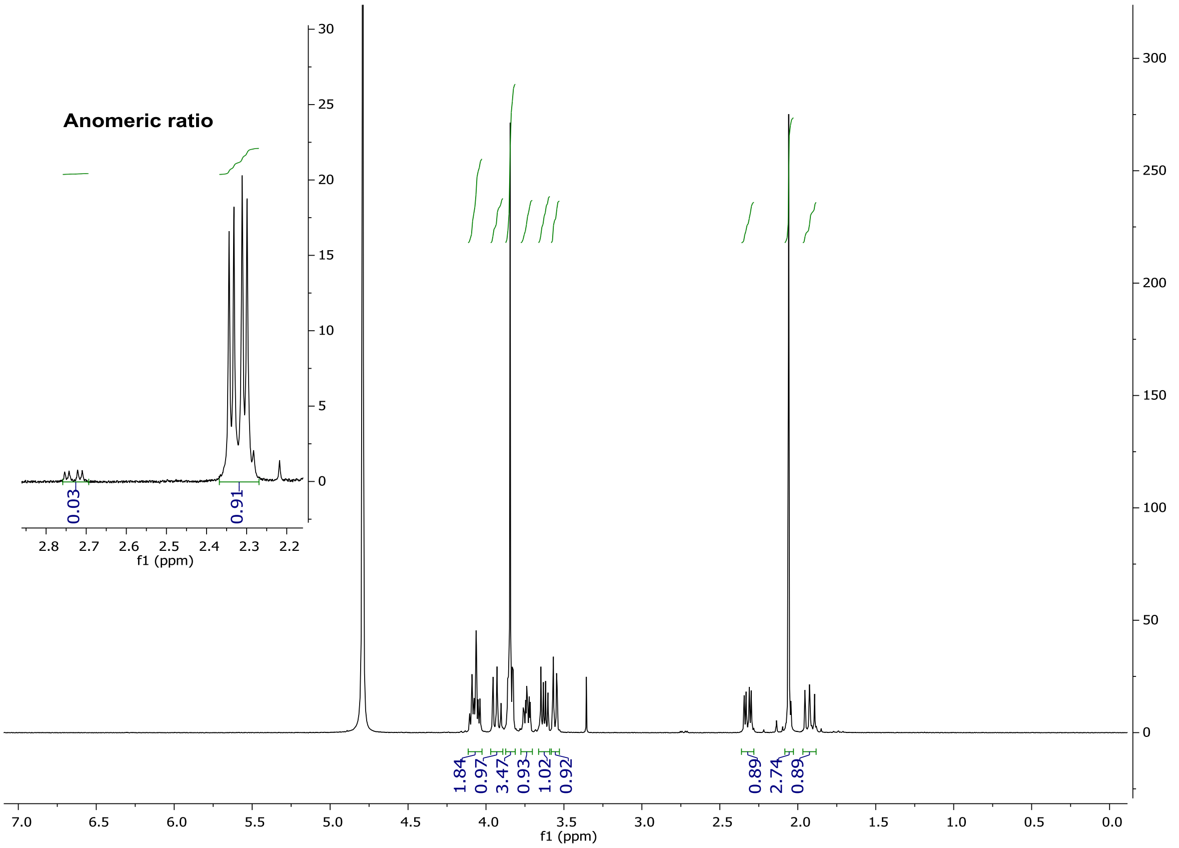

Methyl 5-acetamido-4,7,8,9-tetra-O-acetyl-2-chloro-2,3,5-trideoxy-D-glycero-D-galacto-2-nonulopyranosonate (K) [4]

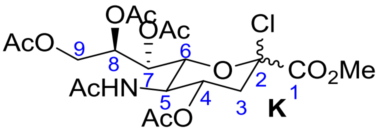

Methyl ester **J** (2.00 g, 6.19 mmol) was dissolved in acetyl chloride (80 mL). The reaction flask was flushed with nitrogen, closed and the solution stirred for 64 hours. The solution was then concentrated under reduced pressure and co-evaporated with toluene (2 x 15 mL), giving a white foam (3.16 g, quant.).* Purification by column chromatography (EtOAc) gave chloride **K** (2.65 g, 84%). ***R*$_f$ =** 0.31 (EtOAc); **^{1}H NMR** ($CDCl_3$, 400 MHz): δ = 5.47 (dd, *J* = 7.2, 2.4 Hz, 1H, H-7), 5.40 (td, *J* = 10.9, 4.8 Hz, 1H, H-4), 5.30 (d, *J* = 10.2 Hz, 1H, NH), 5.18 (ddd, *J* = 7.2, 5.7, 2.7 Hz, 1H, H-8), 4.42 (dd, *J* = 12.6, 2.7 Hz, 1H, H-9b), 4.35 (dd, *J* = 10.8, 2.4 Hz, 1H, H-5), 4.21 (dd, *J* = 10.3 Hz, 1H, H-6), 4.06 (dd, *J* = 12.5, 5.7 Hz, 1H, H-9a), 3.88 (s, 3H, CO_2Me), 2.79 (dd, *J* = 13.9, 4.8 Hz, 1H, H-3_{eq}), 2.28 (dd, *J* = 13.9, 11.2 Hz, 1H, H-3_{ax}), 2.12, 2.08, 2.06, 2.05 (4s, 12H, OAc), 1.91 (s, 3H, NH*Ac*) [lit.[4] ($CDCl_3$, 390 MHz): δ = 3.91 (s, 3H, CO_2Me), 2.78 (dd, *J* = 5.0, 12.0 Hz, 1H, H-3e), 1.92-2.10 (s, 15H NHAc, OAc)]; **LRMS-ESI** $[M + Na]^+$ Calc. for $C_{20}ClH_{28}NO_{12}$ 532.12, Found 532.13.

^{1}H NMR ($CDCl_3$, 400 MHz):

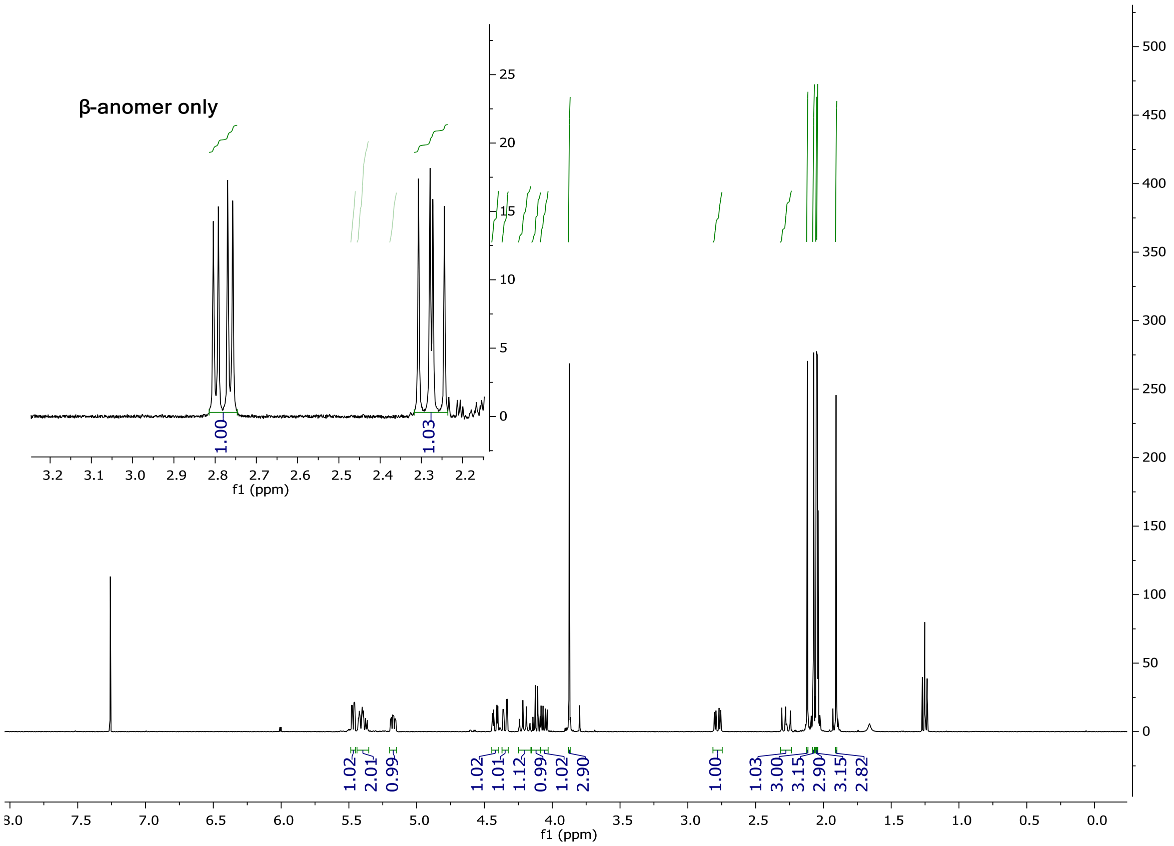

* This material could be used for the next step without further purification.

Methyl (methyl 5-acetamido-3,5-dideoxy-α-D-glycero-D-galacto-2-nonulopyranosid)onate (L) [4]

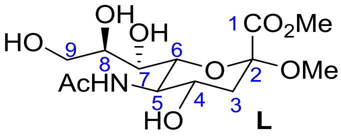

Sodium methoxide (49 mL, 0.20 M) was added to a stirring solution of chloro acetyl methyl ester **K** (2.57 g, 4.90 mmol) in anhydrous methanol (50 mL) under N_2 atmosphere. The reaction was left for 70 min before acidification (pH < 7) by addition of Amberlyst-15 H+ form resin. The mixture was then filtered and the solvent removed *in vacuo*. The residue was dissolved in MeOH and EtOAc added to precipitate the alkene side-product, which was removed by Buchner filtration. The filtrate was then evaporated to dryness and the residue purified by column chromatography (EtOAc/MeOH, 8:2) to yield a white foam. This was then crystallised (MeOH/EtOAc) to give the α-glycoside **L** as colourless cubic crystals (535 mg, 32%). **^{1}H NMR** (D_2O, 500 MHz): δ = 3.93 – 3.83 (m, 7H; CO_2Me, H-5, H-6, H-8, H-9a), 3.78 (dddd, *J* = 11.9, 8.6, 4.6, 1.2 Hz, 1H; H-4), 3.67 (dd, *J* = 6.3, 6.3 Hz, 1H; H-9a), 3.58 (dd, *J* = 9.0, 1.1 Hz, 1H; H-7), 3.40 (s, 3H; OMe), 2.70 (dd, *J* = 12.8, 4.6 Hz, 1H; H-3_{eq}), 2.05 (s, 3H, NHAc), 1.82 (dd, *J* = 12.8, 11.8 Hz, 1H, H-3_{ax}). [lit.[4] 3.78 (s, 3H), 3.38 (s, 3H), 2.69 (dd, 1H), 2.02 (s, 3H), 1.79 (dd, 1H)]; **^{13}C NMR** (D_2O, 126 MHz): δ = 174.9 (NHAc), 169.9 (C-1), 99.1 (C-2), 72.8 (C-6), 70.5 (C-8), 68.2 (C-7), 67.1 (C-4), 63.1 (C-9), 53.3 (CO_2Me), 51.7 (C-5), 51.6 (OMe), 38.8 (C-3), 22.0 (NHAc). **Elem. Anal.** Calc'd for $C_{13}H_{23}NO_9$: C, 46.29; H, 6.87; N, 4.15; Found: C, 47.05; H, 7.00, N, 4.18; **LRMS-ESI** $[M + Na]^+$ Calc. for $C_{13}H_{23}NNaO_9$ 360.13, Found 360.13.

^{1}H NMR (D_2O, 500 MHz):

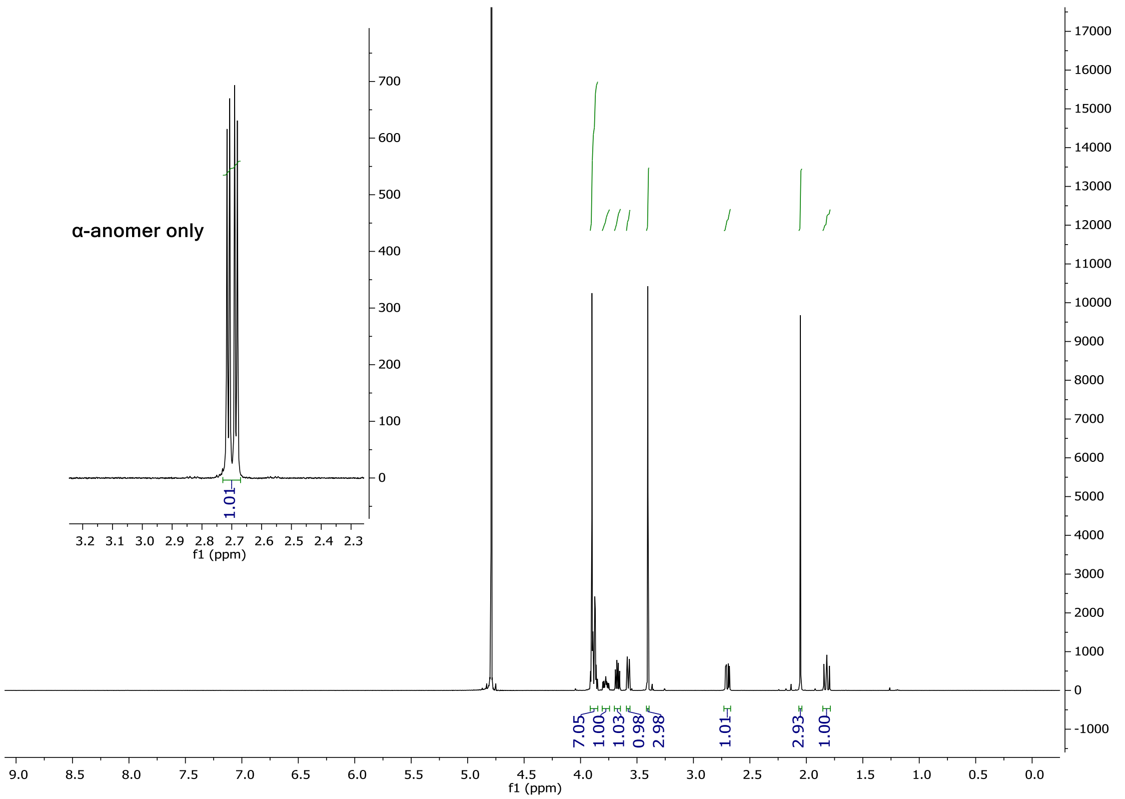

^{1}H-^{13}C HSQC NMR (D_2O, 500, 126 MHz):

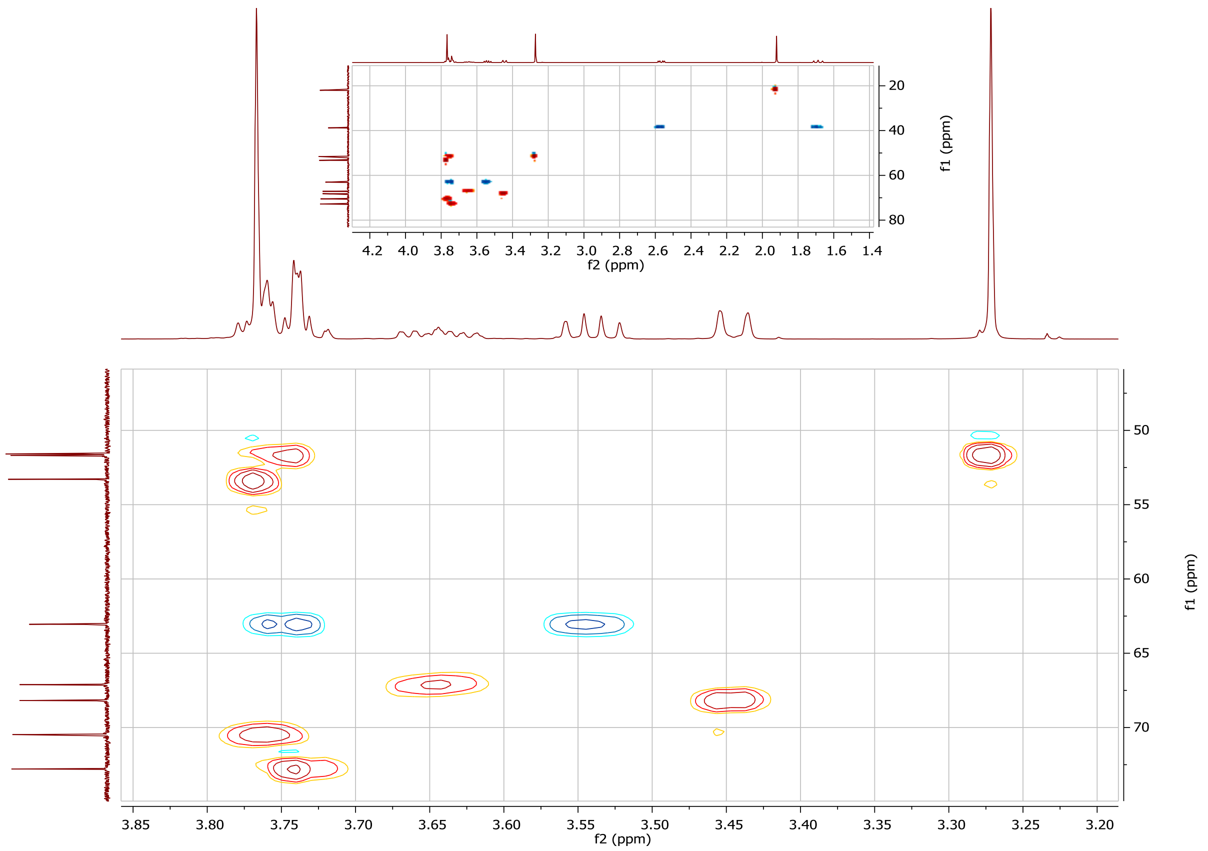

Methyl 5-acetamido -3,5-dideoxy-D-glycero-α-D-galacto-2-nonulosonic acid (14) [5]

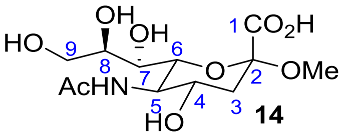

14

Methyl ester **L** (40.0 mg, 0.12 mmol) was dissolved in sodium hydroxide solution (1.20 mL, 1 M in H_2O) and stirred for 45 mins. Amberlyst-15 H^+-form ion exchange resin was then added portionwise until the solution pH < 6 (pH meter). The mixture was filtered through cotton wool and a syringe filter (0.45 µm) before freeze drying to give sialoside **14** (38.3 mg, quant., α/β > 97:3). **^{1}H NMR** (D_2O, 400 MHz): δ = 3.92 – 3.84 (m, 3H; H-8, H-9b, H-6), 3.82 (d, *J* = 8.5 Hz, 1H; H-5), 3.80 – 3.70 (m, 1H; H-4), 3.65 (dd, *J* = 12.0, 6.4 Hz, 1H; H-9a), 3.58 (d, *J* = 8.7 Hz, 1H; H-7), 3.39 (s, 3H; OMe), 2.70 (dd, *J* = 12.7, 4.6 Hz, 1H; H-3_{eq}), 2.04 (s, 3H; NHAc), 1.72 (t, *J* = 12.2 Hz, 1H; H-3_{ax}). [lit.[5] 3.34 (OMe), 2.72 (H-3_{eq}), 1.64 (H-3_{ax})]; **^{13}C NMR** (D_2O, 100 MHz): δ = 174.8 (NHAc), 173.7 (CO_2Me), 100.0 (C-2), 70.3 (C-6), 70.1 (C-4), 68.2 (C-7), 63.1 (C-9), 52.0 (C-5), 48.8 (OMe), 38.8 (C-3), 22.0 (NHAc). **LRMS-ESI** [M + Na]$^+$ Calc. for $C_{12}H_{21}NO_9Na$ 346.11, Found 346.11.

^{1}H NMR (D_2O, 400 MHz):

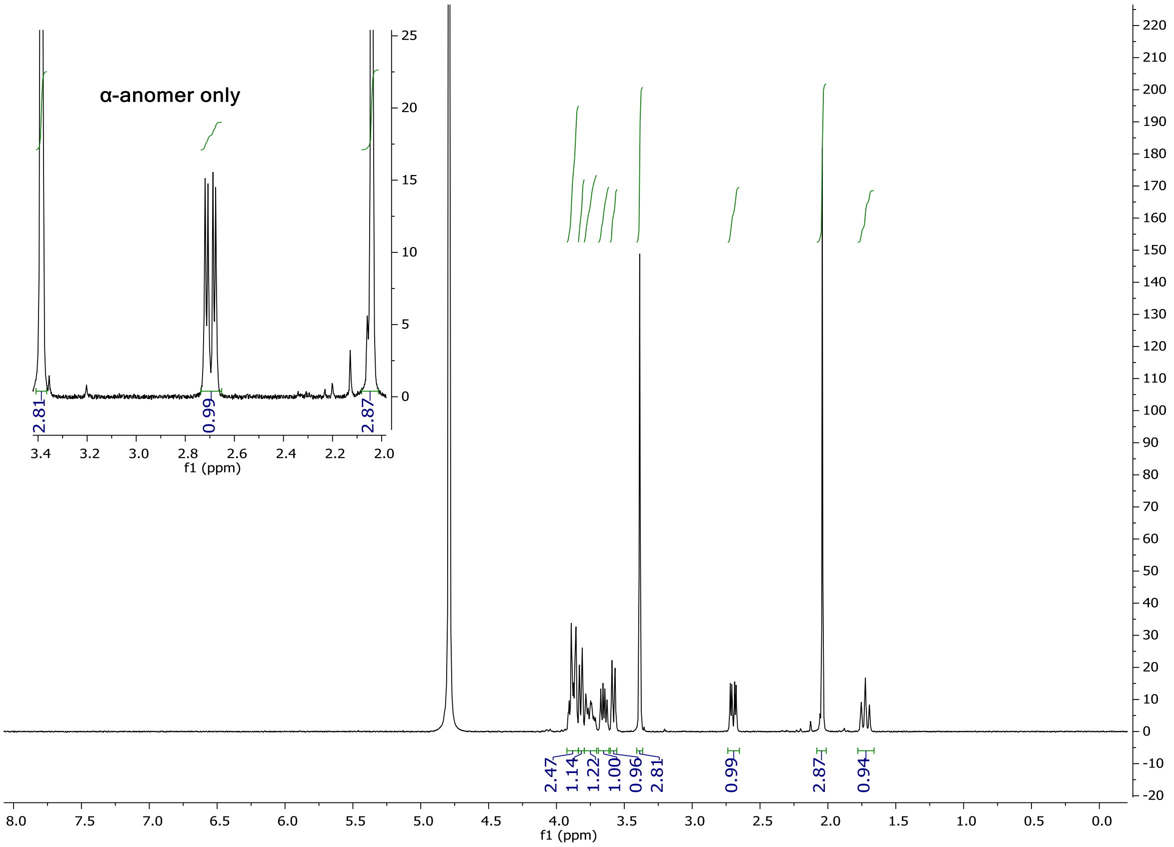

2. Spectroscopic and Binding Studies

General Methods for Binding Studies

Preparation of titration solutions. Stock solutions of carbohydrate guests were prepared (in D_2O or H_2O) at pH 7, with pH adjustment if necessary, using ion exchange resins to avoid salt formation. For example with aminosaccharide hydrochloride salts, Amberlyst A-26 (OH^- form) was used to sequester chloride and bring pH >7, before filtering and gradual addition of HCl_{aq} (0.4 M or 40 mM) to give a solution at pH = 7. Stock solutions containing reducing sugars were allowed to equilibrate before use. A receptor stock solution (pH = 7) was then employed both to prepare the titrand solution and, with a sugar stock solution, to prepare the titrant. In this way the titrant and titrand contain equal concentrations of receptor.

Fluorescence titration experiments. A PerkinElmer LS45 fluorescence spectrometer was used to measure the fluorescence emission spectra of receptor solutions (typically 0.13 - 0.50 µM) in a Hellma 111-QS quartz cuvette (3 mL, 10 mm path length). Fluorescence emission intensity was recorded in arbitrary units (a.u.) and emission wavelength in nanometres (nm). The thermostat feature was used to maintain a temperature of 25 °C. Spectra were recorded for the receptor solution with increasing guest concentration, while volume and host concentration remained constant. The procedure for each addition was thus: removal of a volume from the cuvette by Gilson pipette; replacement with the same volume of guest titrant solution (containing host); stirring of cuvette with micro stirrer bar for at least 5 min, with monitoring of emission intensity over time; solution allowed to settle for 1 min; spectrum collected twice ensuring intensity is stable. After the titration, the pH of the solution in the cuvette was measured, confirming that no change had occurred.

^{1}H NMR titration experiments. A Varian VNMRS500b (500 MHz) or a Varian VNMRS600 Cryo (600 MHz) spectrometer were used to measure the ^{1}H NMR spectra of receptor solutions (typically 0.15 - 0.25 mM) in D_2O at 298 K, with varying concentrations of guest. A solution of receptor was placed in an NMR tube and a spectrum was recorded, aliquots of guest titrant were then added and the tube shaken, with a spectrum recorded after each addition. After the titration, the pH of the solution within the NMR tube was measured, ensuring no change had occurred.

Calculation of association constants (K_1, K_2). The data resulting from each titration experiment was entered into an Excel spreadsheet, where the change in fluorescence emission intensity (or receptor chemical shift) was plotted against guest concentration, to give a binding curve. The data was then fit to a 1:2 (Host:Guest) binding model using a non-linear least squares regression, based on the variation of the sequential association constants (K_1, K_2) and limiting values (Em_{HG}, Em_{HG2} or δ_{HG}, δ_{HG2}) to maximise the goodness of fit (measured by correlation coefficient, r).[6,7] To simplify the data fitting procedure (for 1:2 stoichiometries), the free guest concentration ([G]) was assumed to be equal to total guest concentration ($[G]_t$). This

approximation is reasonable in cases where $[H]_t$ is substoichiometric relative to $[G]_t$ during the entire titration, such as the experiments reported herein. Limiting values were constrained within reasonable boundaries, for example ensuring that limiting fluorescence emission values are greater than zero. A plot with the calculated and observed data overlaid is given for each titration experiment, and in addition the species distribution predicted by the fitting process is given.

For some titrations (where indicated), HypNMR 2008 (version 4.0.71, Protonic Software) was used to fit the data to a binding model. This was necessary when following the change in the titrant (as opposed to titrand) chemical shift. In such cases the limiting chemical shifts are found at titrant concentrations before the first data point. This situation was incompatible with theExcel spreadsheet, but could be handled by the HypNMR programme. HypNMR is also capable of fitting several NMR peaks simultaneously to a binding model, as in the study of **16** + **14** (Figure S56 - Figure S59).

2D NOESY experiments. For receptor-substrate pairings where binding was relatively strong a Varian VNMRS600 Cryo (600 MHz) spectrometer was used to measure 2D NOESY spectra (36 - 128 scans, mixing time = 300 ms).

1D NOESY experiments. For cases of weaker binding, where high concentrations of carbohydrate were needed to promote complex formation, a selective 1D NOESY spectrum was obtained. The region containing the receptor aromatic signals was selectively irradiated, to give a spectrum which shows any NOE correlations to these protons. A Bruker Advance III HD 500 Cryo (500 MHz) spectrometer was employed, each spectrum involving 1500 scans with mixing time = 300 ms.

Job Plot experiments. Equimolar stock solutions of receptor and guest were prepared (0.50 or 1.00 mM) in D_2O at pH 7. Seven samples (150 µL) were then prepared in 3 mm NMR tubes, using varying proportions of the stock solutions, and ^1H NMR spectra were recorded. For each sample, the change in receptor chemical shift ($\Delta\delta$) relative to the sample containing only receptor was measured. This value scaled by the mol fraction of host (χ_H = [host]/([host]+[guest])) was used to give an indication of the proportion of bound host, and plot against mol fraction of guest (χ_G = [guest]/([host]+[guest])). The maximum of this plot indicates the mol fraction of guest in the host-guest complex, this can then be extrapolated to the binding stoichiometry.

Anionic Receptor 9 – Spectroscopic Studies.

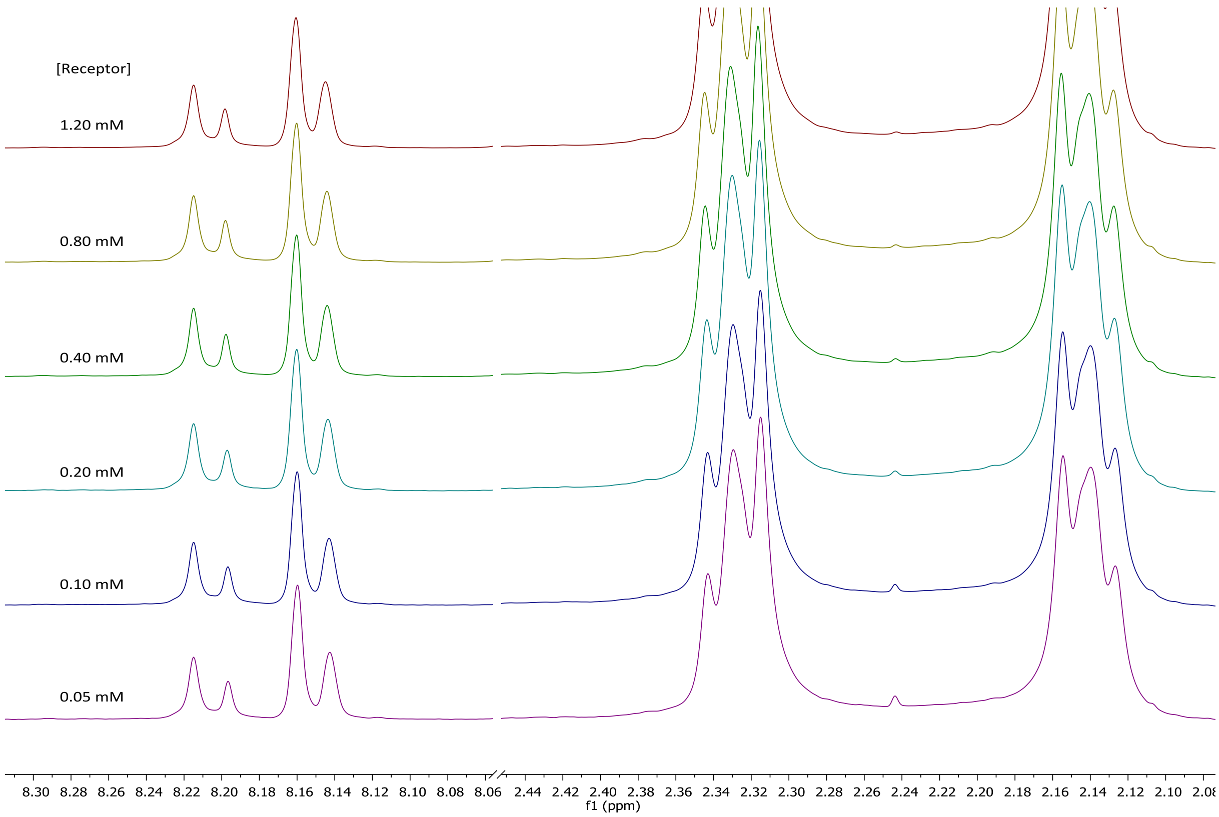

Figure S1. ^{1}H NMR dilution study of receptor **9** in D_2O at 298 K. Concentrations range from 1.20 mM to 0.05 mM.

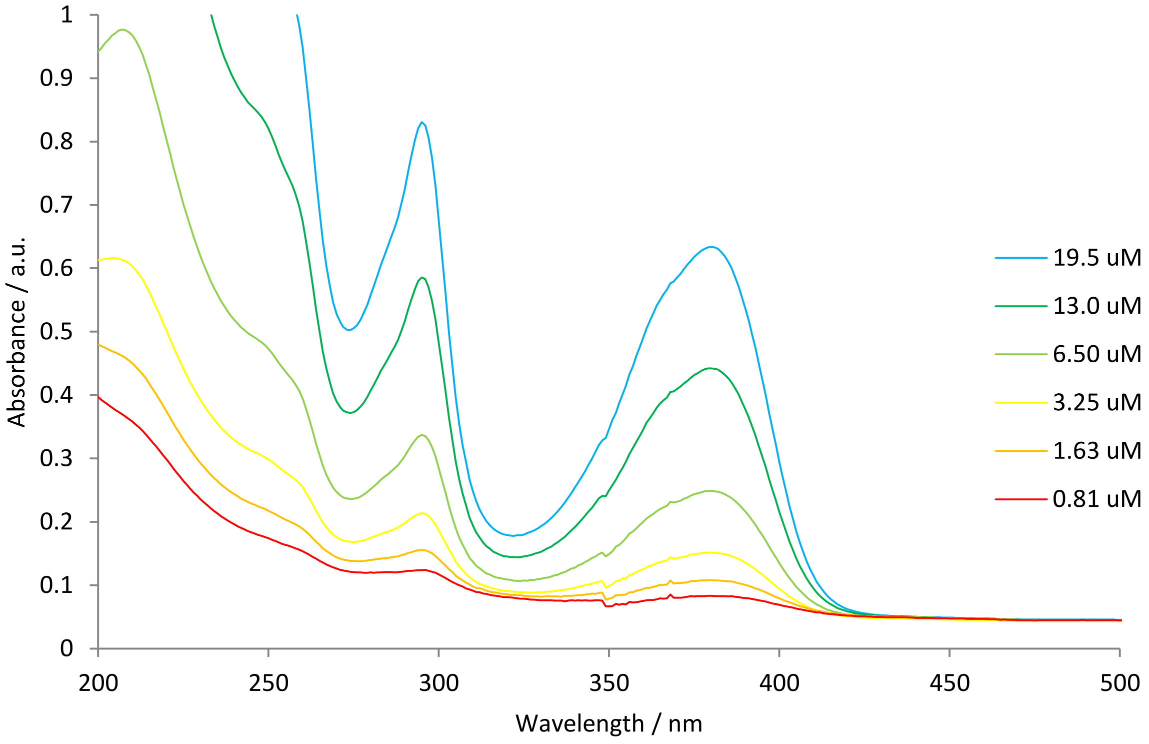

Figure S2. UV/Vis Absorbance spectrum for receptor **9** at a range of concentrations (19.5 – 0.81 μM) in H_2O at 298 K.

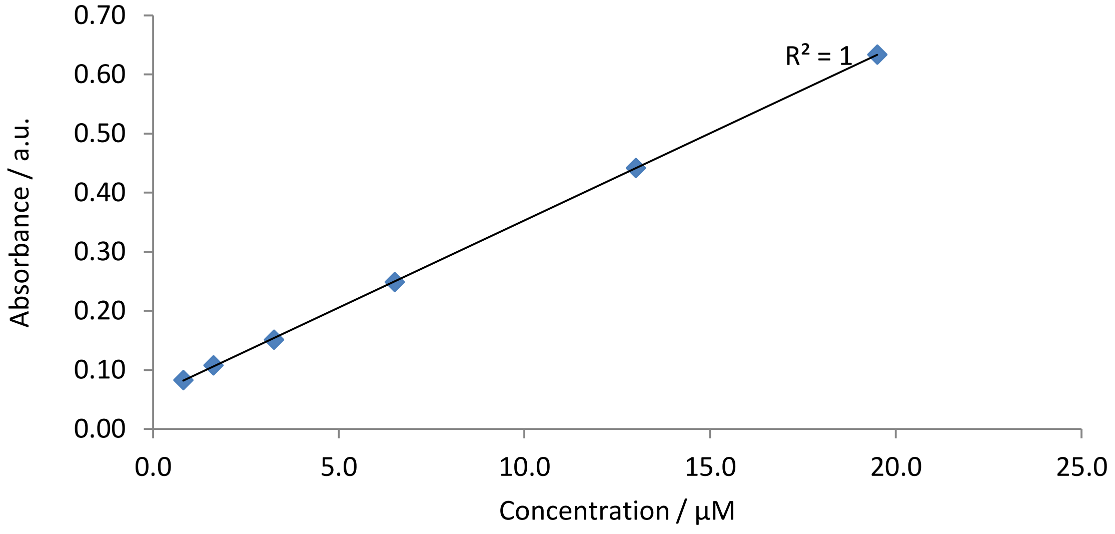

Figure 3. UV-Vis absorbance of receptor **9** at 380 nm in H_2O plotted over a range of concentrations (19.5 to 0.81 μM) at 298 K. A linear relationship between concentration and absorbance is observed.

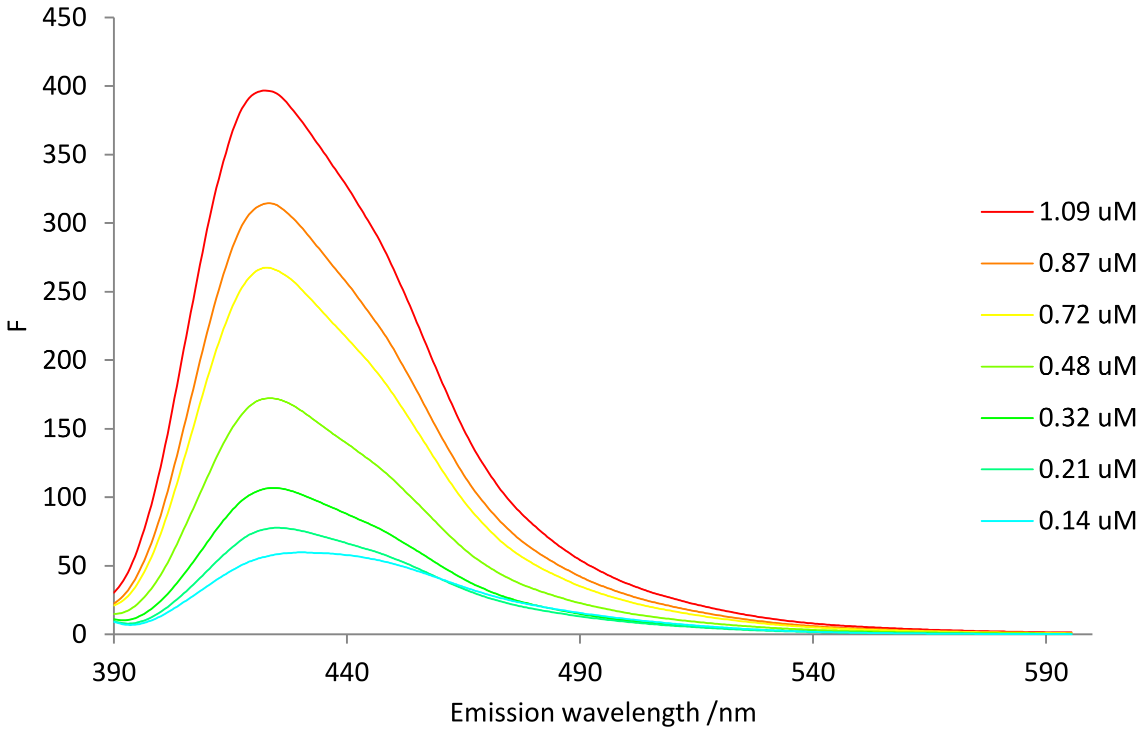

Figure S4. Fluorescence emission spectra for receptor **9** in H_2O at 298 K, over a range of concentrations from 0.82 µM to 12.3 nM. Excitation wavelength 380 nm.

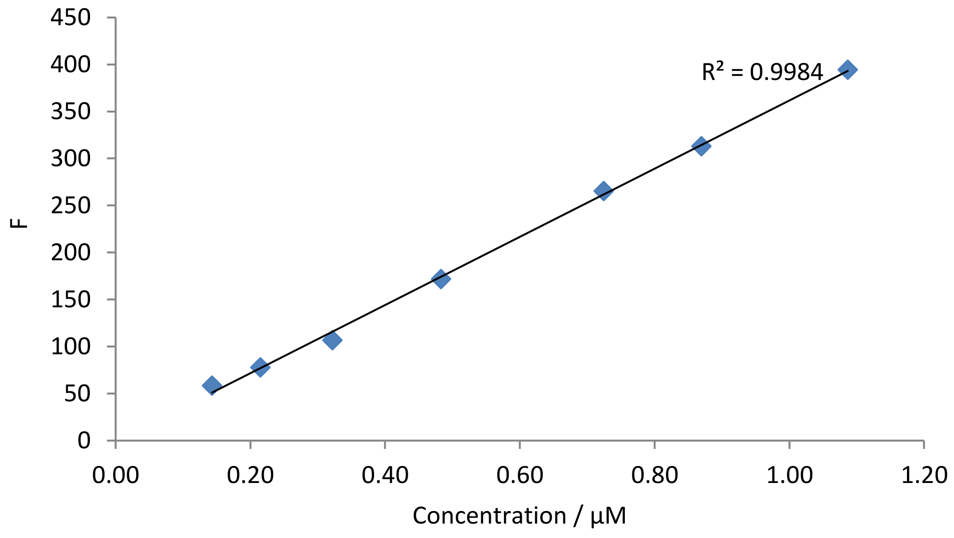

Figure S5. A plot of receptor **9** concentration against fluorescence emission intensity (425 nm) in H_2O at 298 K, excitation wavelength 380 nm. A linear relationship between emission intensity and concentration is observed.

Anionic Receptor 9 – Binding Studies.

Mannosamine 10

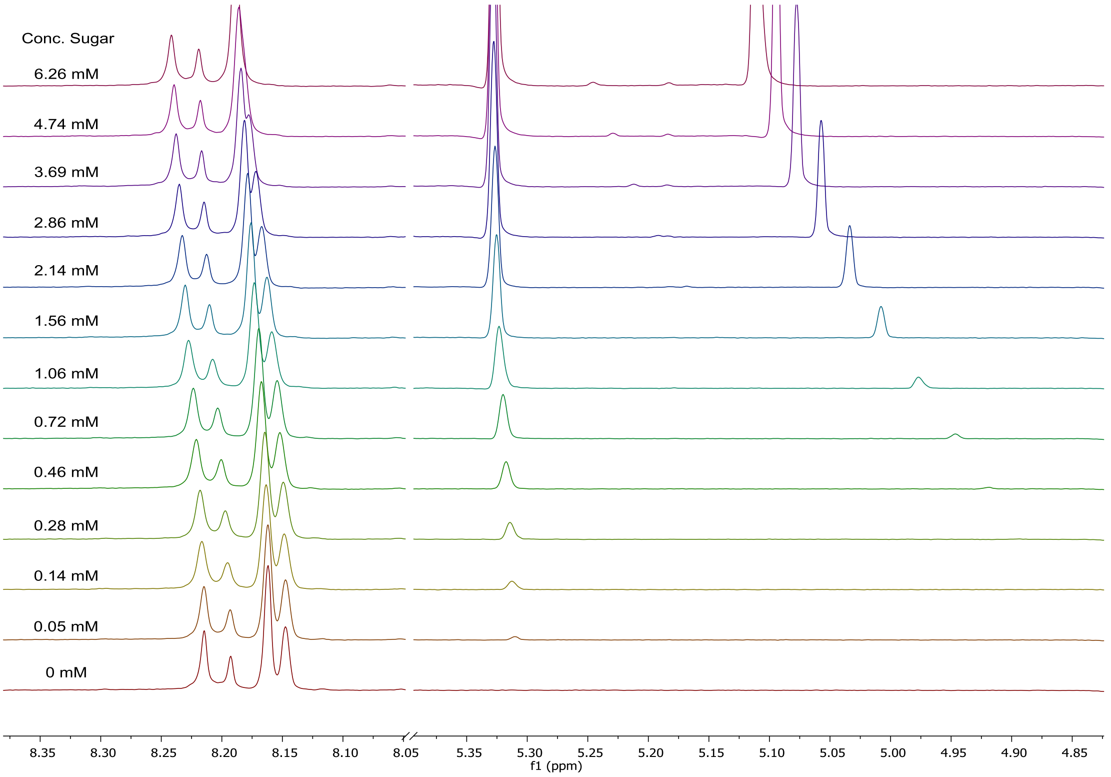

Figure S6. Partial spectra from a ^{1}H NMR titration of receptor **9** (0.20 mM) with D-mannosamine **10** (21.0 mM) in D_2O at pH 7.

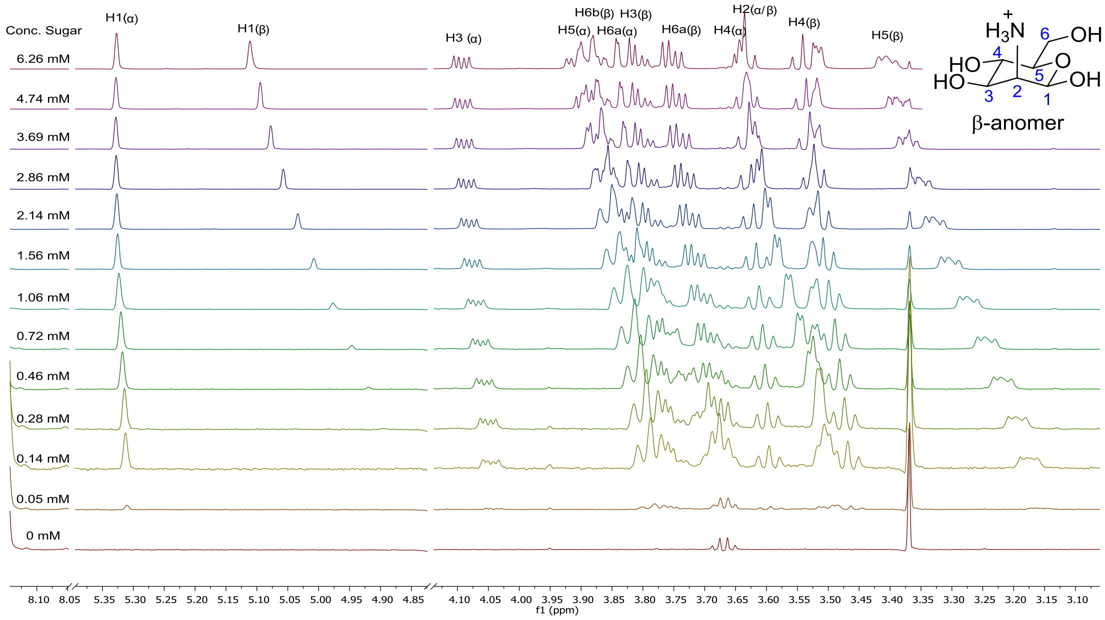

Figure S7. Partial spectra from a ^{1}H NMR titration of receptor **9** (0.20 mM) with D-mannosamine **10** (21.0 mM) in D_2O at pH 7 (as above, but rescaled to standardise carbohydrate signals). H1(β) is suppressed as a result of its proximity to the HDO peak. Carbohydrate α/β ratio = 1:1.85.

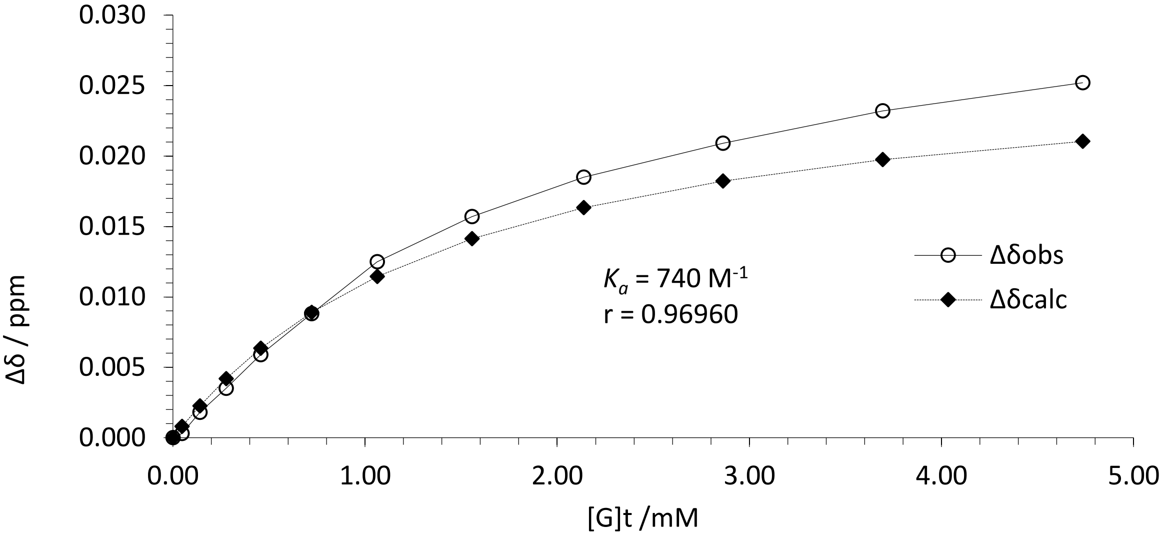

Figure S8. Data analysis for ^{1}H NMR titration of receptor **9** with D-mannosamine **10** (see above). Plot of observed and predicted changes in chemical shift (ppm) against guest concentration (mM), in accordance with a 1:1 binding model with K_a = 740 M^{-1}. Initial chemical shift is δH = 8.2150 ppm. Limiting chemical shift is δHG = 8.2420 ppm. r = 0.96960. The poor fit supports our hypothesis of receptor:substrate 1:1 + 1:2 stoichiometry.

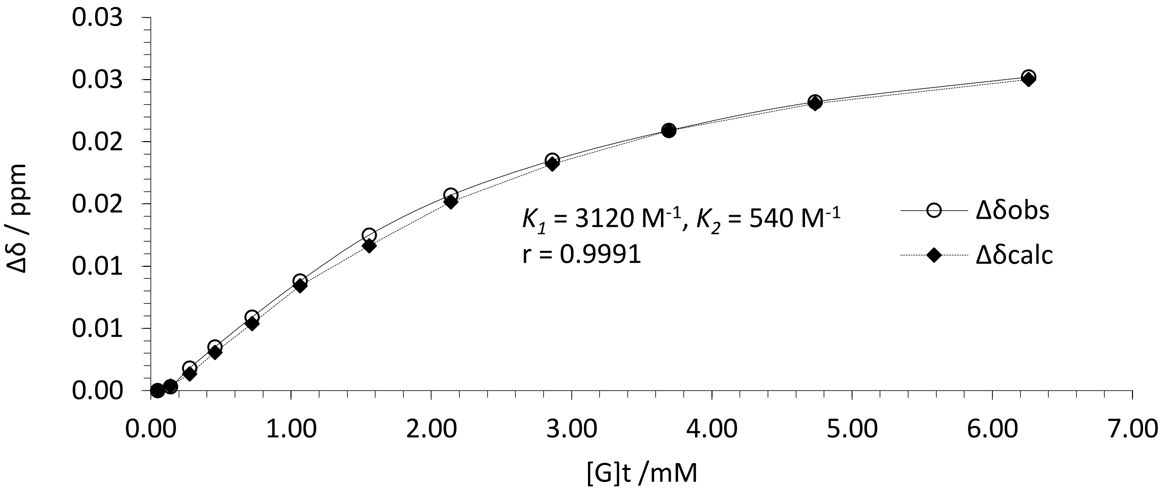

Figure S9. Data analysis for ^{1}H NMR titration of receptor **9** with D-mannosamine **10** (see above). Plot of observed and predicted changes in chemical shift (ppm) against guest concentration (mM), in accordance with a receptor:substrate 1:2 binding model with K_1 = 3120 M^{-1} and K_2 = 540 M^{-1}. Initial chemical shift is δH = 8.2150 ppm. Limiting chemical shifts are δHG = 8.2164 and δHG_2 = 8.2492 ppm. r = 0.9991.

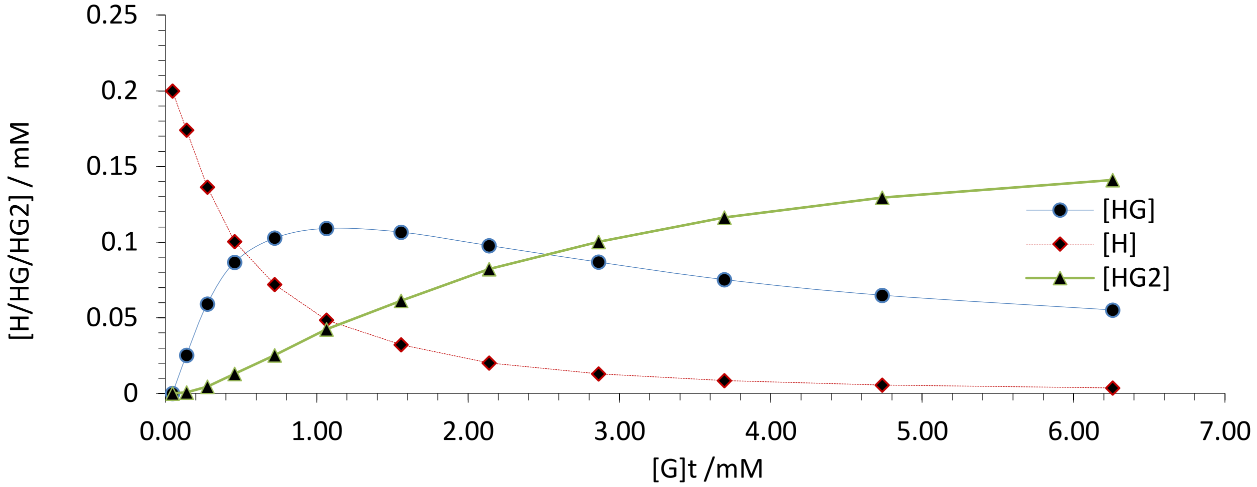

Figure S10. Species distribution resulting from analysis of the NMR binding study of receptor **9** with D-mannosamine **10** (see above).

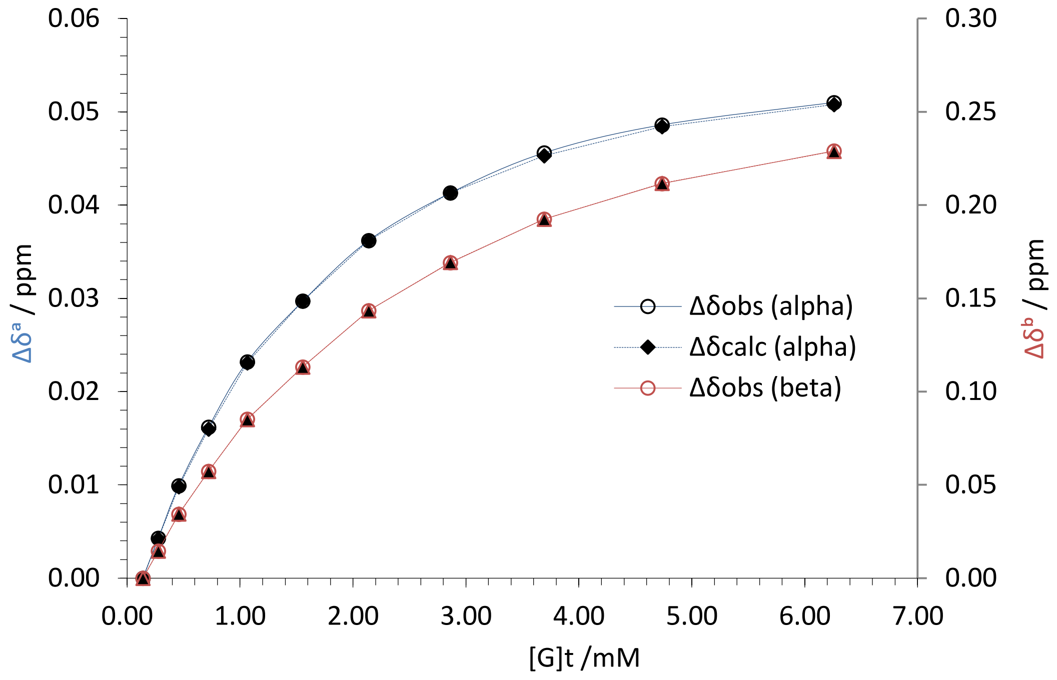

Figure S11. Data analysis for ^{1}H NMR titration of receptor **9** with D-mannosamine **10** (see above). In this case the D-mannosamine **10** signals are followed and the movements analysed by HypNMR. Mannosamine α:β ratio = 1.00:1.85 by ^{1}H NMR integration. Observed and predicted changes in chemical shift (ppm) are plotted against total guest concentration (mM) for each anomer (blue = alpha, red = beta). The movements were fitted simultaneously to two receptor:substrate 1:2 binding models, giving K_1 = 3142 M^{-1} and K_2 = 171 M^{-1} for the alpha anomer, and K_1 = 3165 M^{-1} and K_2 = 1184 M^{-1} for the beta anomer. Calculated chemical shifts of free guests are δG^a = 4.1009 and δG^b = 3.4763 ppm. Chemical shifts of first observed point ([G] = 0.0465 mM) are δG^a = 4.0405 and δG^b = 3.1590 ppm. Limiting chemical shifts are δHG^a = 3.9389 and $\delta HG^a{}_2$ = 3.1451 ppm, δHG^b = 2.6387 and $\delta HG^b{}_2$ = 2.5408 ppm. r^a = 0.999981, r^b = 0.999999. The possible mixed anomer complex HG^aG^b was not included in the fitting. Due to the complexity of the binding model the results are considered tentative, but the values for K_1 are consistent with other measurements.

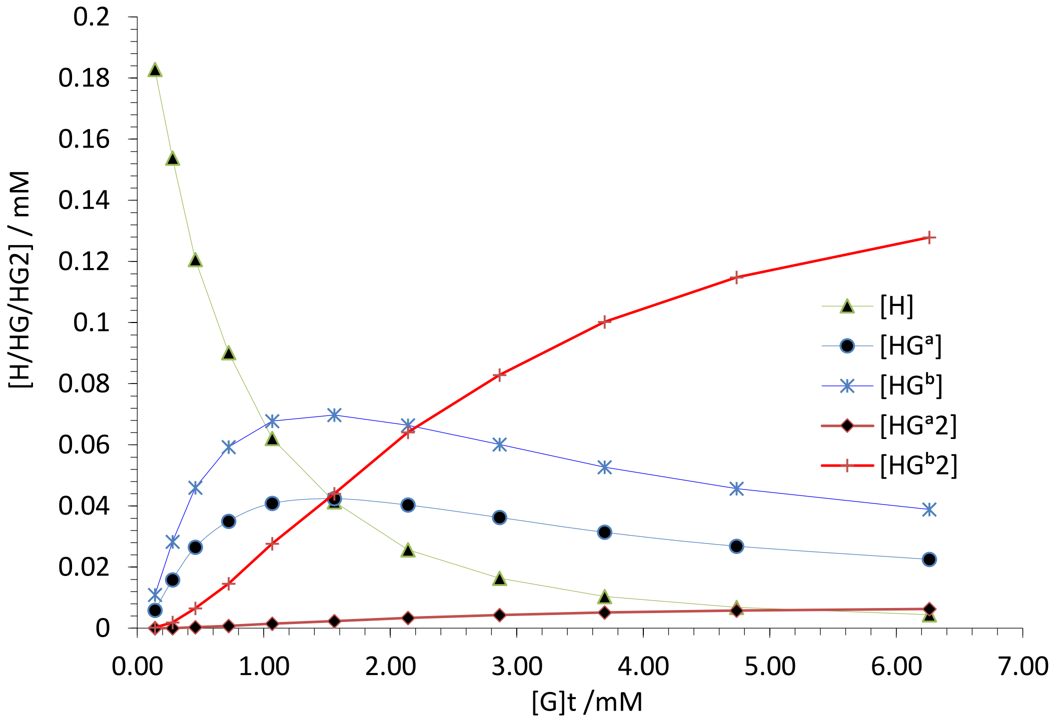

Figure S12. Species distribution related to Figure S11, resulting from the 1:2 fits of the NMR binding study of receptor **9** with D-mannosamine **10**.

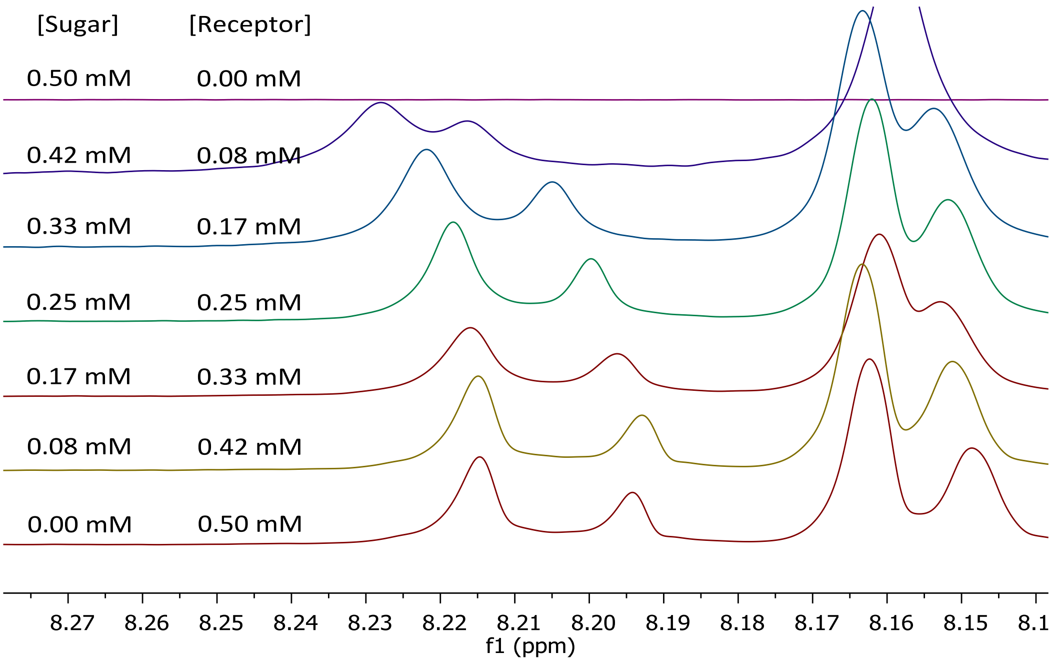

Figure S13. Partial ^{1}H NMR spectra of receptor **9** with D-mannosamine **10** in D_2O at 298 K and pH 7, at various mol fractions as required for a Job Plot.

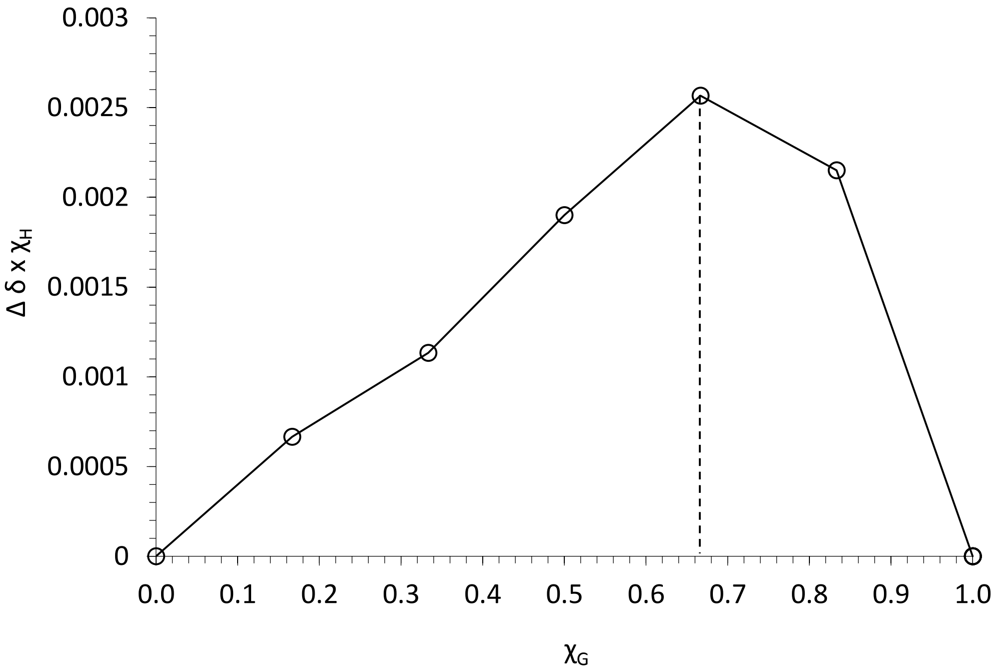

Figure S14. Job Plot for receptor **9** + D-mannosamine **10**, based on above data, employing the signal at δ_H = 8.2144 ppm in unbound **9**. X_G = mol fraction of guest ([guest]/[host]+[guest]). Maximum at X_G = 0.66 indicates a 1:2 (H:G) binding stoichiometry. Total [receptor] + [sugar] = 0.50 mM.

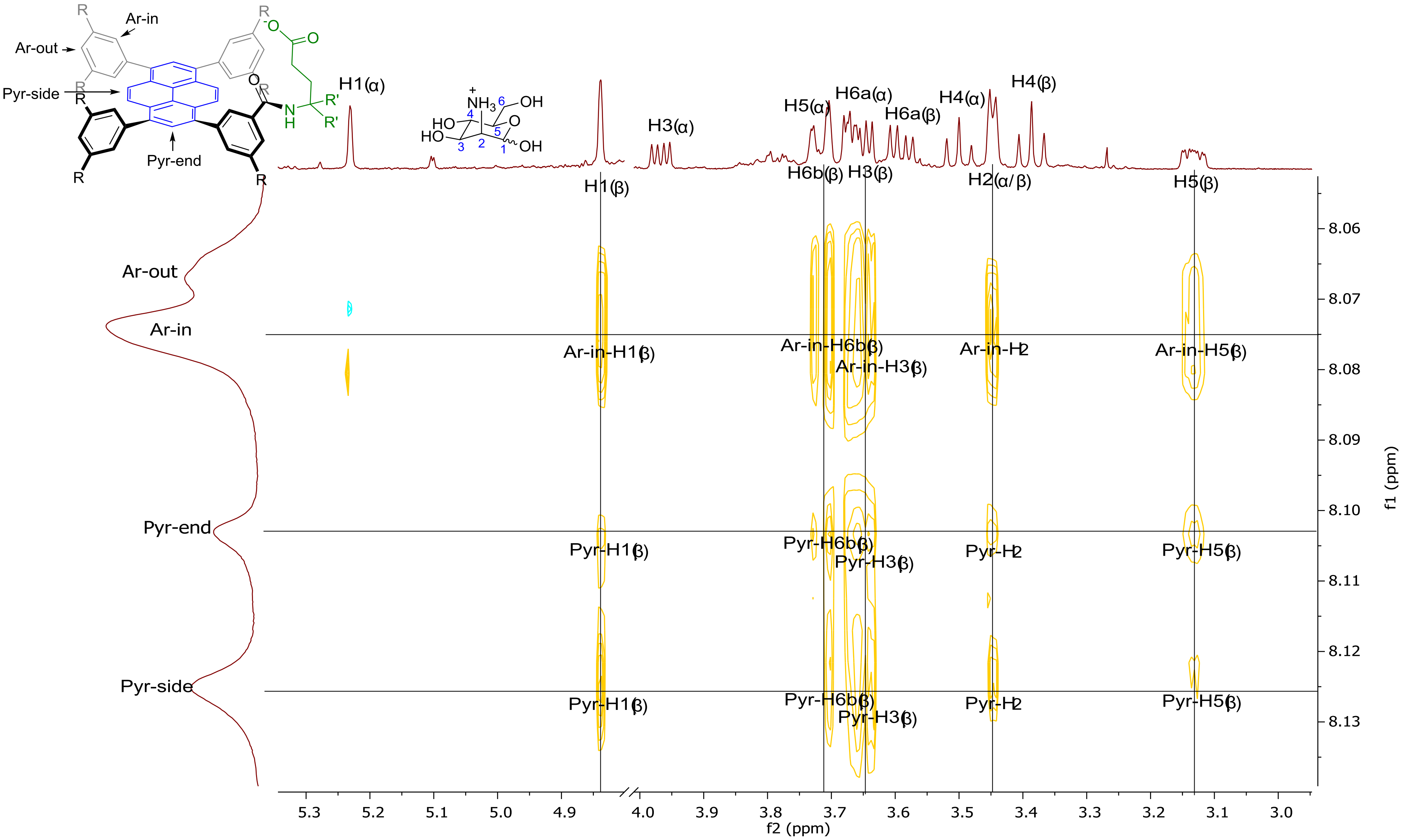

Figure S15. 2D-^{1}H-NOESY spectrum of receptor **9** (0.50 mM) with D-mannosamine **10** (6.74 mM) in D_2O at pH 7. Mixing time of 300 ms. Carbohydrate α/β ratio = 1:1.85. Receptor aromatic peaks are shown in the vertical and carbohydrate peaks are shown in the horizontal. Carbohydrate peaks for both anomers are labelled, where H1(α) refers to the anomeric proton (H1) and the α-anomer. Dashed lines provide a guide to the apparent location of the cross peaks. Connections from receptor to the mannosamine β anomer are especially clear, and are consistent with the structure in Figure 2. In particular, well-resolved cross-peaks are observed involving H1(β) and H5(β), both on the α face of the substrate, but not H4(β) on the β face.

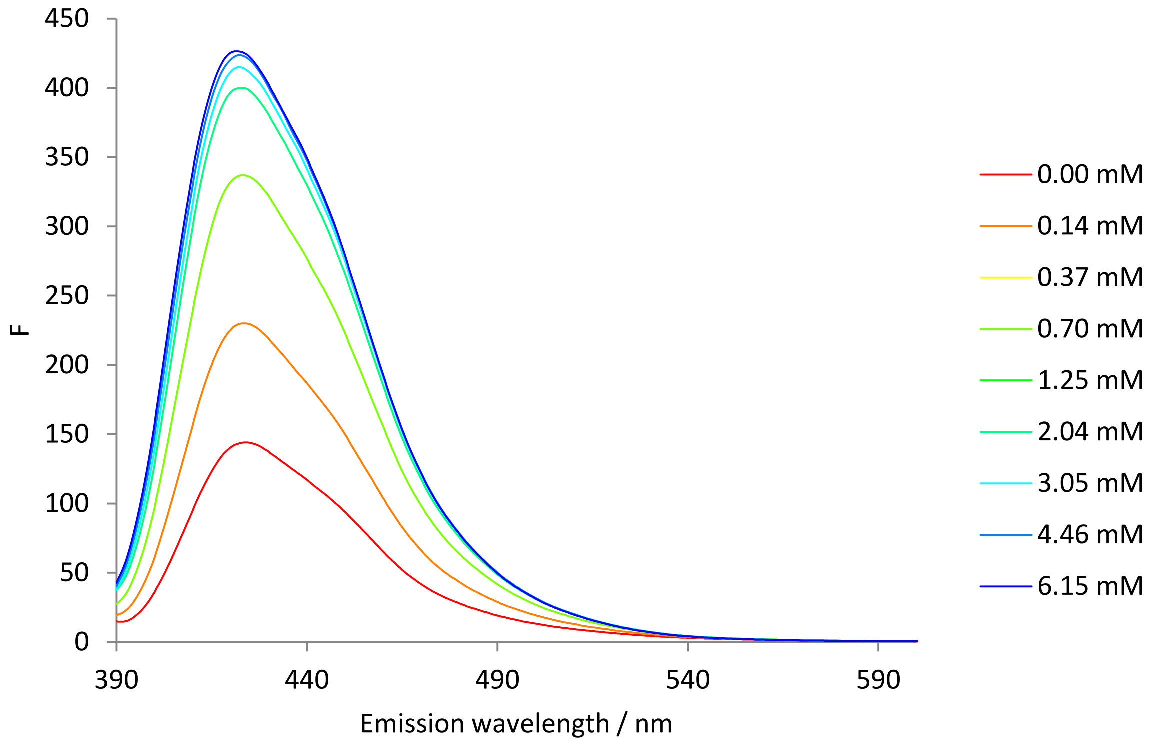

Figure S16. Fluorescence emission titration of receptor **9** (0.33 μM) with D-mannosamine **10** (17.1 mM) at pH 7 in H_2O at 298 K, excitation wavelength 380 nm.

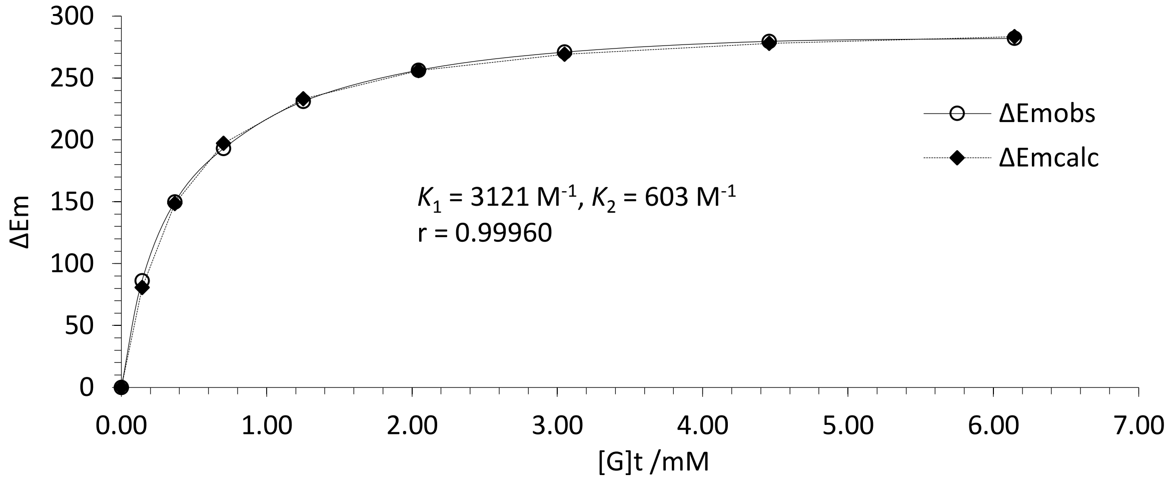

Figure S17. Data analysis for fluorescence binding study of receptor **9** titrated with D-mannosamine **10** (see above). Plot of observed and predicted emission intensity (423 nm) against guest concentration (mM) in accordance with a receptor:substrate 1:2 binding model with K_1 = 3121 M^{-1} and K_2 = 603 M^{-1}. Limiting fluorescence at 423 nm is Em_{HG} = 388 and Em_{HG2} = 442. r = 0.99960.

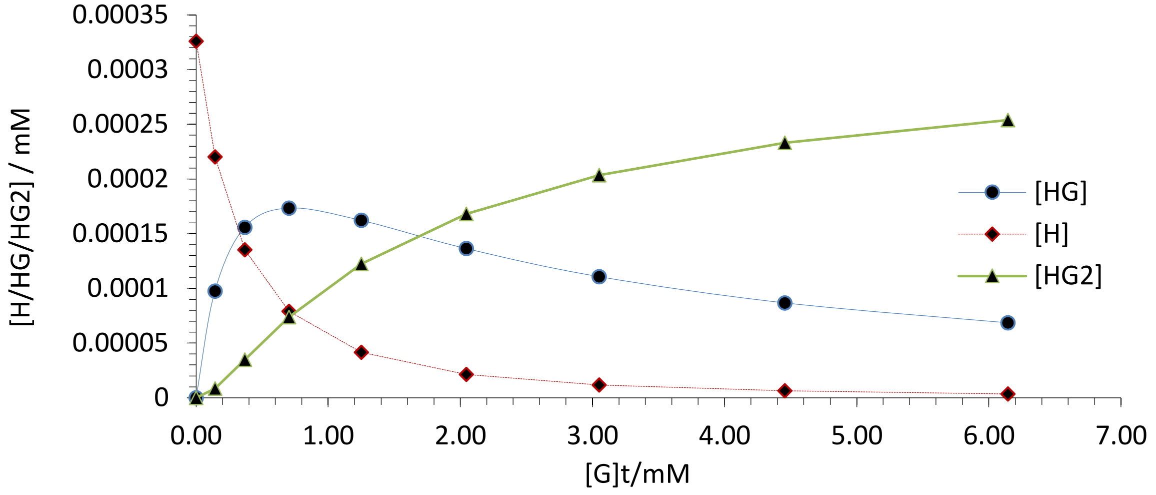

Figure S18. Species distribution resulting from analysis of the fluorescence binding study of receptor **9** titrated with D-mannosamine **10** (see above).

Galactosamine 11

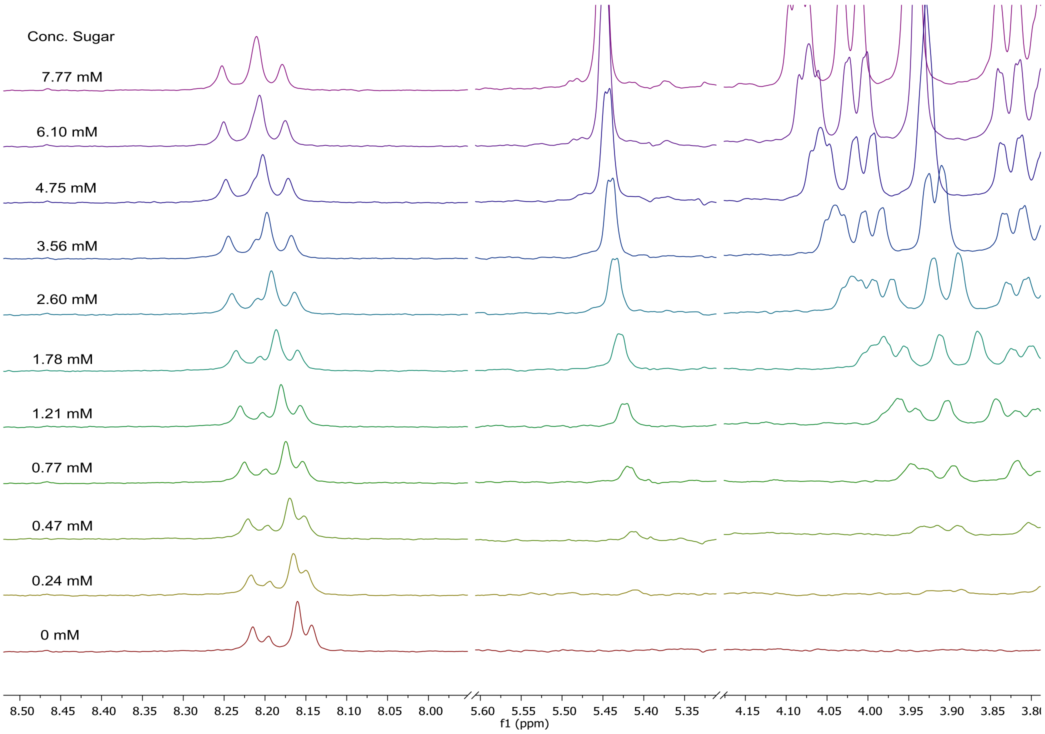

Figure S19. Partial spectra from a ^{1}H NMR titration of receptor **9** (0.20 mM) with D-galactosamine **11** (31.5 mM) in D_2O at pH 7.

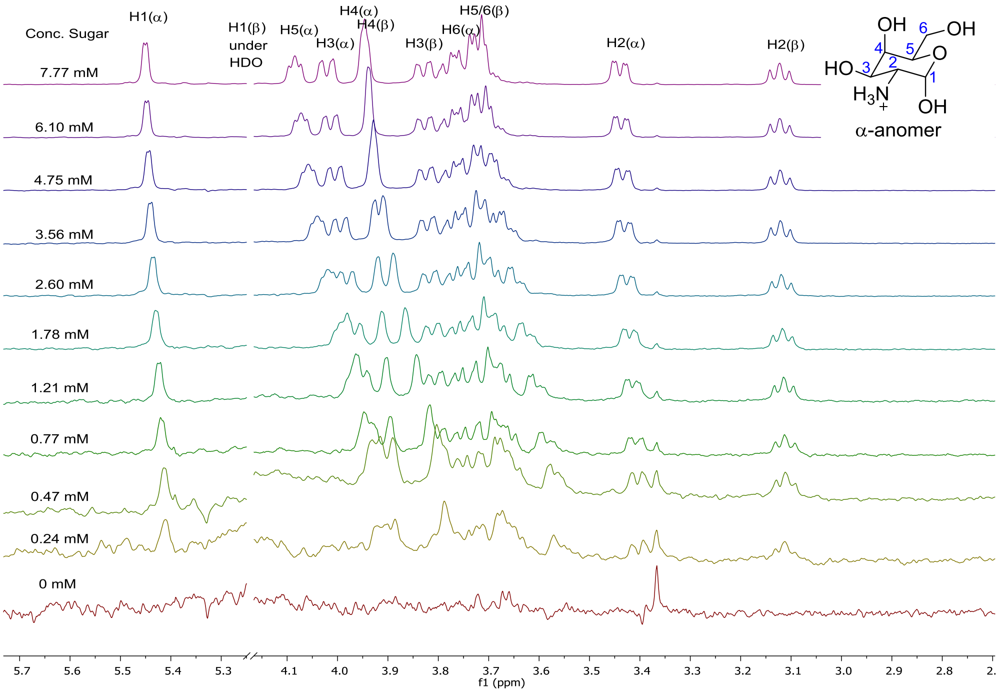

Figure S20. Partial spectra from a ^{1}H NMR titration of receptor **9** (0.20 mM) with D-galactosamine **11** (31.5 mM) in D_2O at pH 7 (as above, but rescaled to standardise carbohydrate signals). H1(β) peak is hidden by HDO signal. Carbohydrate α/β ratio = 1:0.73.

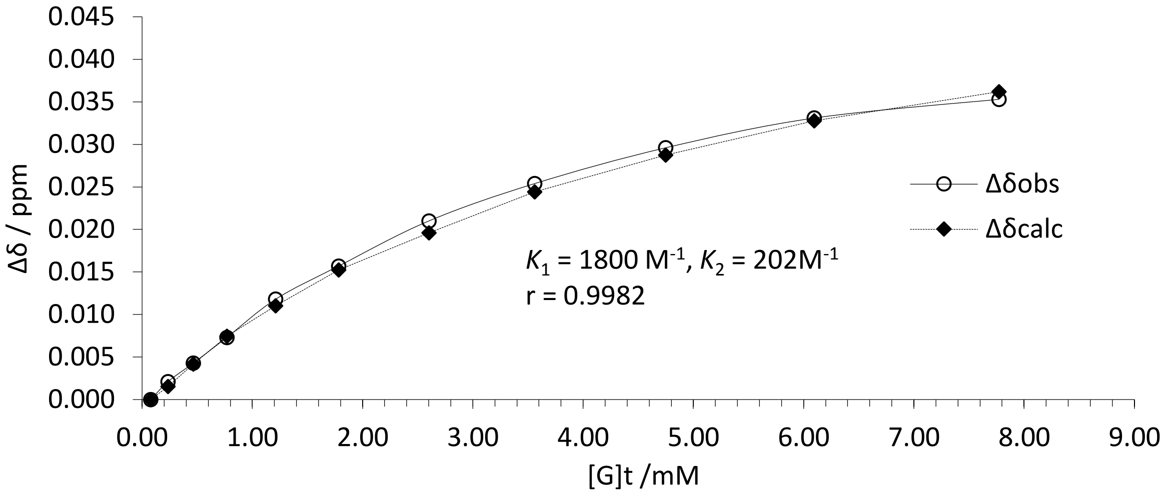

Figure S21. Data analysis for ^{1}H NMR titration of receptor **9** with D-galactosamine **11** (see above). Plot of observed and predicted changes in chemical shift (ppm) against guest concentration (mM), in accordance with a receptor:substrate 1:2 binding model with K_1 = 1800 $M^{-1}$and K_2 = 202 M^{-1}. Initial chemical shift is δH = 8.2149 ppm. Limiting chemical shifts are δHG = 8.2264 and δHG_2 = 8.2738 ppm. r = 0.9982.

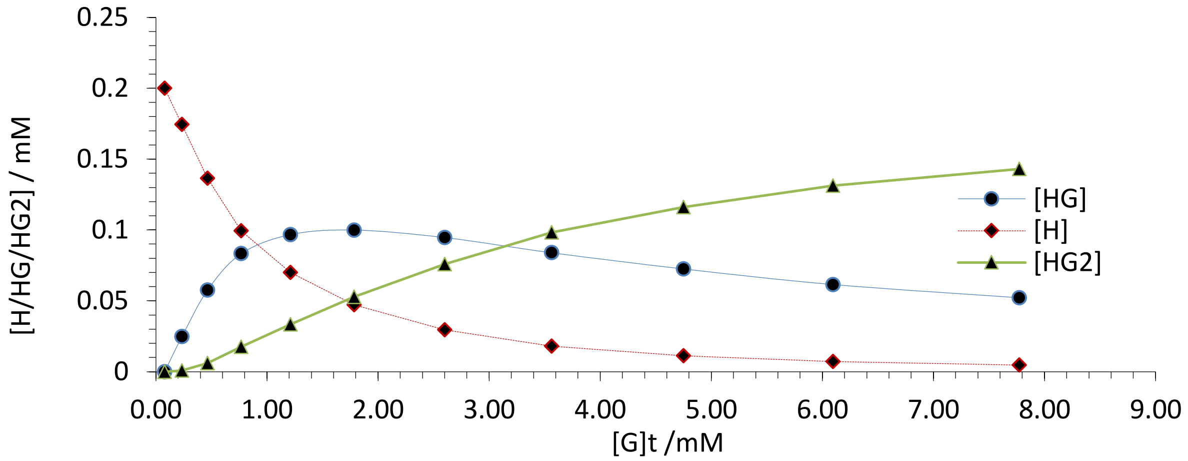

Figure S22. Species distribution resulting from analysis of the NMR binding study of receptor **9** with D-galactosamine **11** (see above).

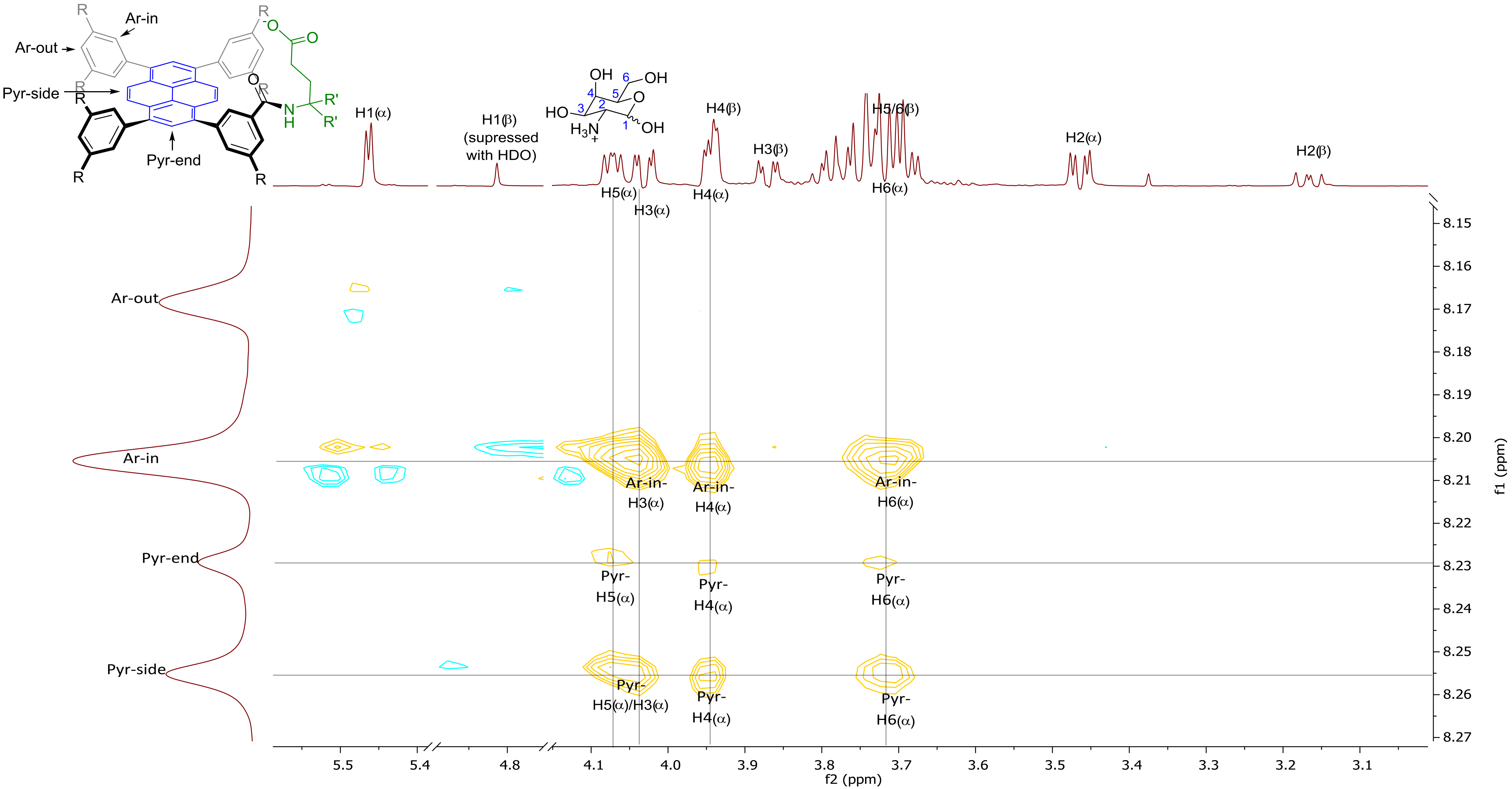

Figure S23. 2D-^{1}H-NOESY Spectrum of receptor **9** (0.40 mM) with D-galactosamine **11** (9.84 mM) in D_2O at pH 7. Mixing time of 300 ms. Receptor aromatic peaks are shown in the vertical and carbohydrate peaks are shown in the horizontal. Carbohydrate α/β ratio = 1:1.85. Carbohydrate peaks for both anomers are labelled, where H1(α) refers to the anomeric proton (H1) and the α-anomer. Dashed lines provide a guide to the apparent location of the cross peaks.

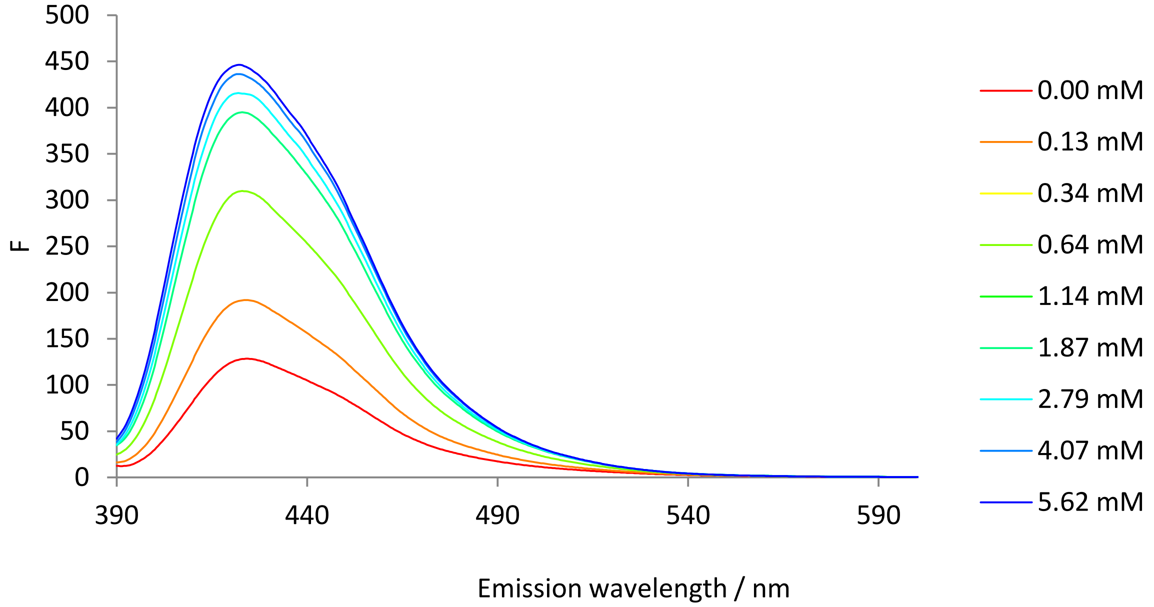

Figure S24. Fluorescence emission titration of receptor **9** (0.33 µM) with D-galactosamine **11** (15.7 mM) at pH 7 in H_2O at 298 K, excitation wavelength 380 nm.

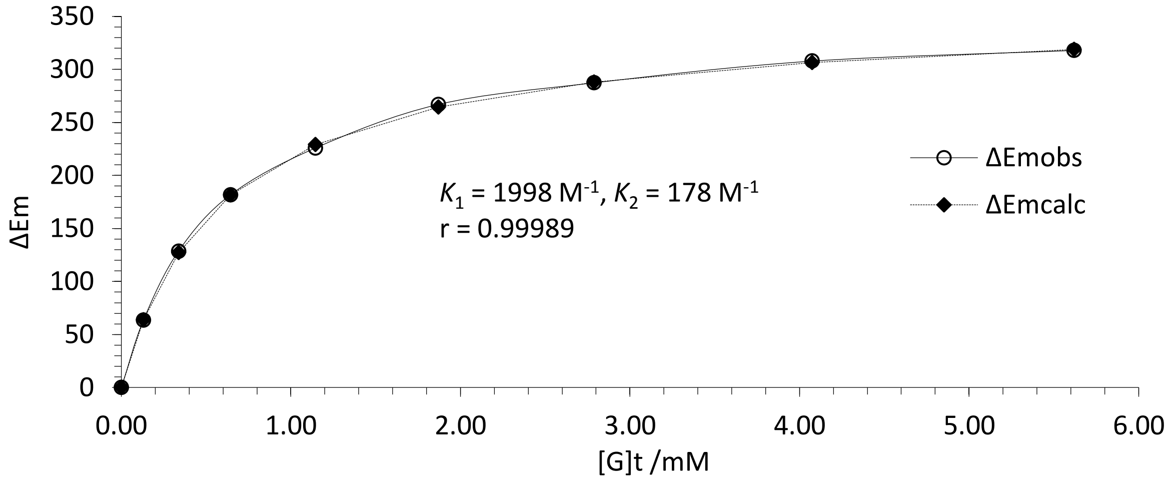

Figure S25. Data analysis for fluorescence binding study of receptor **9** titrated with D-galactosamine **11** (see above). Plot of observed and predicted emission intensity (423 nm) against guest concentration (mM), in accordance with a receptor:substrate 1:2 binding model with K_1 = 1998 M^{-1} and K_2 = 178 M^{-1}. Limiting fluorescence at 423 nm is Em_{HG} = 430 and Em_{HG2} = 493. r = 0.99989.

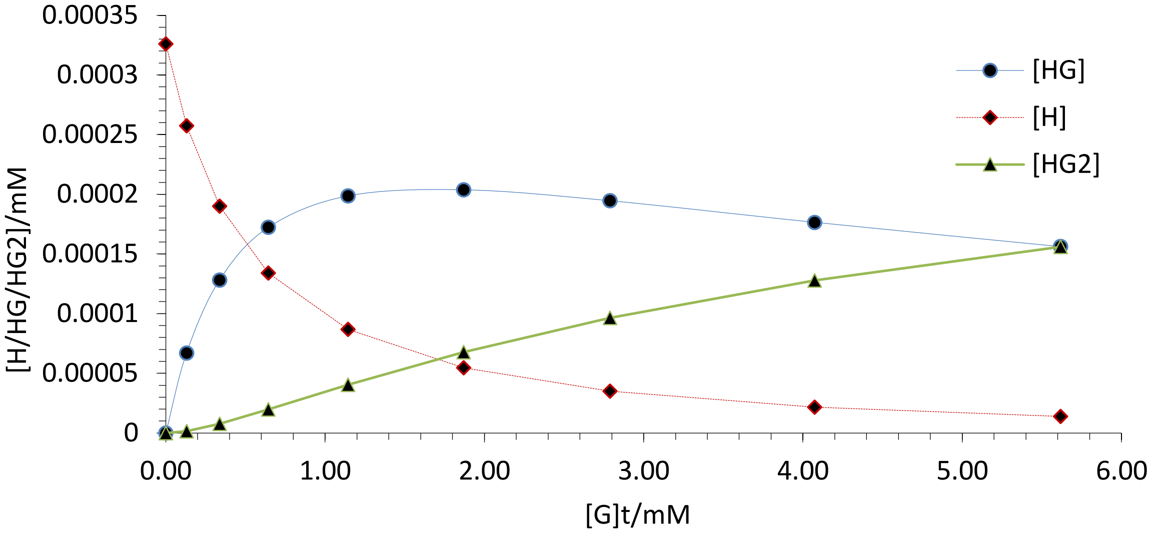

Figure S26. Species distribution resulting from analysis of the fluorescence binding study of receptor **9** with D-galactosamine **11** (see above).

Glucosamine 12

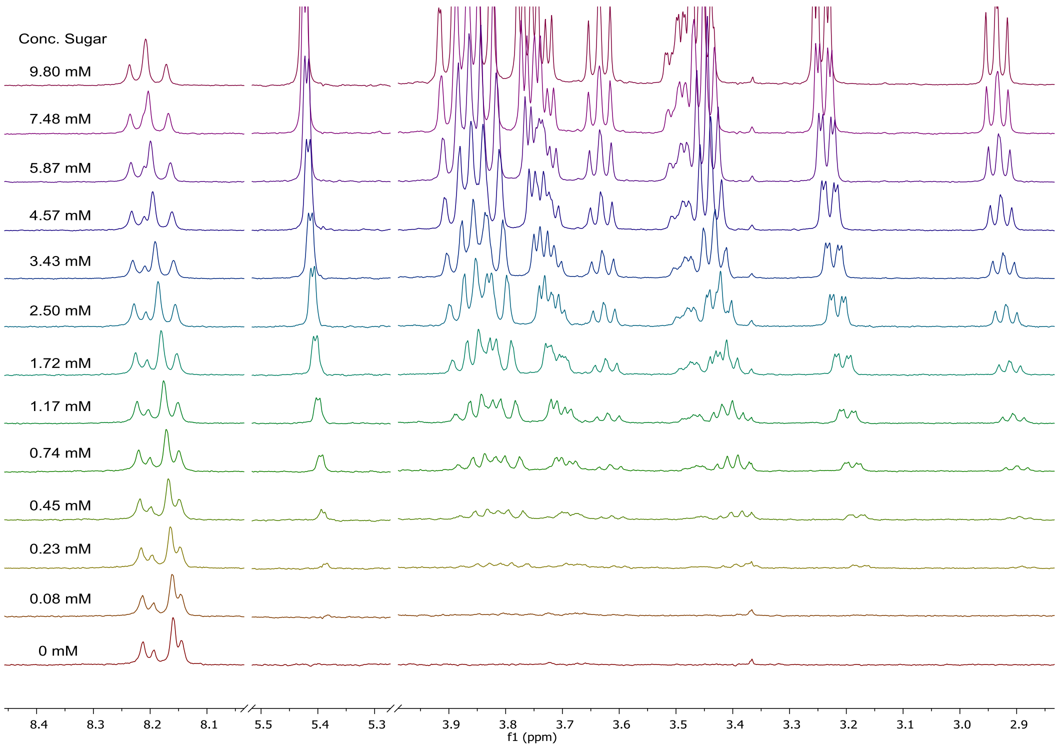

Figure S27. Partial spectra from a ^1H NMR titration of receptor **9** (0.20 mM) with D-glucosamine **12** (30.3 mM) in D_2O at pH 7.

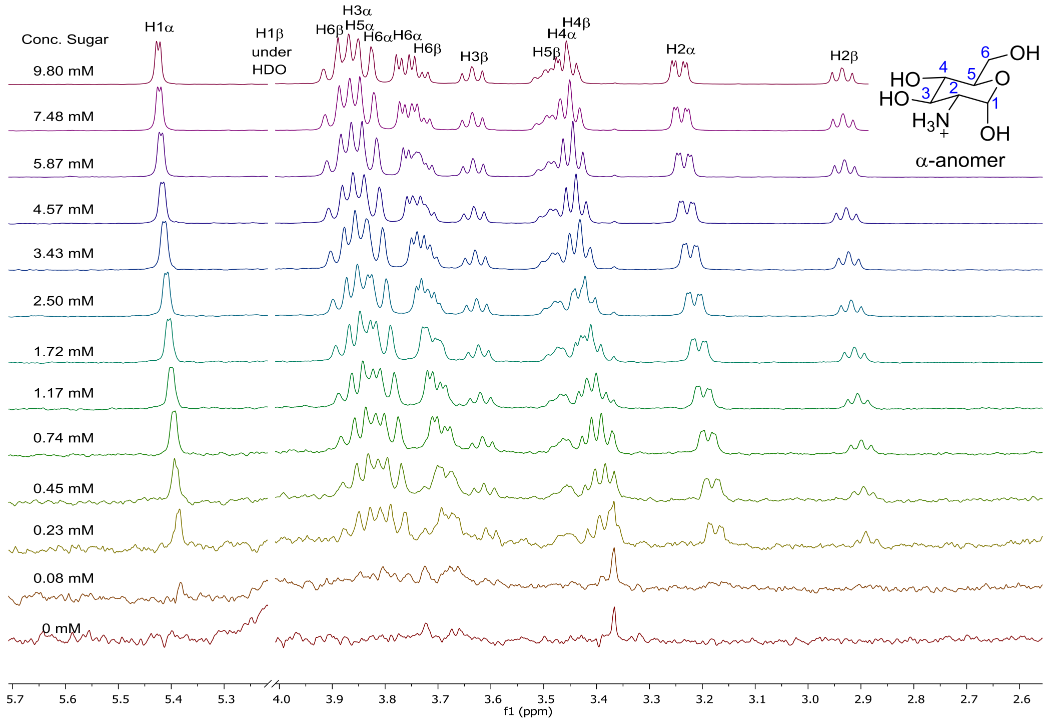

Figure S28. Partial spectra from a ^1H NMR titration of receptor **9** (0.20 mM) with D-glucosamine **12** (30.3 mM) in D_2O at pH 7 (as above, but rescaled to standardise carbohydrate signals). Carbohydrate α/β ratio = 1:0.60.

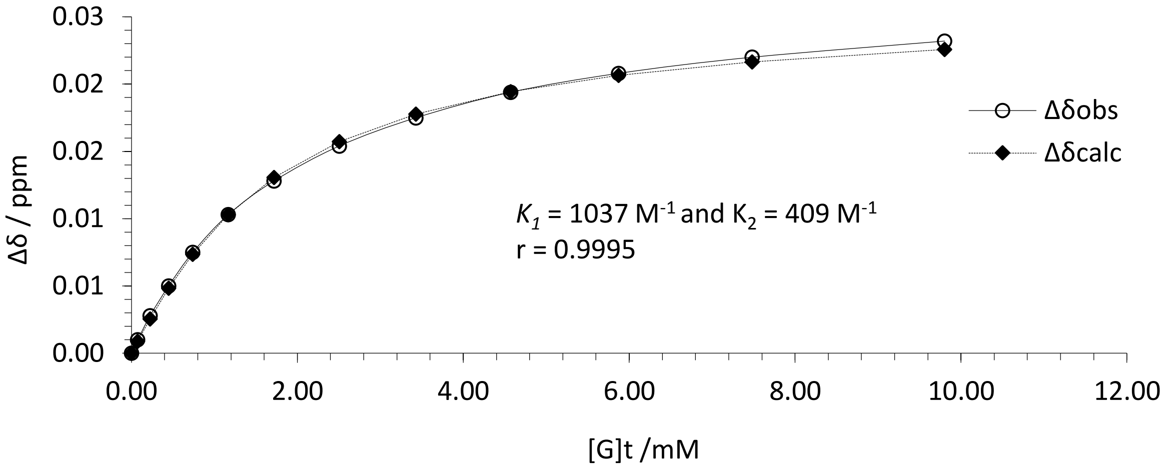

Figure S29. Data analysis for ^{1}H NMR titration of receptor **9** with D-glucosamine **12** (see above). Plot of observed and predicted changes in chemical shift (ppm) against guest concentration (mM), in accordance with a receptor:substrate 1:2 binding model with K_1 = 1037 M^{-1} and K_2 = 409 M^{-1}. Initial chemical shift is δH = 8.2130 ppm. Limiting chemical shifts are δHG = 8.2243 and δHG_2 = 8.2389 ppm. r = 0.9995.

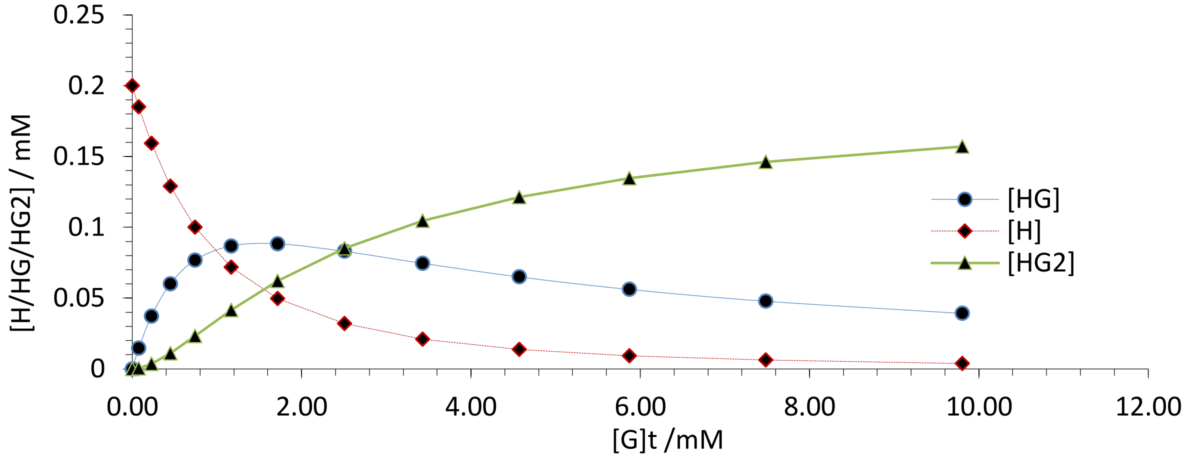

Figure S30. Species distribution resulting from analysis of the NMR binding study of receptor **9** titrated with D-glucosamine **12** (see above).

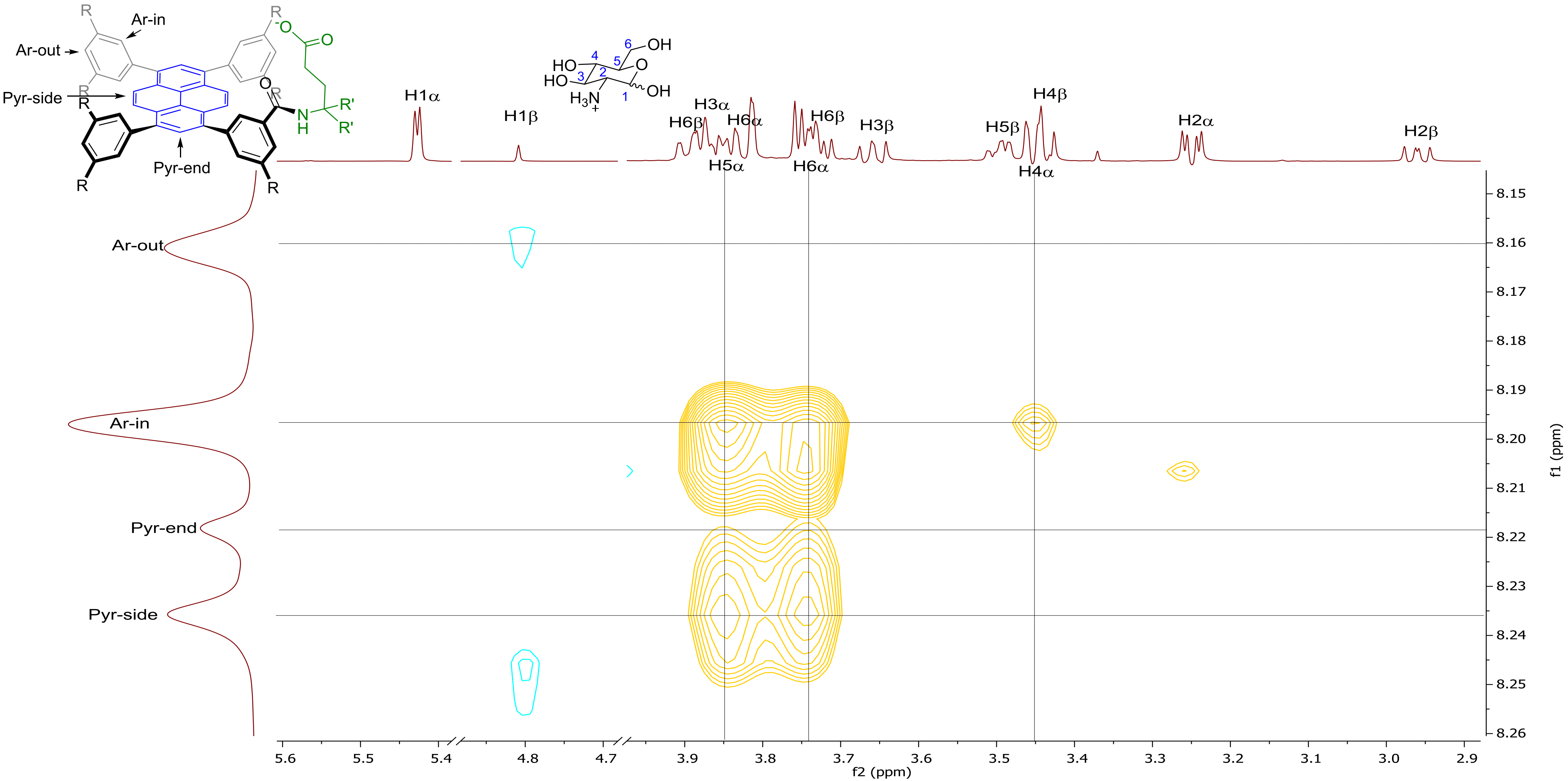

Figure S31. 2D-^{1}H-NOESY Spectrum of receptor **9** (0.40 mM) with D-glucosamine **12** (9.19 mM) in D_2O at pH 7. Mixing time of 300 ms. Receptor aromatic peaks are shown in the vertical and carbohydrate peaks are shown in the horizontal. Carbohydrate α/β ratio = 1:0.60. Carbohydrate peaks for both anomers are labelled, where H1(α) refers to the anomeric proton (H1) and the α-anomer. Dashed lines provide a guide to the apparent location of the cross peaks. The connections suggest that H5 and H6 are especially close to the pyrene nucleus, possibly due to a tilted geometry in which the ammonium group is held away from the pyrene.

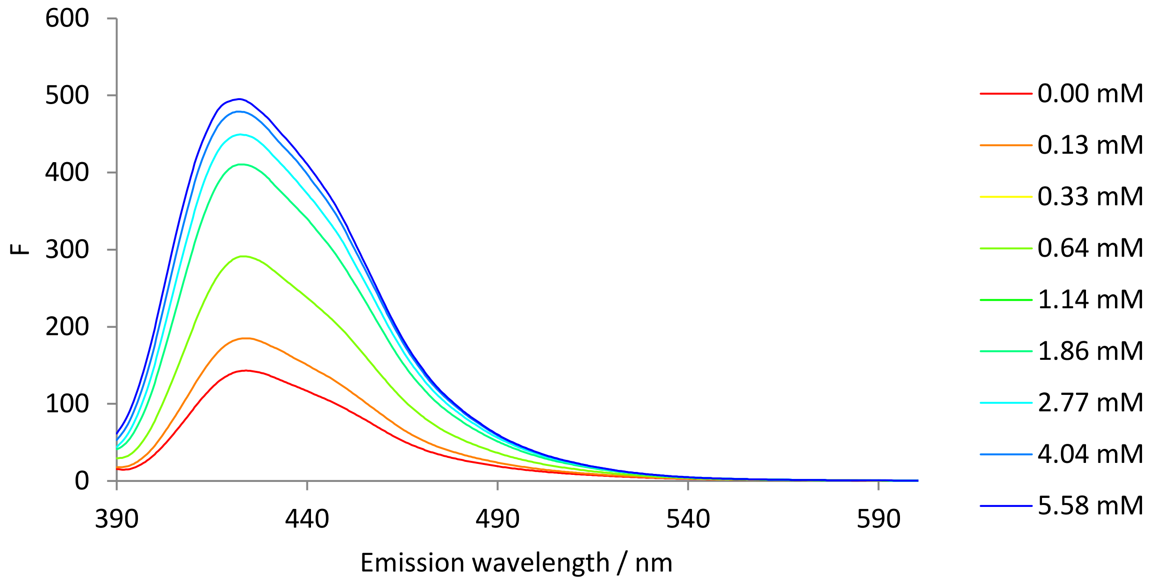

Figure S32. Fluorescence emission titration of receptor **9** (0.33 µM) with D-glucosamine **12** (15.5 mM) at pH 7 in H_2O at 298 K, excitation wavelength 380 nm.

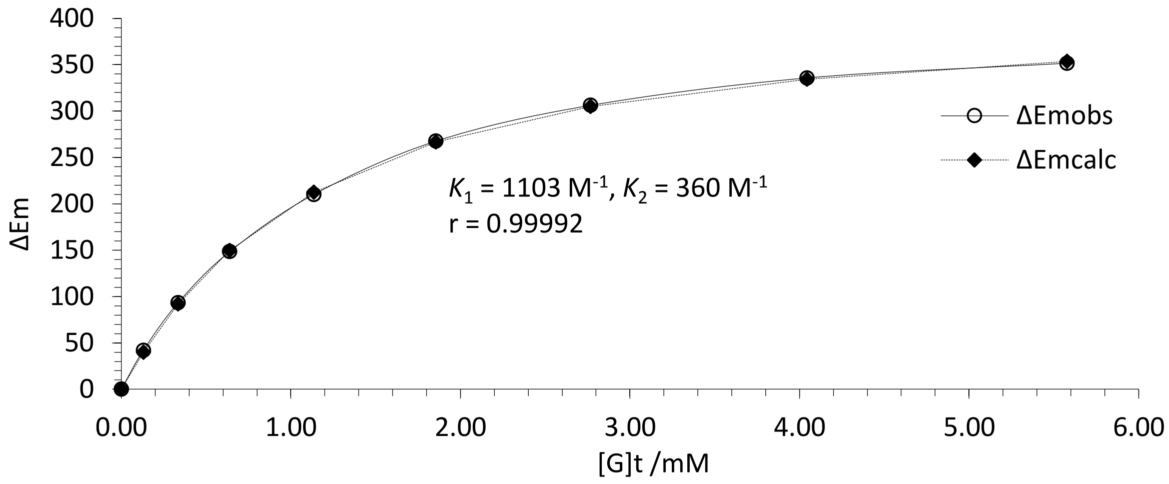

Figure S33. Data analysis for fluorescence binding study of receptor **9** titrated with D-glucosamine **12** (see above). Plot of observed and predicted emission intensity (423 nm) against guest concentration (mM), in accordance with a receptor:substrate 1:2 binding model with K_1 = 1103 M^{-1} and K_2 = 360 M^{-1}. Limiting fluorescence at 423 nm is Em_{HG} = 446 and Em_{HG2} = 550. r = 0.99992.

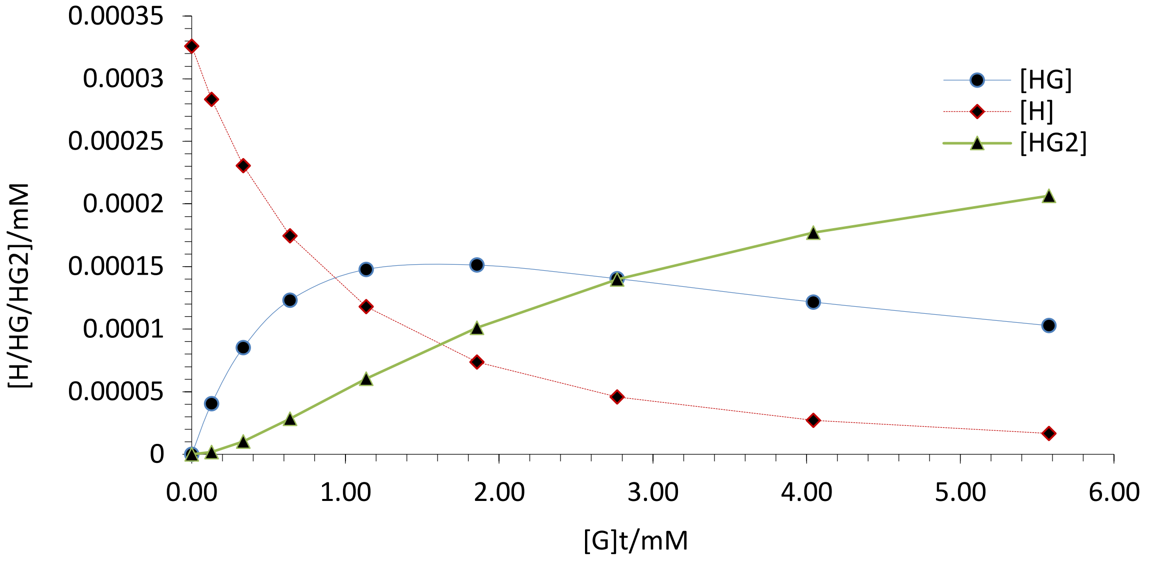

Figure S34. Species distribution resulting from analysis of the fluorescence binding study of receptor **9** titrated with D-glucosamine **12** (see above).

Cellobiose

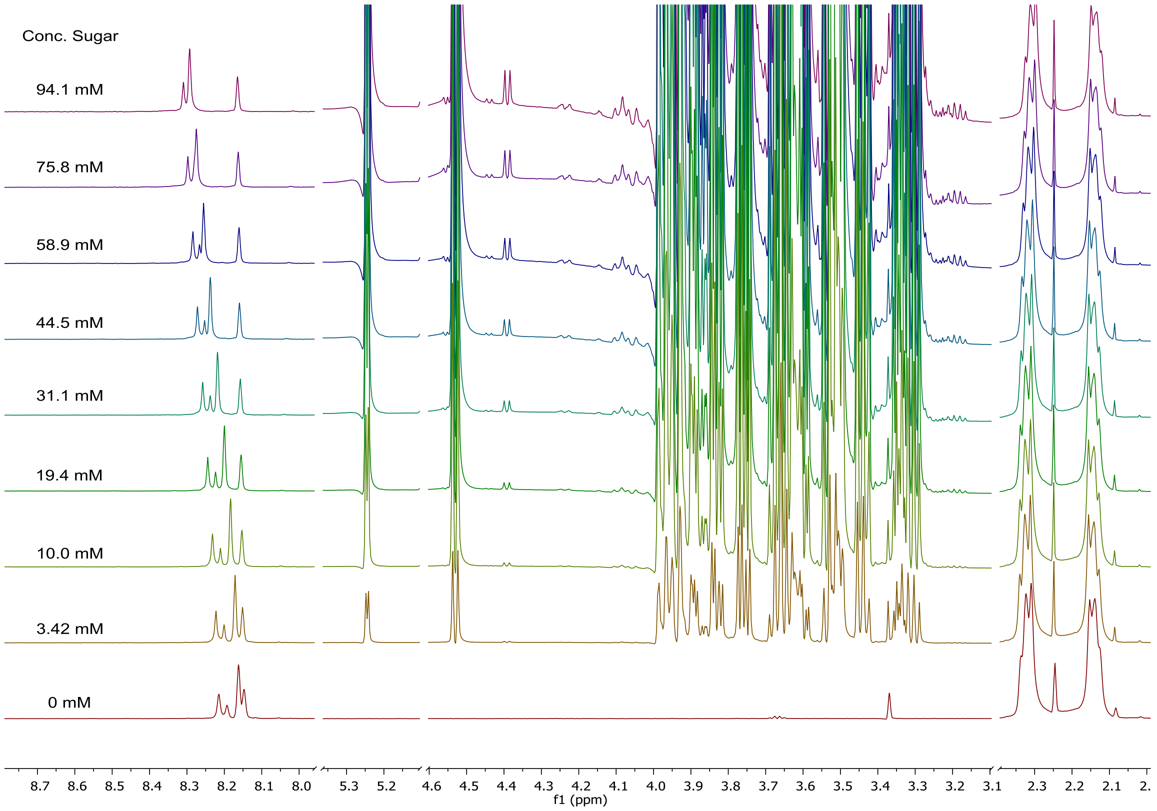

Figure S35. Partial spectra from a ^{1}H NMR titration of receptor **9** (0.20 mM) with cellobiose (311 mM) at pH 7 in D_2O.

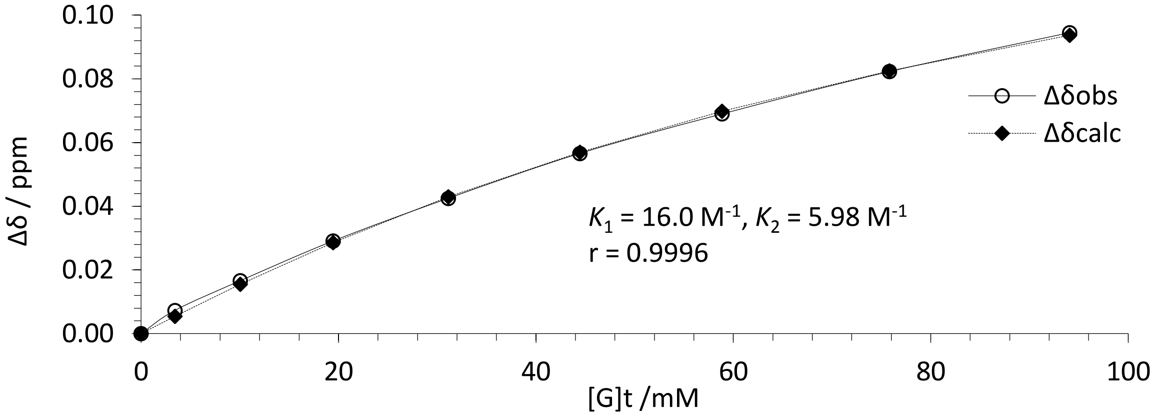

Figure 36. Data analysis for ^{1}H NMR titration of receptor **9** with cellobiose (see above). Plot of observed and predicted changes in chemical shift (ppm) against guest concentration (mM), in accordance with a receptor:substrate 1:2 binding model with K_1 = 16.0 M^{-1} and K_2 = 5.98 M^{-1}. Initial chemical shift is δH = 8.2150 ppm. Limiting chemical shifts are δHG = 8.3168 and δHG_2 = 8.4048 ppm. r = 0.9996.

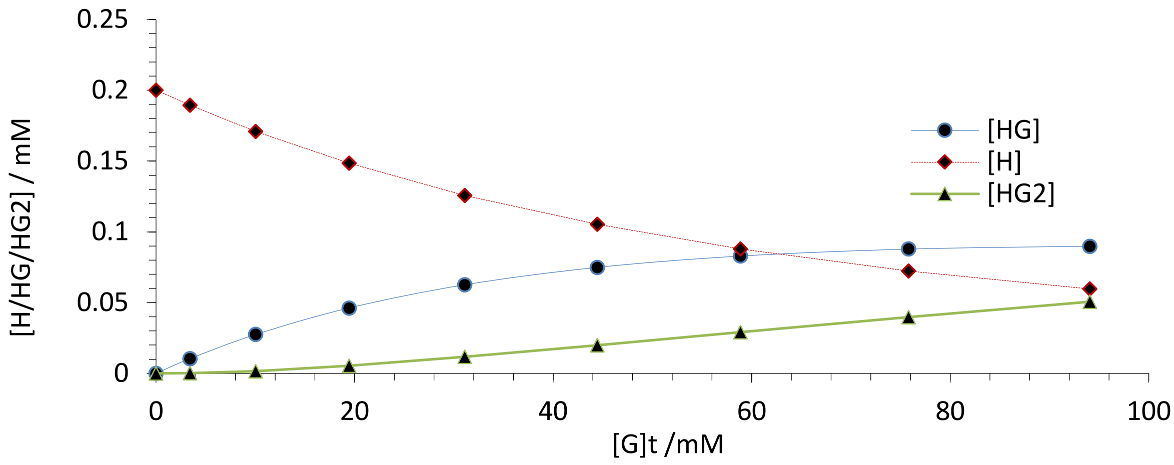

Figure 37. Species distribution resulting from analysis of the NMR binding study of receptor **9** with cellobiose (see above).

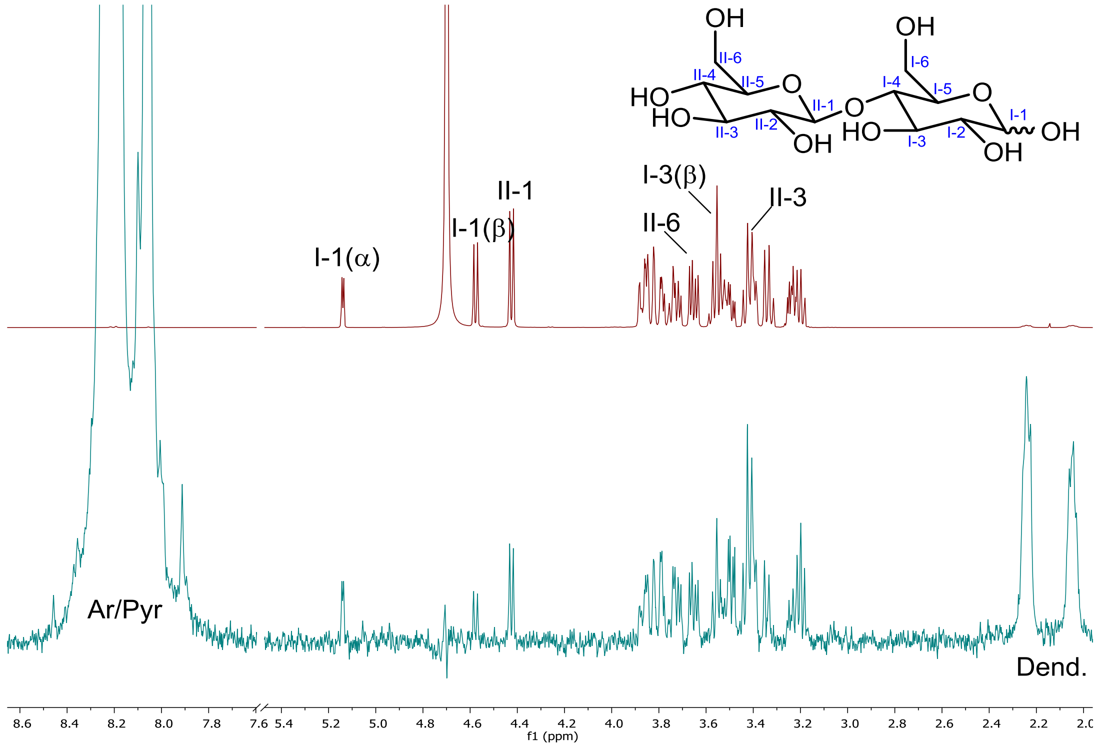

Figure S38. ^{1}H NMR (top) stacked with ^{1}H NOESY NMR spectra (bottom) of receptor **9** (0.20 mM) with cellobiose (94.1 mM) at pH 7 in D_2O at 298 K. Mixing time = 300 ms. Aromatic receptor peaks excited, showing correlation with dendrimers and sugar peaks. Selected carbohydrate peaks for both anomers are labelled, where I-1(α) refers to the anomeric proton (H1) in the α-anomer of the reducing sugar unit. A significant difference in correlation intensity can be observed between II-3 and I-3(β), suggesting that the non-reducing monosaccharide is in closer proximity to the receptor aromatic protons.

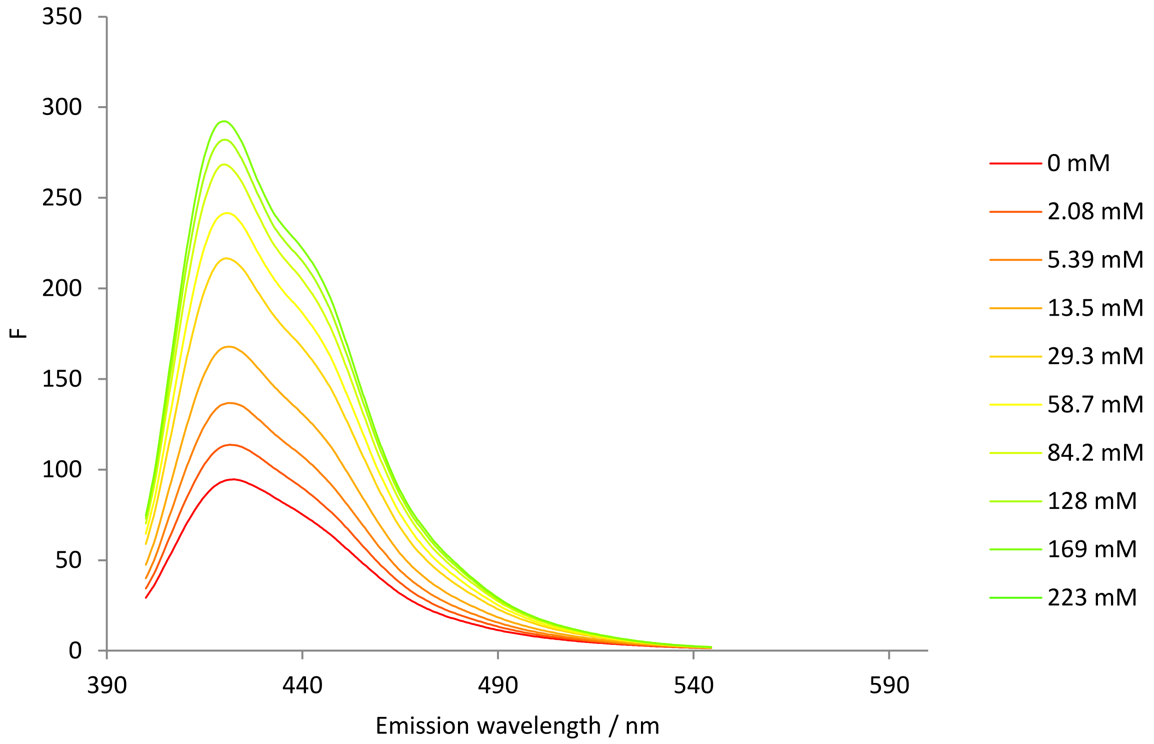

Figure S39. Fluorescence emission titration of receptor **9** (0.13 µM) with cellobiose (250 mM) at pH 7 in H_2O at 298 K, excitation wavelength 380 nm.

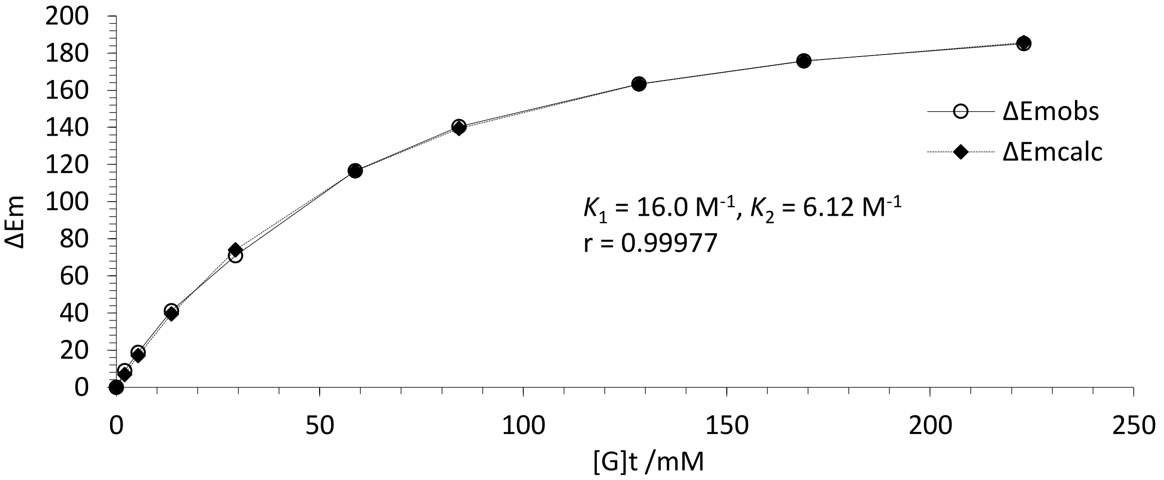

Figure S40. Data analysis for fluorescence binding study of receptor **9** with cellobiose (see above), in accordance with a receptor:substrate 1:2 binding model with K_1 = 15.7 M^{-1} and K_2 = 4.42 M^{-1}. Limiting fluorescence at 423 nm is Em_{HG} = 301 and Em_{HG2} = 301. r = 0.99977.

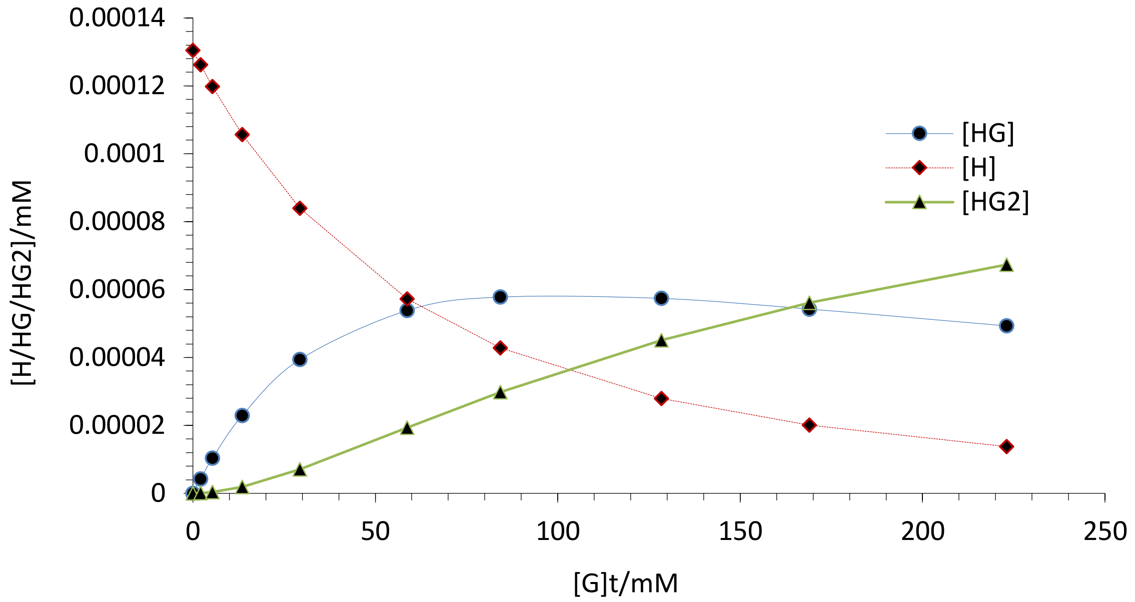

Figure S41. Species distribution resulting from analysis of the fluorescence binding study of receptor **9** titrated with cellobiose (see above).

Lactose

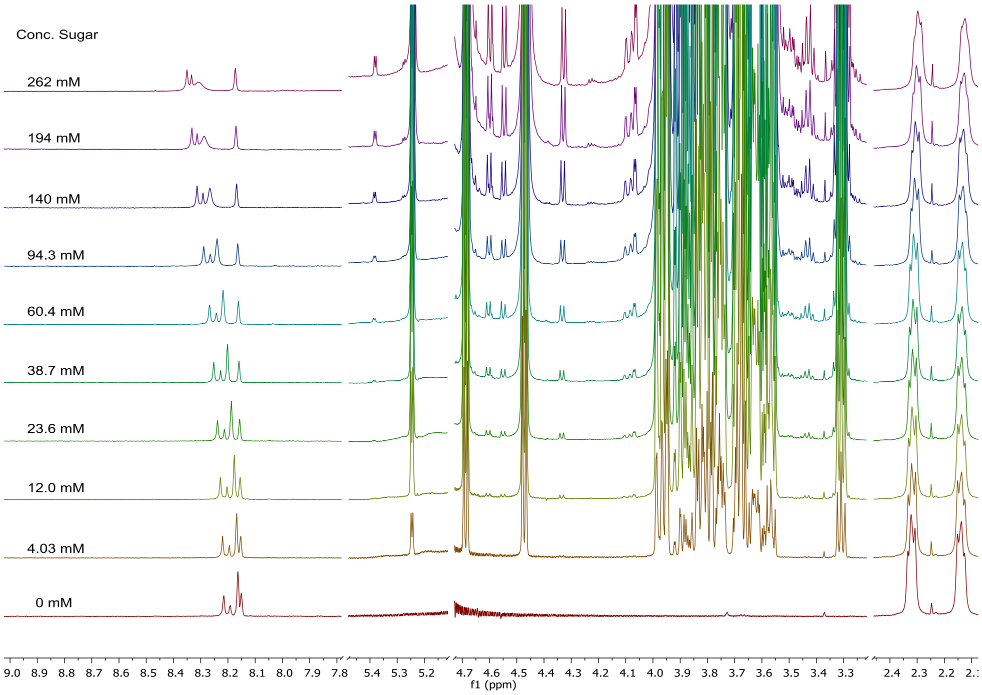

Figure S42. Partial spectra from a ^{1}H NMR titration of receptor **9** (0.20 mM) with lactose (910 mM) at pH 7 in D_2O. The receptor aromatic signals move appreciably during the titration, but the plot of $\Delta\delta$ vs. concentration is only slightly curved implying that binding is very weak.

Maltose

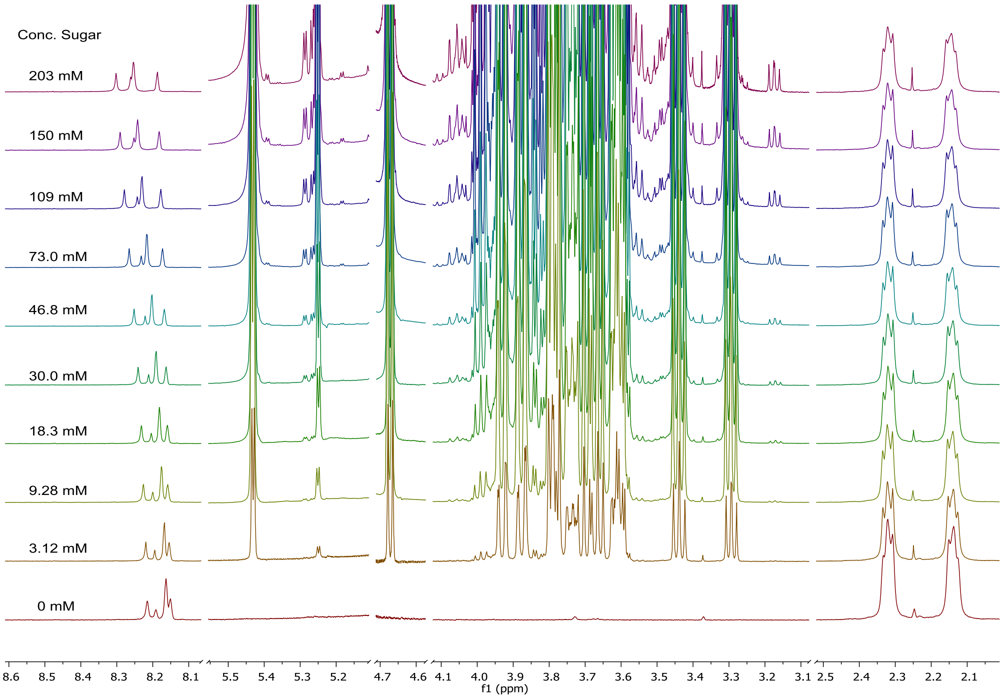

Figure S43. Partial spectra from a ^{1}H NMR titration of receptor **9** (0.20 mM) with maltose (705 mM) at pH 7 in D_2O. The receptor aromatic signals move appreciably during the titration, but the plot of $\Delta\delta$ vs. concentration is only slightly curved implying that binding is very weak.

Methylamine

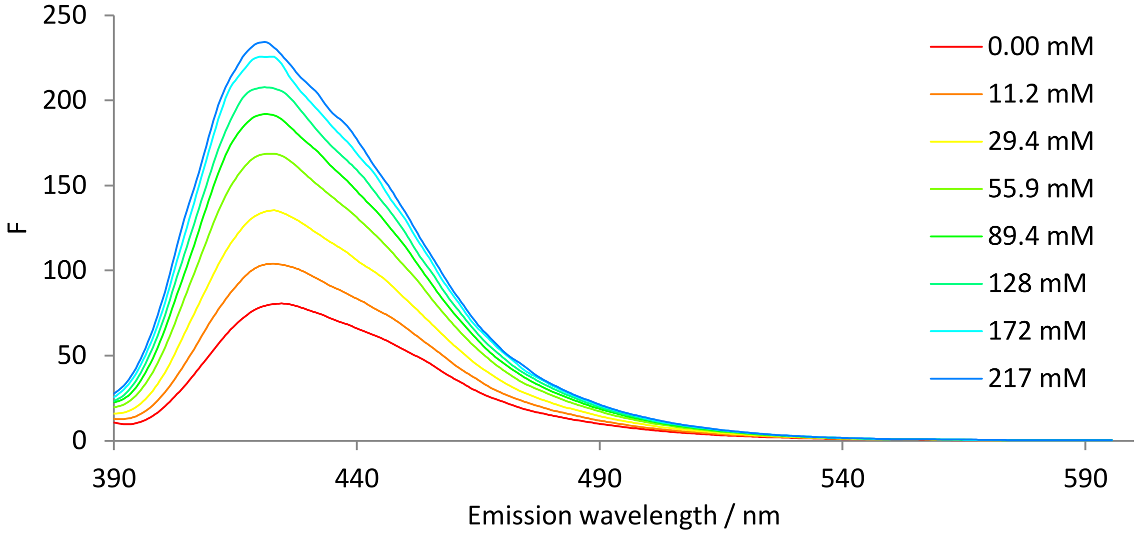

Figure S44. Fluorescence emission titration of receptor **9** (0.33 µM) with methylamine (559 mM) at pH 7 in H_2O at 298 K, excitation wavelength 380 nm.

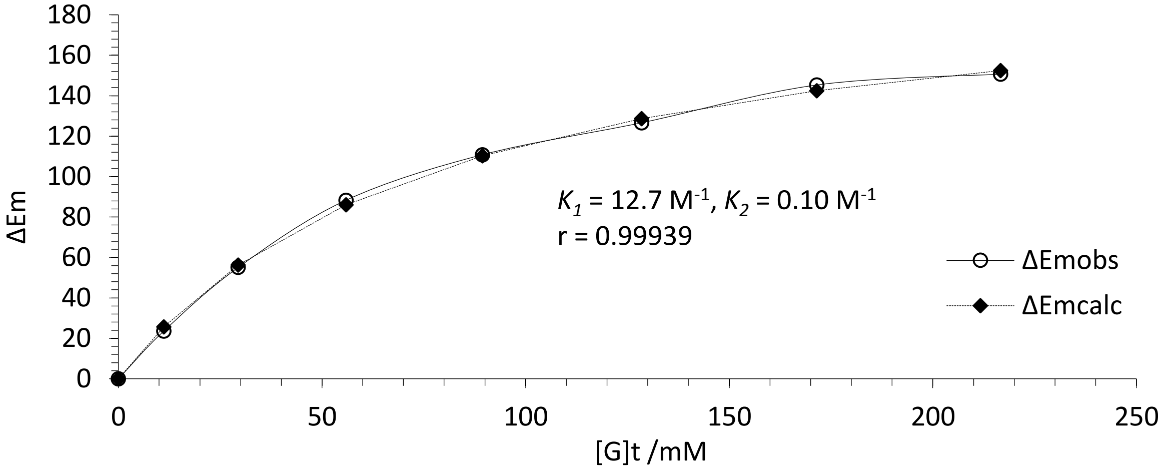

Figure S45. Data analysis for fluorescence binding study of receptor **9** with methylamine (see above), in accordance with a receptor:substrate 1:2 binding model with K_1 = 12.7 M^{-1} and K_2 = 0.10 M^{-1}. Limiting fluorescence at 423 nm is Em_{HG} = 287 and Em_{HG2} = 287. r = 0.99939.

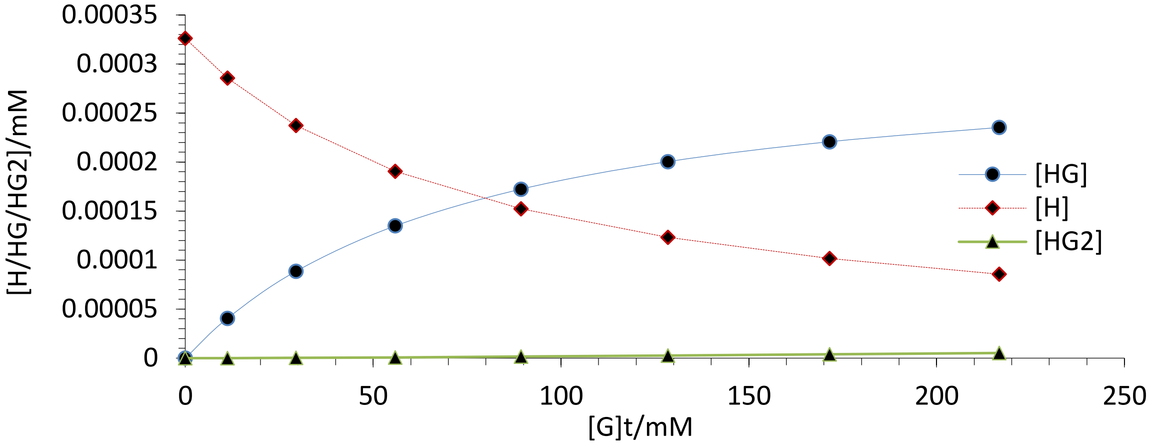

Figure S46. Species distribution resulting from analysis of the fluorescence binding study of receptor **9** titrated with methylamine (see above).

Cationic Receptor 16 – Spectroscopic Studies

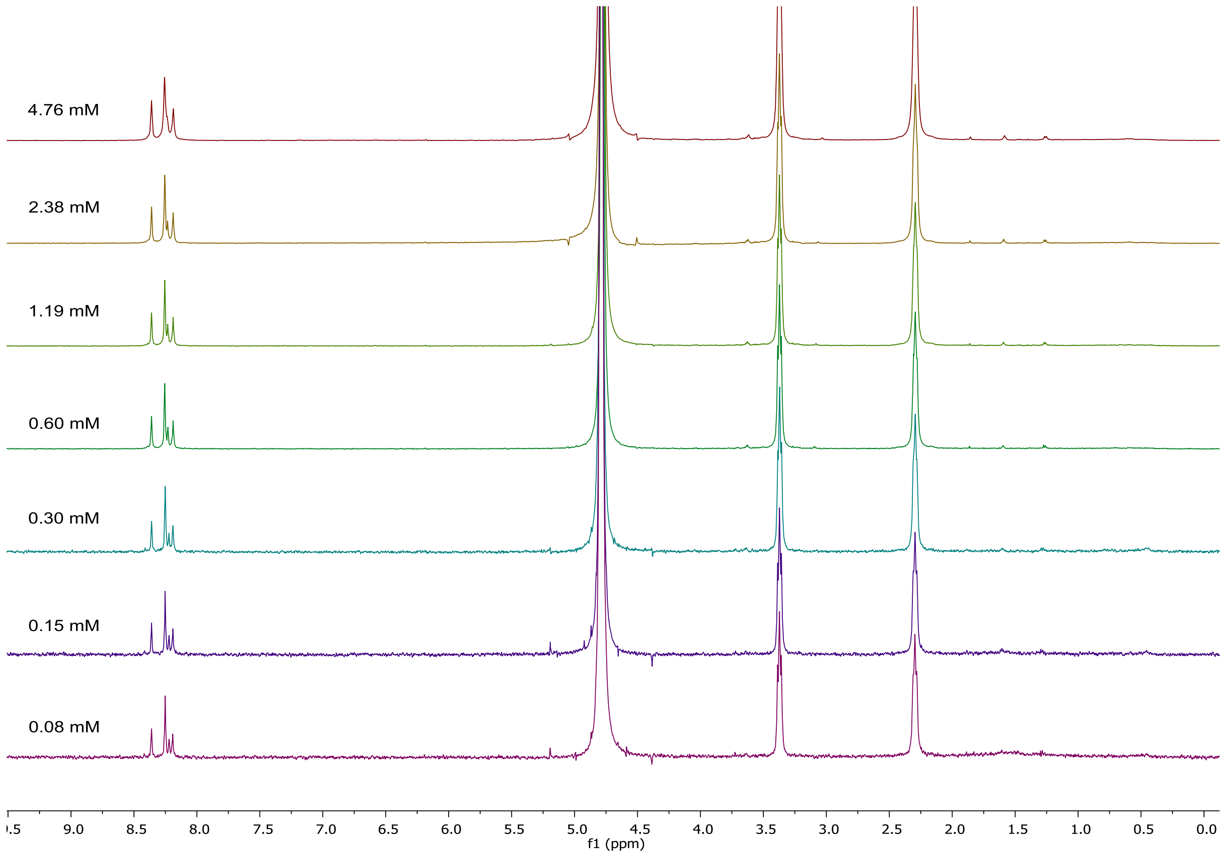

Figure S47. ^{1}H-NMR spectra of receptor **16** at a range of concentrations, 0.08 mM to 4.76 mM in D_2O at 298 K.

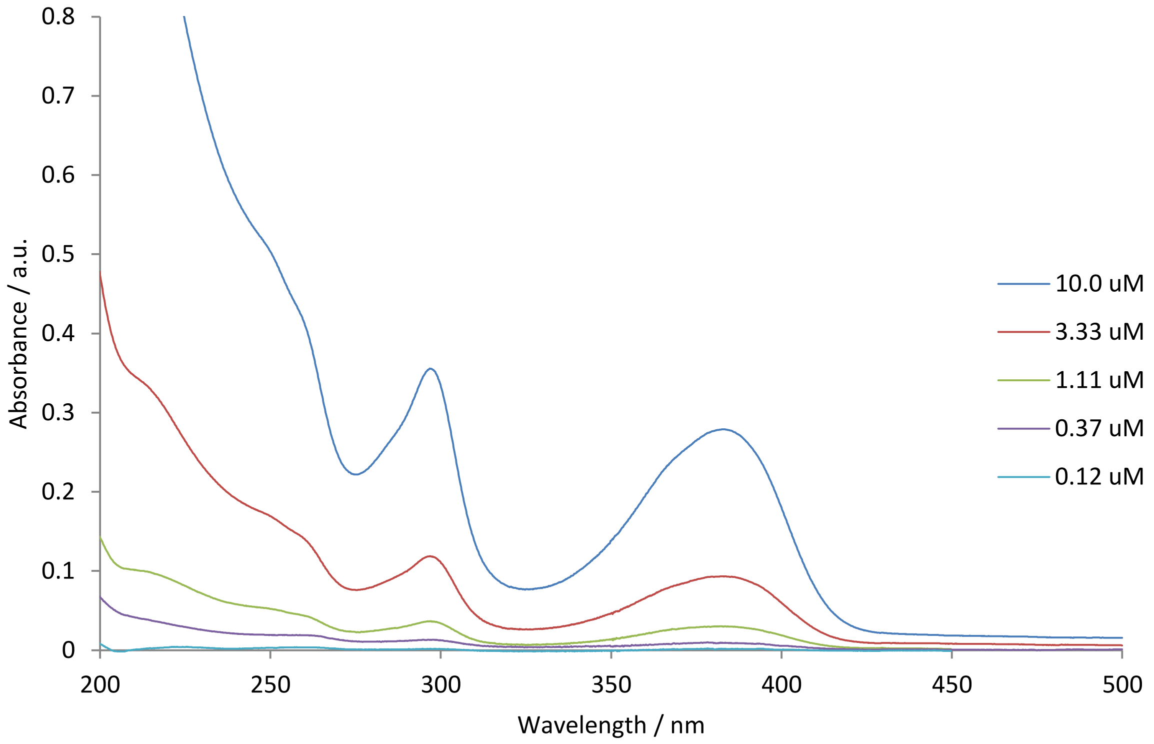

Figure S48. UV-Vis Spectra of receptor **16** in H_2O over a range of concentrations (10.0 to 0.12 µM) at 298 K.

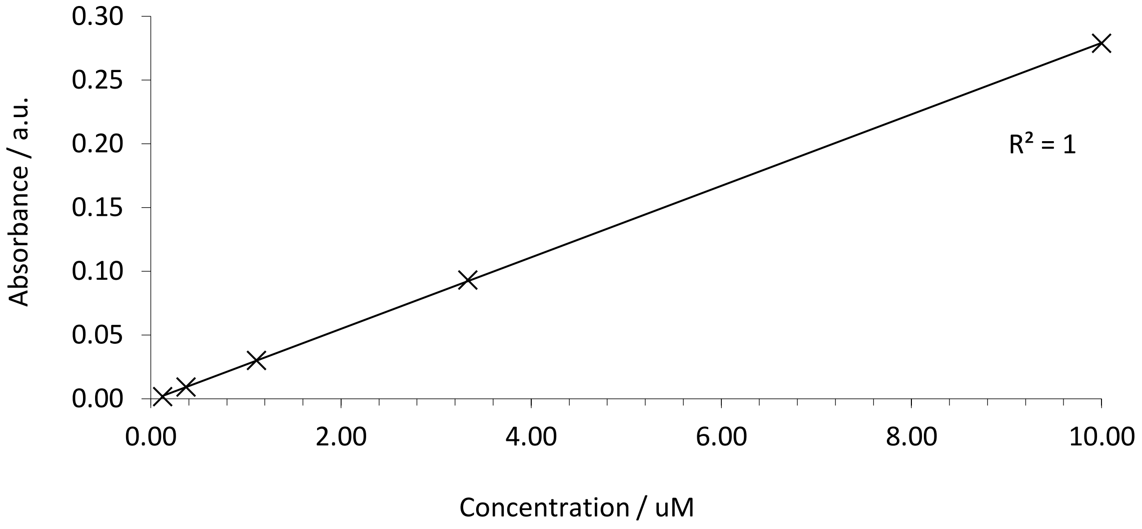

Figure S49. UV-Vis absorbance of receptor **16** at 380 nm in H_2O over a range of concentrations (10.0 to 0.12 µM) at 298 K. A linear relationship between concentration and absorbance is observed.

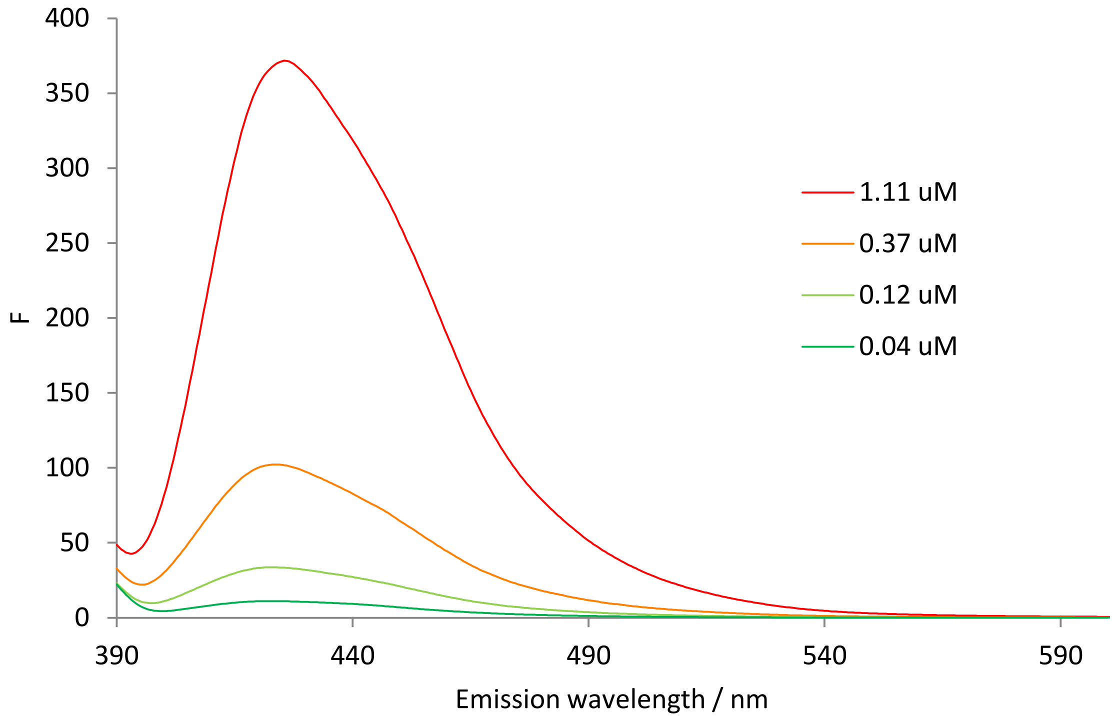

Figure S50. Fluorescence emission spectra of receptor **16** in H_2O at a range of concentrations (1.10-0.03 µM), excited at 380 nm.

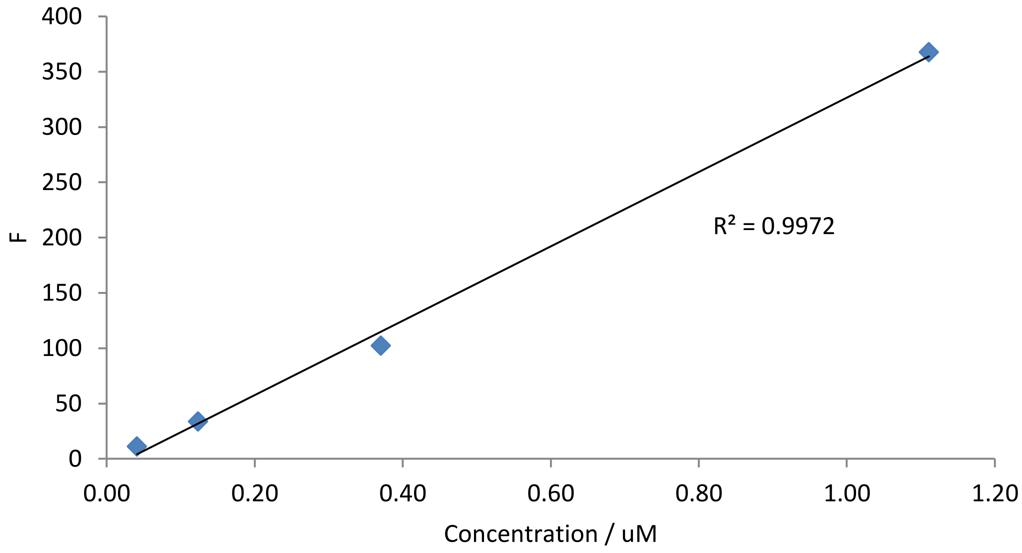

Figure S51. Fluorescence emission intensity of receptor **16** in H_2O over a range of concentrations (1.11 µM–0.04 µM), excited at 380 nm. A linear relationship between concentration and intensity is observed.

Cationic Receptor 16 – Binding Studies.

Methyl α-sialoside 14

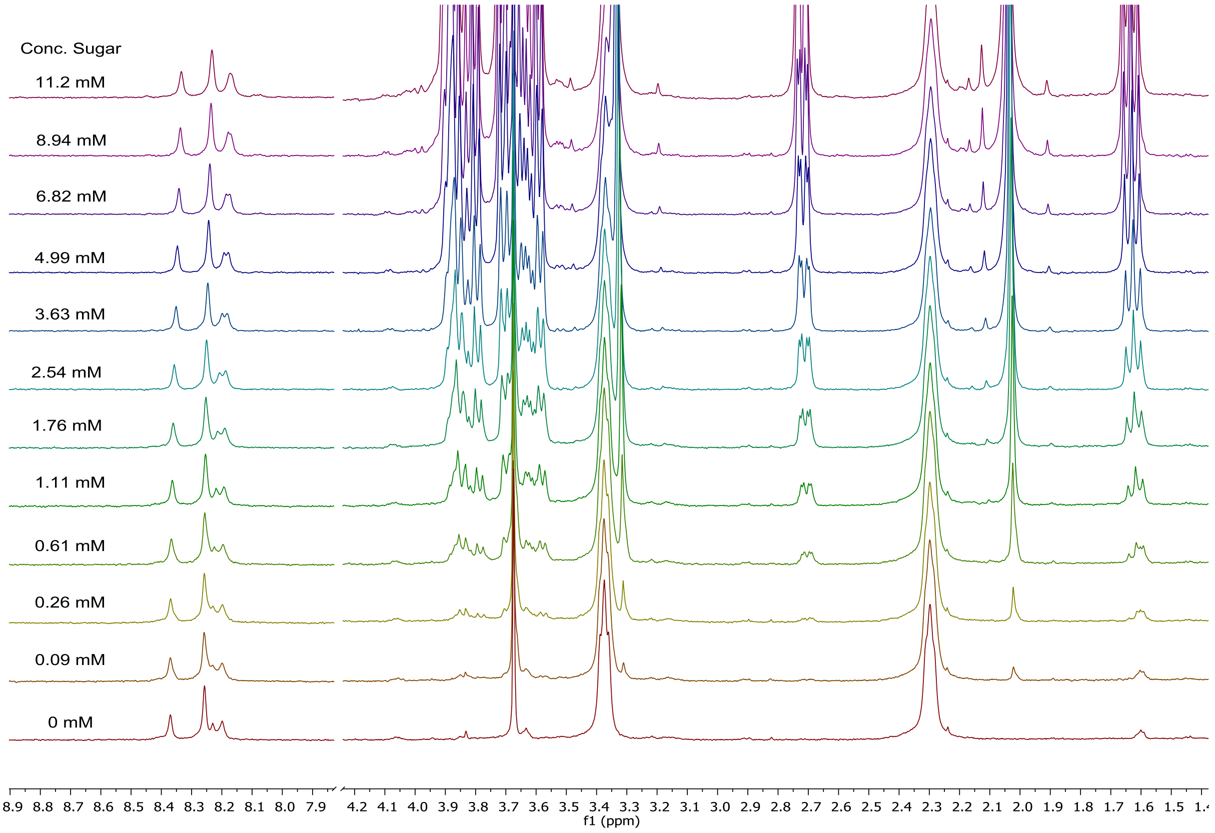

Figure S52. Partial ^{1}H NMR spectra from the binding study of receptor **16** (0.25 mM) titrated with methyl α-sialoside **14** (35.2 mM) at pH 7 in D_2O at 298 K.

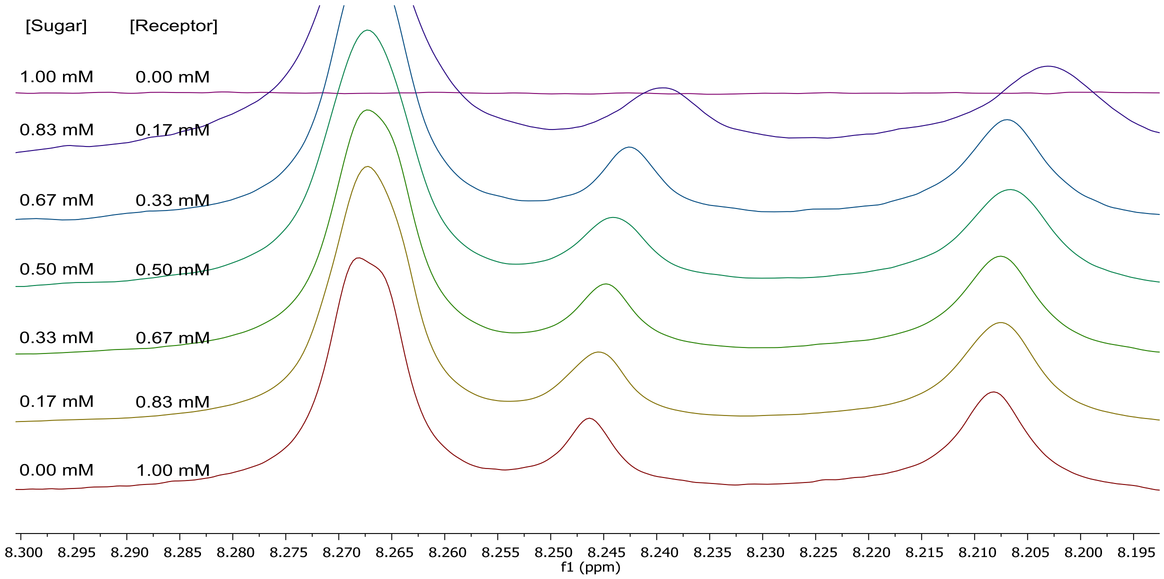

Figure S53. Partial ^{1}H NMR spectra of receptor **16** with methyl α-sialoside **14** in D_2O at 298 K and pH 7, at various mol fractions used for a Job Plot.

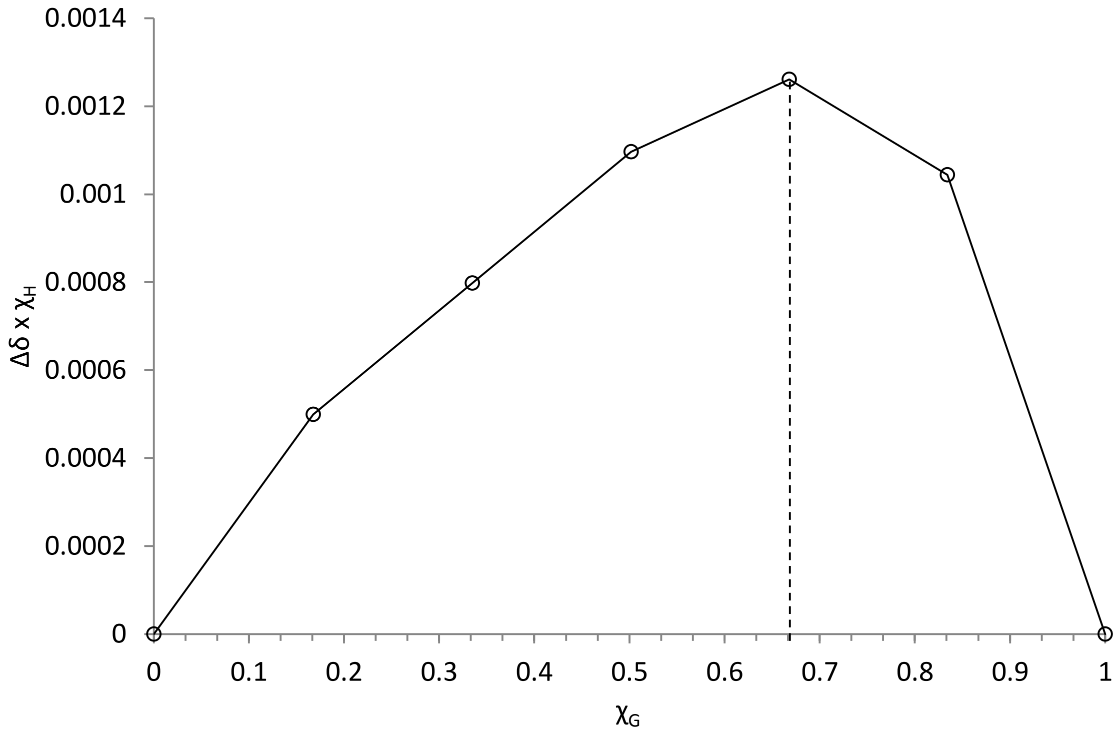

Figure S54. Job Plot of receptor **16** with methyl α-sialoside **14** in D_2O at 298 K and pH 7. X_G = mol fraction of sugar ([sugar]/[sugar]+[receptor]). Initial chemical shift δ_H = 8.2462 ppm. Maximum at X_G = 0.66 indicates a 1:2 (H:G) binding stoichiometry. Total [receptor] + [sugar] = 1 mM.

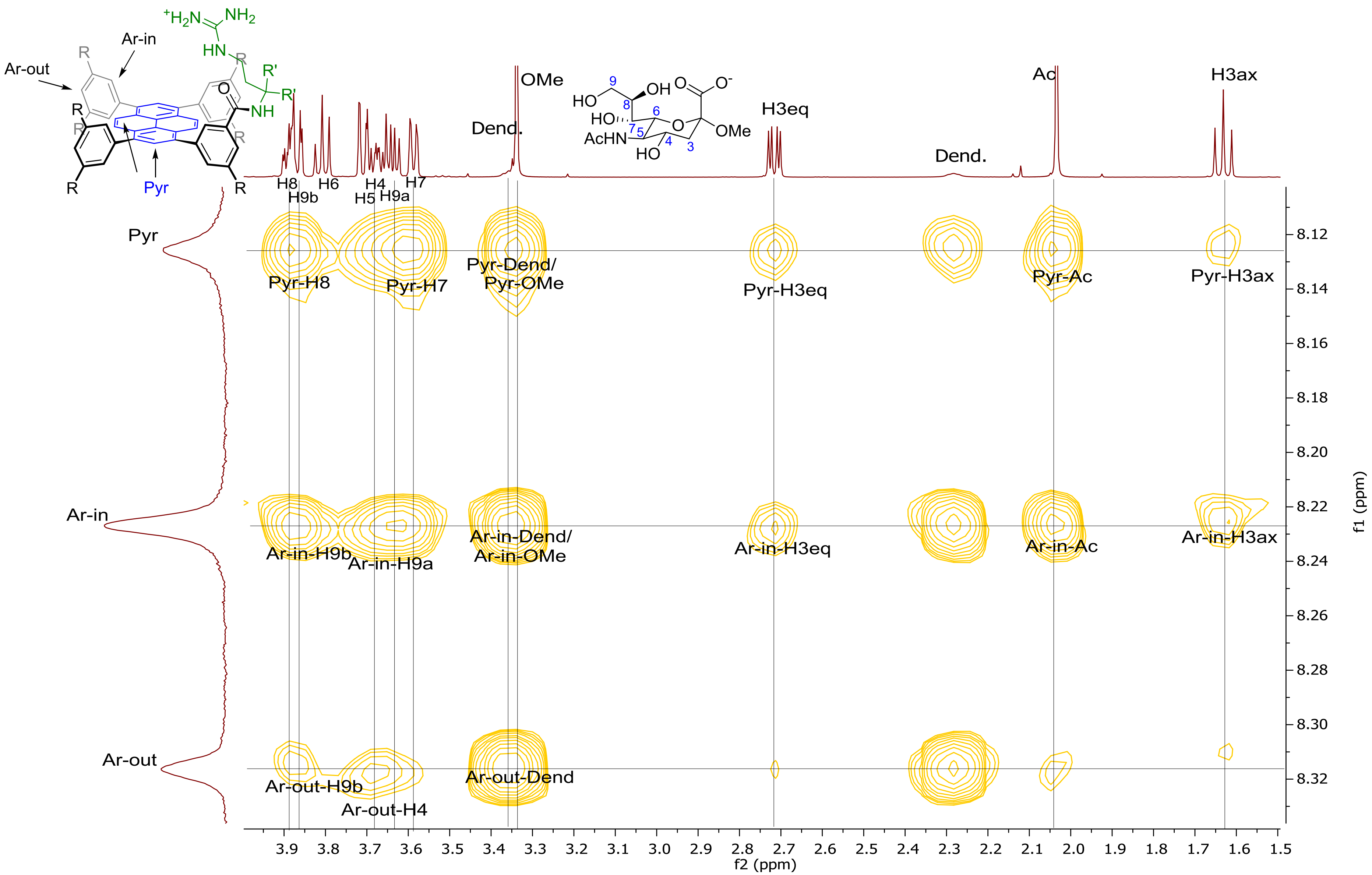

Figure S55. 2D-^{1}H-NOESY Spectrum of receptor **16** (0.20 mM) with methyl α-sialoside **14** (32.5 mM) in D_2O at pH 7 with a mixing time of 300 ms. Connections are observed between receptor aromatic protons and all regions of the substrate. As expected cross peaks to Ar-out, away from the binding site, are relatively weak.

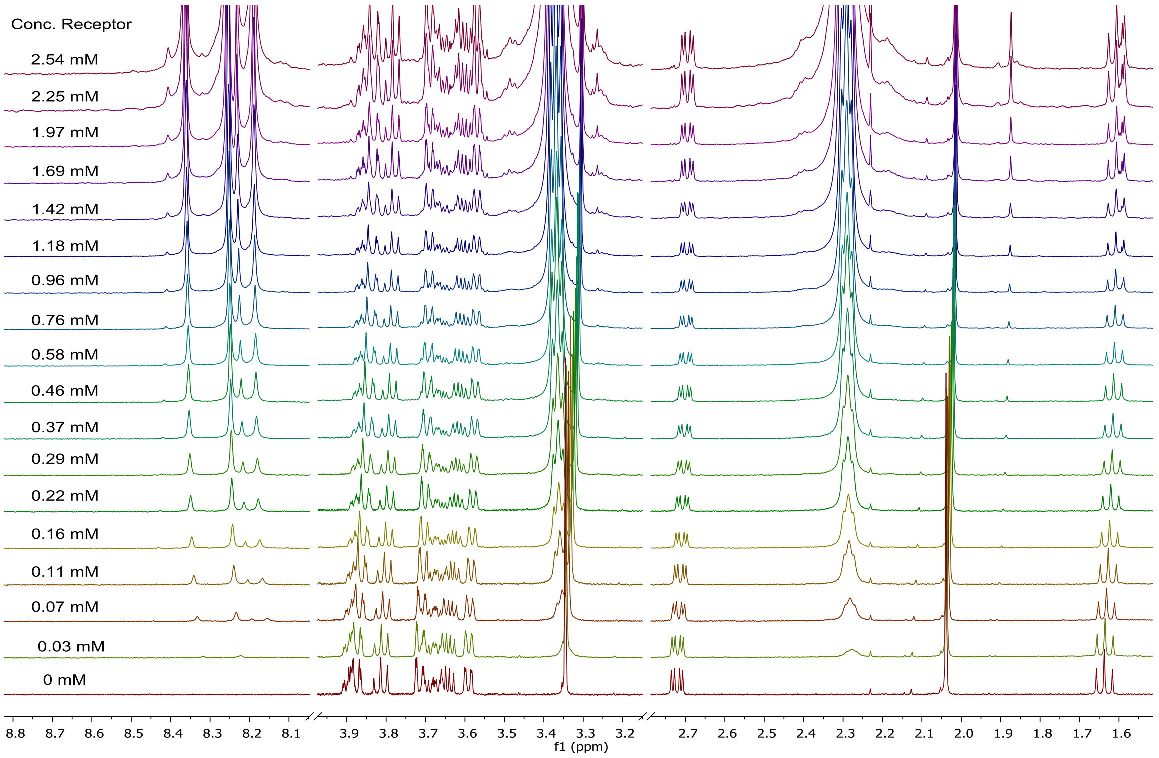

Figure S56. Partial ^{1}H NMR spectra from the binding study of methyl α-sialoside **14** (1.01 mM) titrated with receptor **16** (4.63 mM) at pH 7 in D_2O at 298 K.

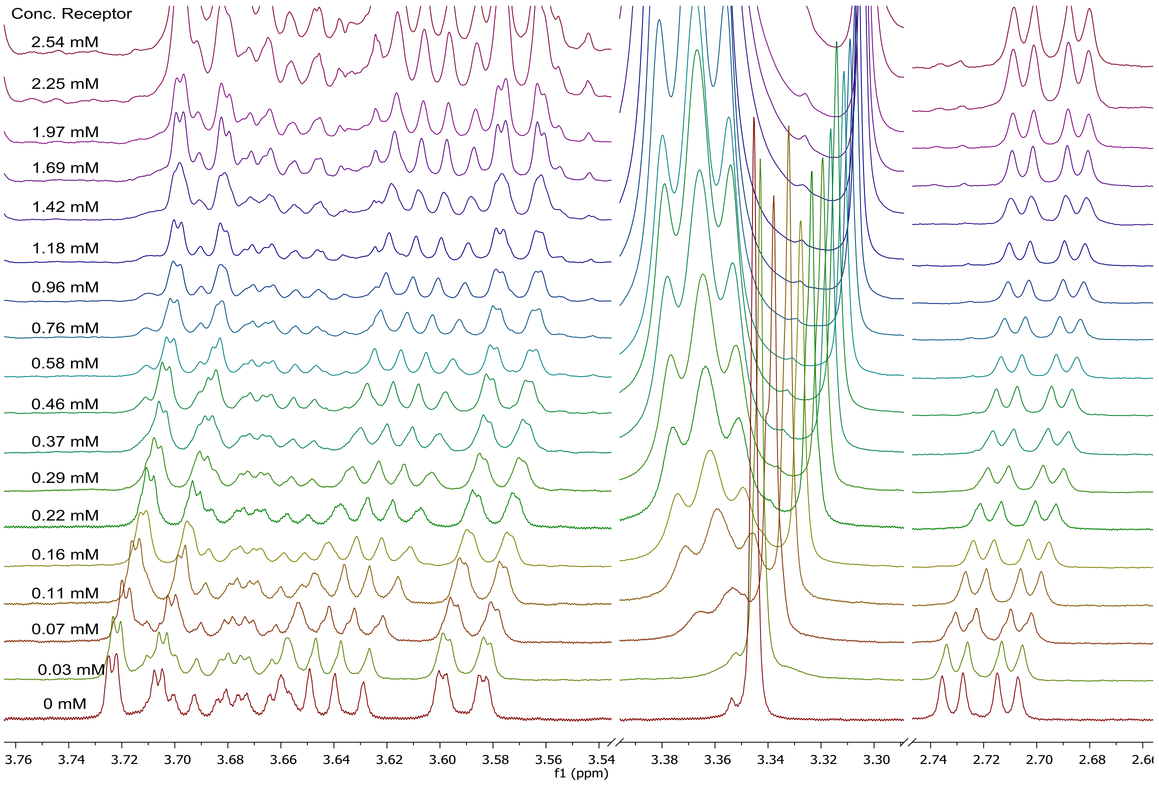

Figure S57. Expansions of selected regions from the spectra in Figure S56.

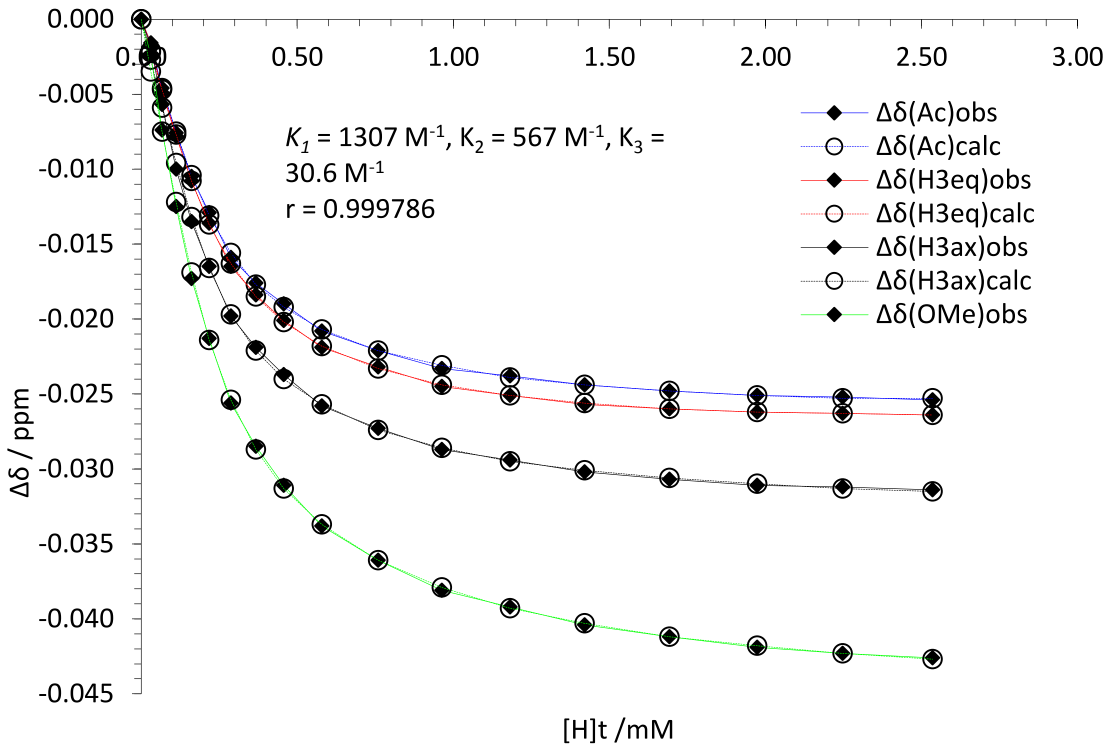

Figure S58. Data analysis for ^{1}H NMR titration of methyl α-sialoside **14** titrated with receptor **16** (see above). Observed and predicted changes in chemical shift (ppm) of guest are plotted against host concentration (mM). Changes in four sugar proton signals were simultaneously fitted to 1:3 binding model using HypNMR, giving K_1 = 1307 M^{-1}, K_2 = 567 M^{-1} and K_3 = 30.6 M^{-1}. Initial chemical shifts (H3ax, Ac, H3eq, OMe) are δG = 1.637, 2.0388, 2.7205 and 3.3453 ppm. Limiting chemical shifts (H3ax, Ac, H3eq, OMe) are δHG = 1.6154, 2.0388, 2.7205 and 3.3453 ppm; δHG_2 = 1.4550, 1.8456, 2.4800 and 3.0772 ppm; δHG_3 = 1.6067, 2.0162, 2.6980 and 3.3075 ppm. r = 0.999786.

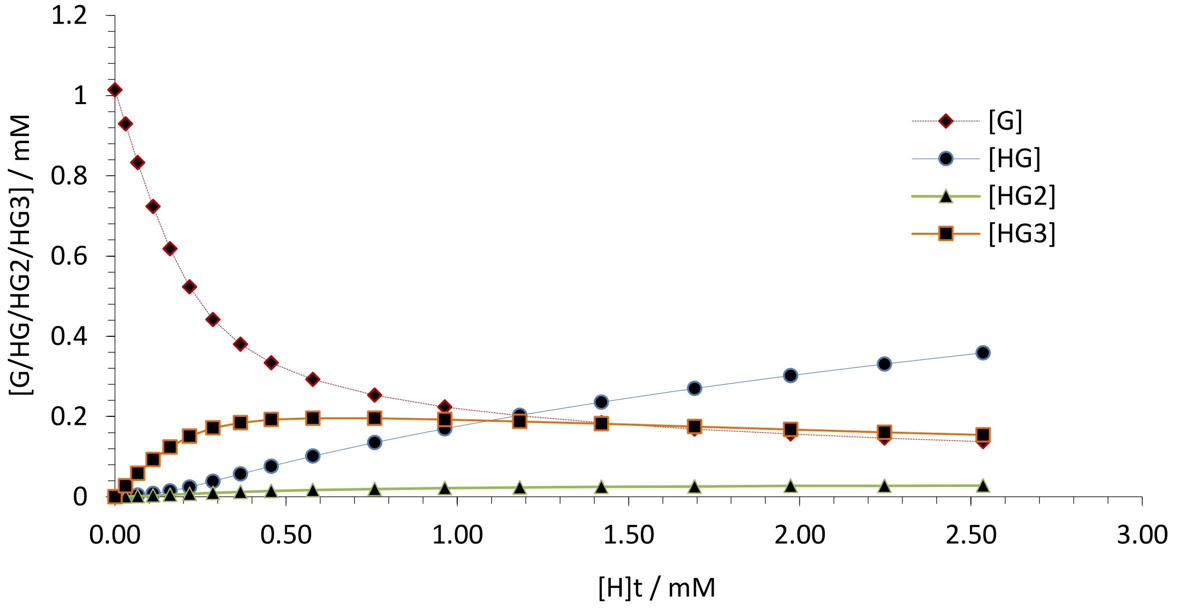

Figure S59. Species distribution resulting from the 1:3 fit of the NMR binding study of methyl α-sialoside **14** titrated with receptor **16** (see above).

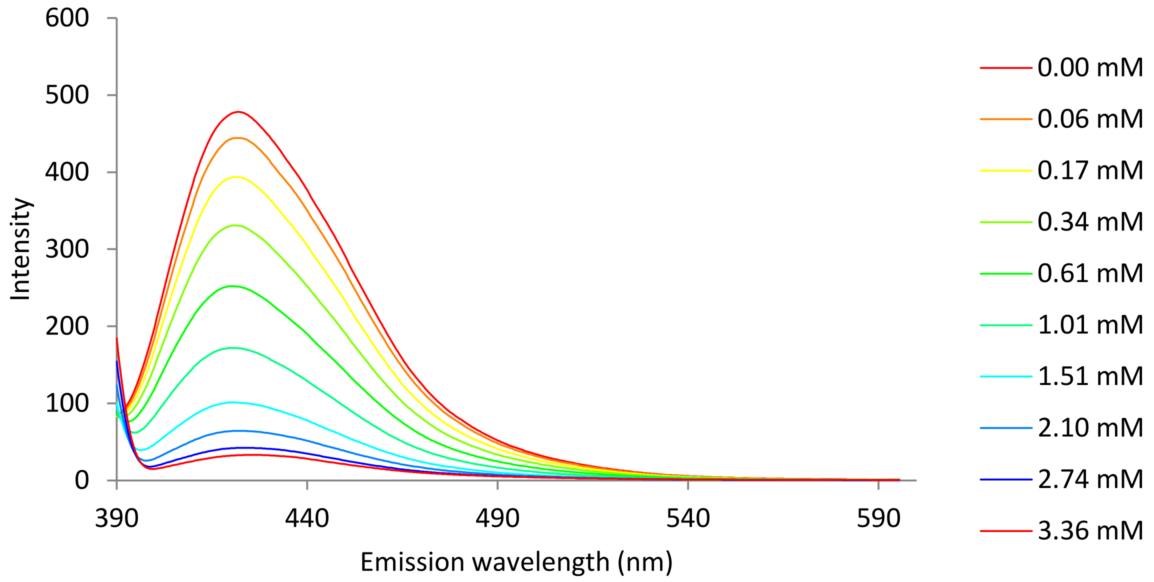

Figure S60. Fluorescence spectra from the binding study of receptor **16** (0.50 µM) titrated with methyl α-sialoside **14** (8.55 mM) at pH 7 in H_2O at 298 K.

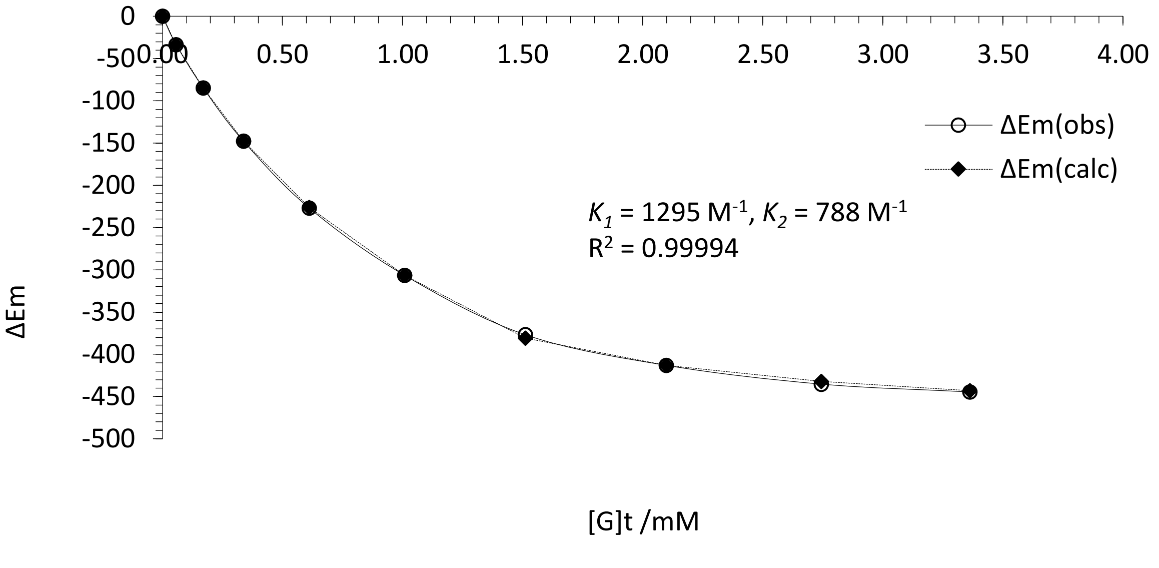

Figure S61. Data analysis for fluorescence binding study of receptor **16** titrated with methyl α-sialoside **14** (see above). Plot of observed and predicted emission intensity (423 nm) against guest concentration (mM), in accordance with a receptor:substrate 1:2 binding model with K_1 = 1295 M^{-1} and K_2 = 788 M^{-1}. Limiting values are Em_{HG} = 10.8 and Em_{HG2} = 0.61. r = 0.99994

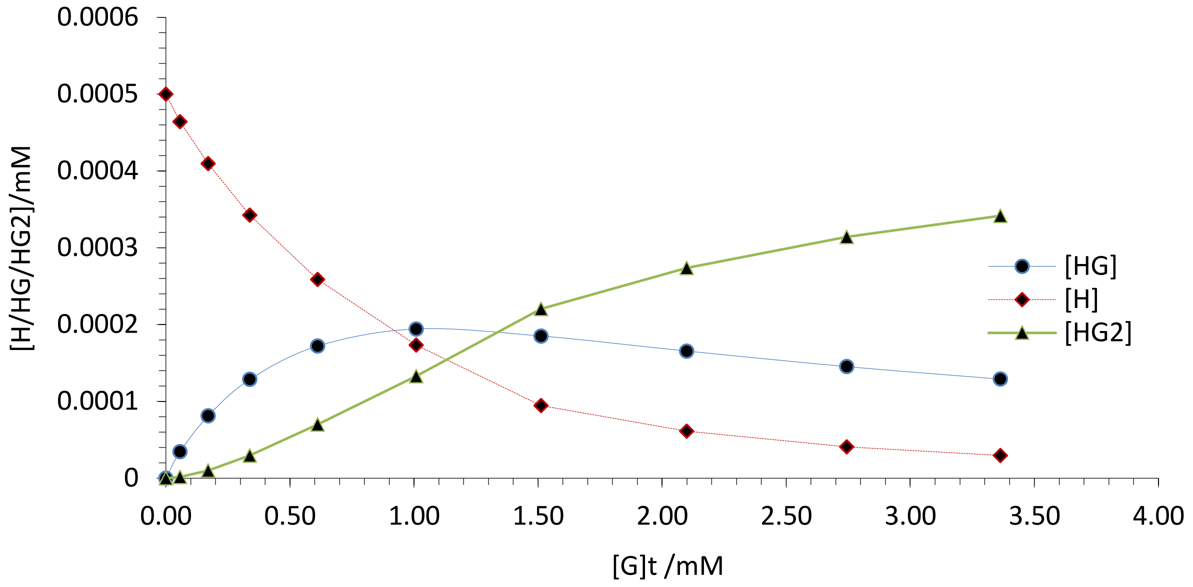

Figure S62. Species distribution resulting from analysis of the fluorescence binding study of receptor **16** titrated with methyl α-sialoside **14** (see above).

Methyl β-D-glucoside

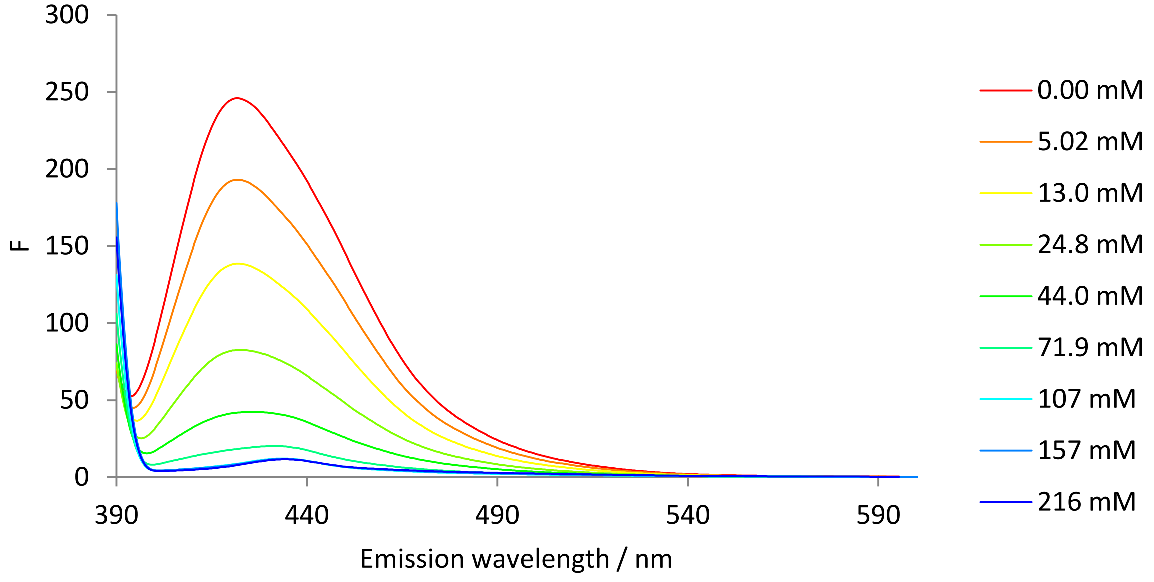

Figure S63. Fluorescence spectra from the binding study of receptor **16** (0.33 μM) titrated with methyl β-D-glucoside (602 mM) at pH 7 in H_2O at 298 K.

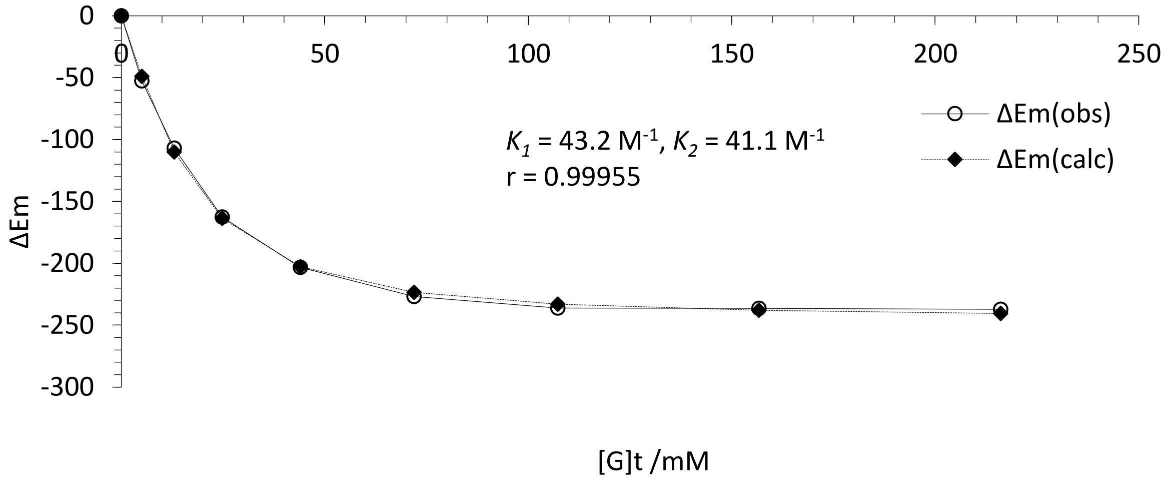

Figure S64. Data analysis for fluorescence binding study of receptor **16** titrated with methyl β-D-glucoside (see above). Plot of observed and predicted emission intensity (423 nm) against guest concentration (mM), in accordance with a receptor:substrate 1:2 binding model, with K_1 = 43.2 M^{-1} and K_2 = 41.1 M^{-1}. Limiting values are Em_{HG} = 10.8 and Em_{HG2} = 1.36. r = 0.99955.

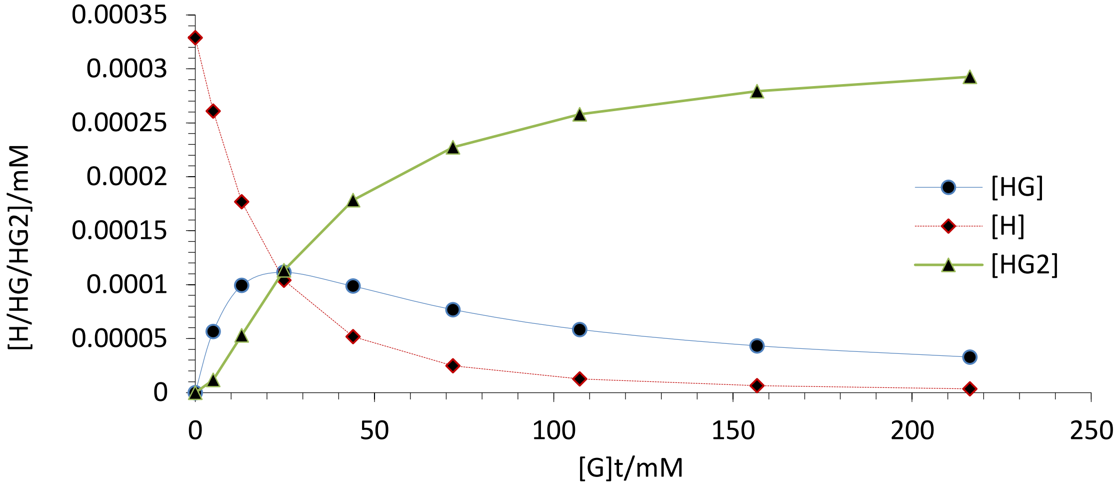

Figure S65. Species distribution resulting from analysis of the fluorescence binding study of receptor **16** titrated with methyl β-D-glucoside (see above).

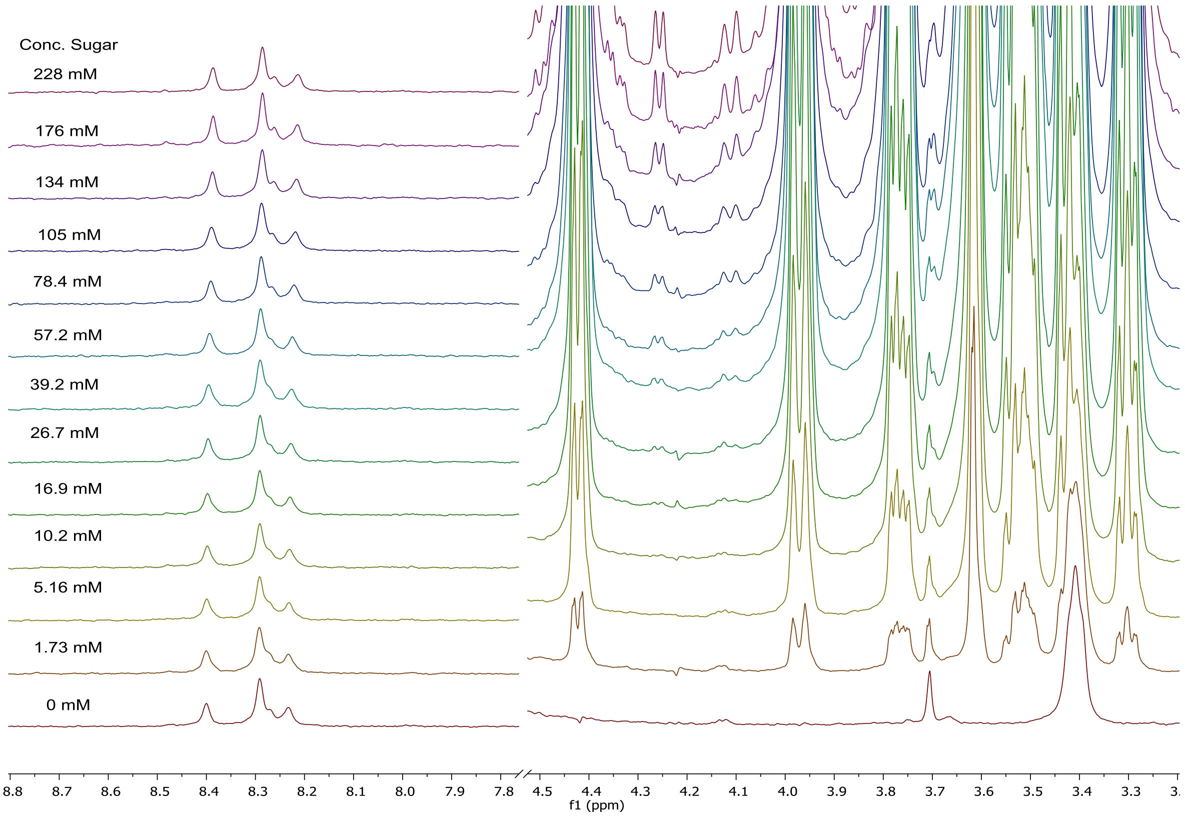

Figure S66. Partial ^{1}H NMR spectra from the binding study of receptor **16** (0.20 mM) titrated with methyl β-D-glucoside (693 mM) at pH 7 in D_2O at 298 K.

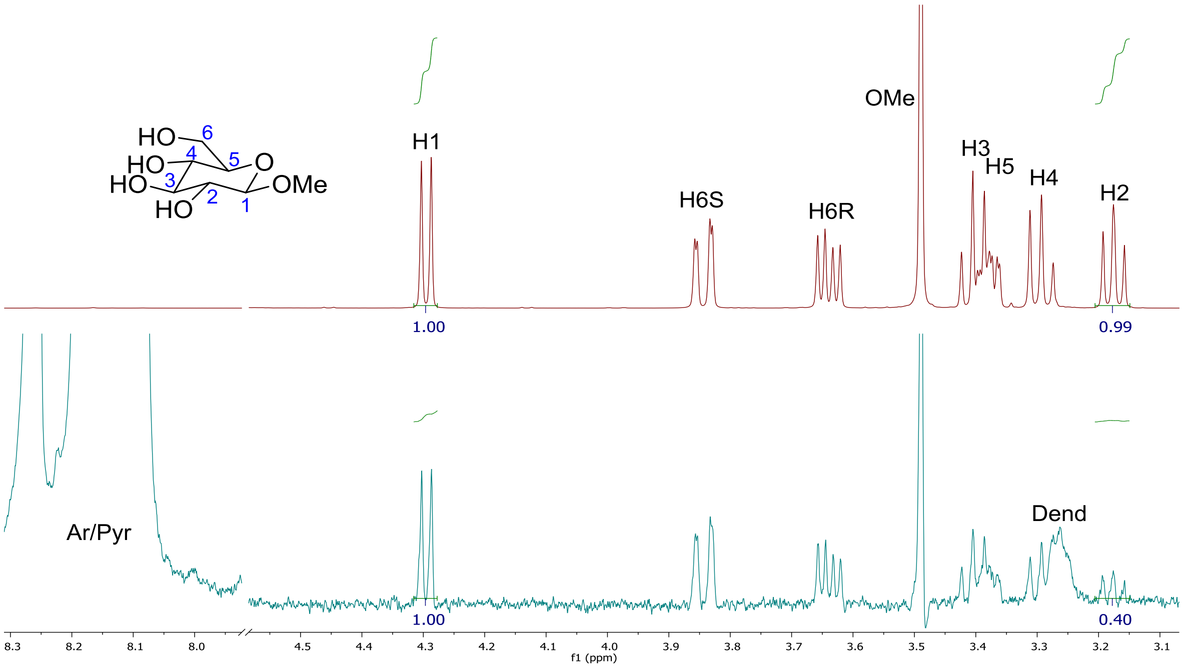

Figure S67. ^1H NMR (top) stacked with ^1H NOESY NMR spectra (bottom) of receptor **16** (0.20 mM) with methyl β-D-glucoside (228 mM) at pH 7 in D_2O at 298 K. Mixing time = 300 ms. Aromatic receptor peaks excited, showing correlation with dendrimers and sugar peaks.

Methyl β-D-galactoside

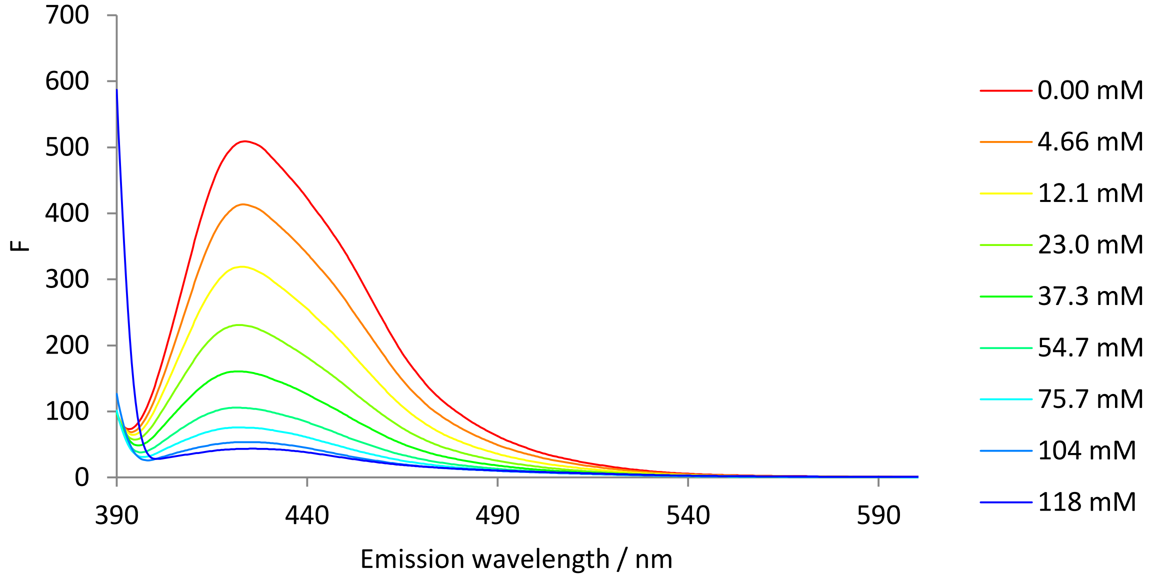

Figure S68. Fluorescence spectra from the binding study of receptor **16** (0.33 µM) titrated with methyl β-D-galactoside (559 mM) at pH 7 in H_2O at 298 K.

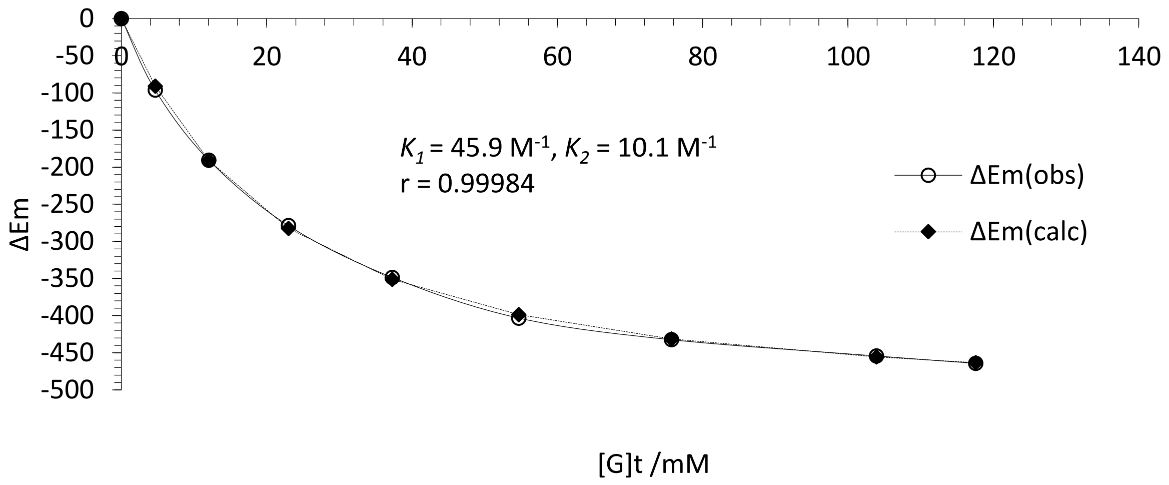

Figure S69. Data analysis for fluorescence binding study of receptor **16** titrated with methyl β-D-galactoside (see above). Plot of observed and predicted emission intensity (423 nm) against guest concentration (mM), fitted using a receptor:substrate 1:2 binding model with K_1 = 45.9 M^{-1} and K_2 = 10.1 M^{-1}. Limiting values are Em_{HG}= 9.96 and Em_{HG2} = 1.31. r = 0.99984.

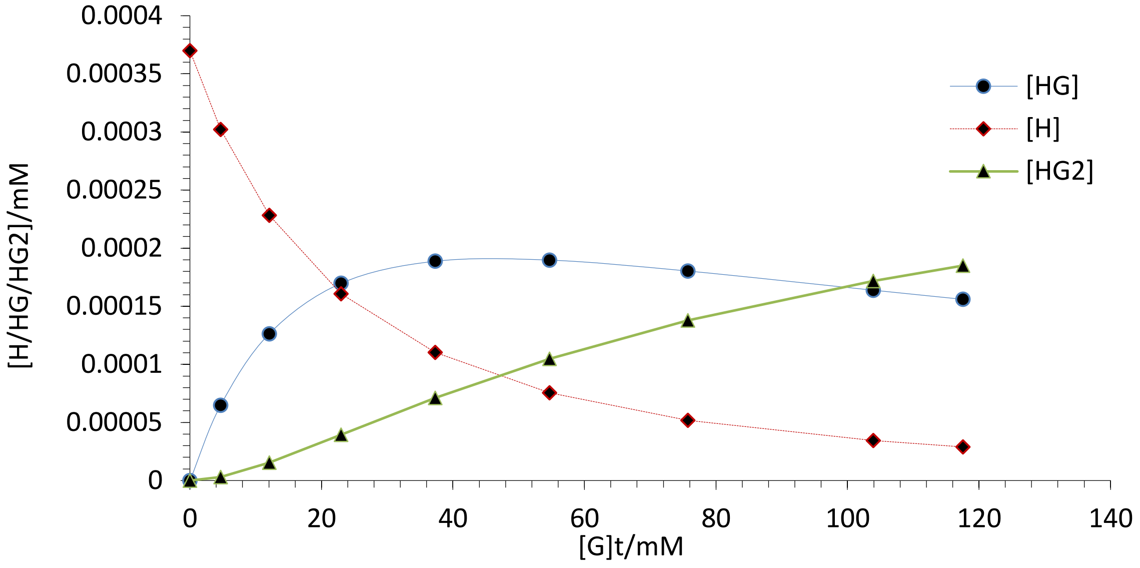

Figure S70. Species distribution resulting from analysis of the fluorescence binding study of receptor **16** titrated with methyl β-D-galactoside (see above).

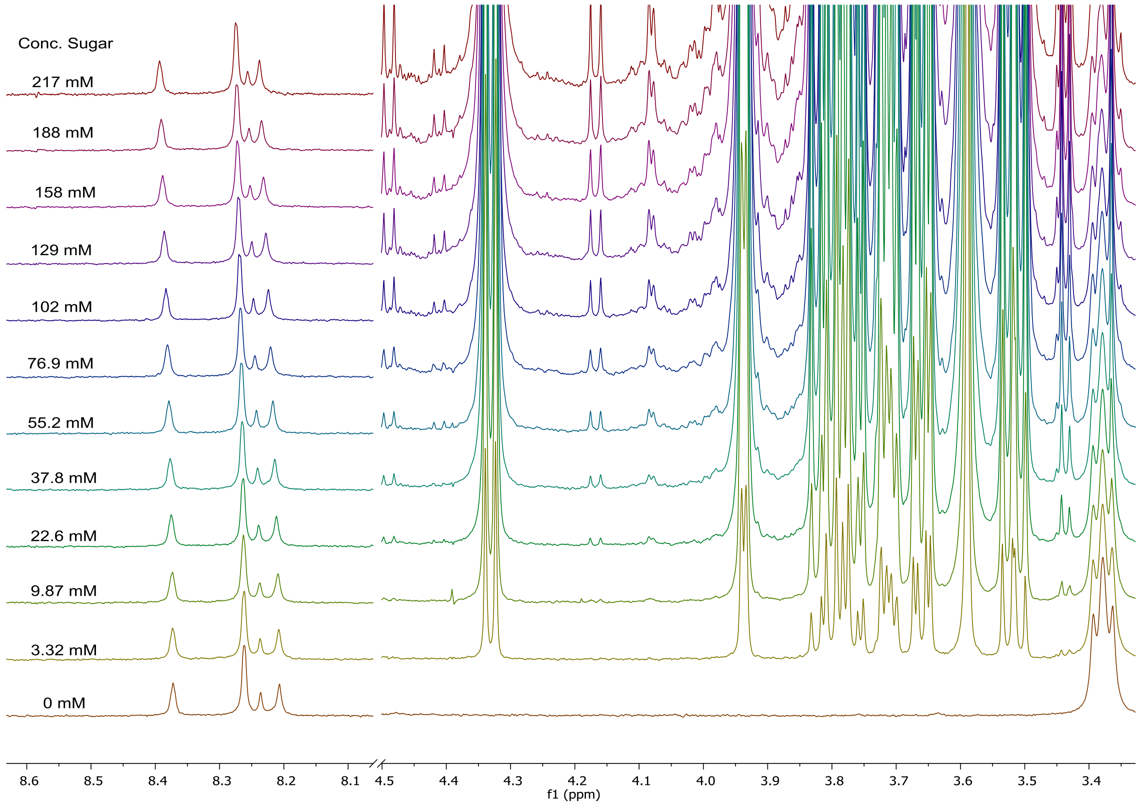

Figure S71. Partial ^{1}H NMR spectra from the binding study of receptor **16** (0.20 mM) titrated with methyl β-D-galactoside (668 mM) at pH 7 in D_2O at 298 K.

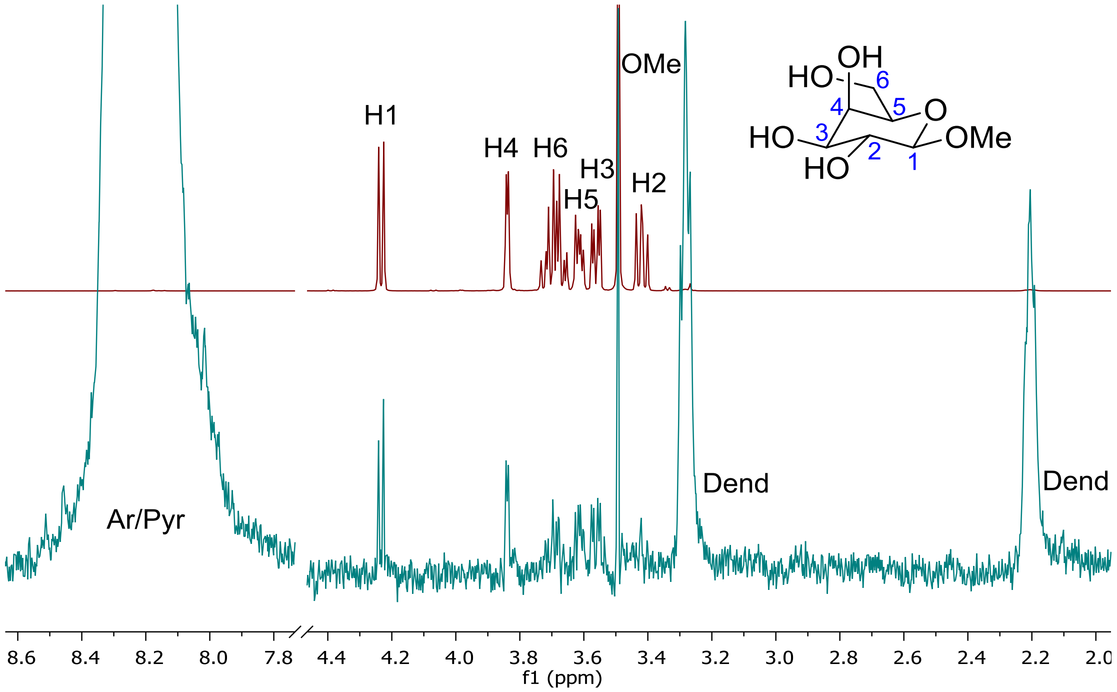

Figure S72. ^{1}H NMR (top) stacked with ^{1}H NOESY NMR spectra (bottom) of receptor **16** (0.20 mM) with methyl β-D-galactoside (217 mM) at pH 7 in D_2O at 298 K. Mixing time = 300 ms. Aromatic receptor peaks excited, showing correlation with dendrimers and sugar peaks. The nOe to H2 is notably weak, suggesting that the pyrene nucleus is closer to the α face of the galactoside.

Glucose

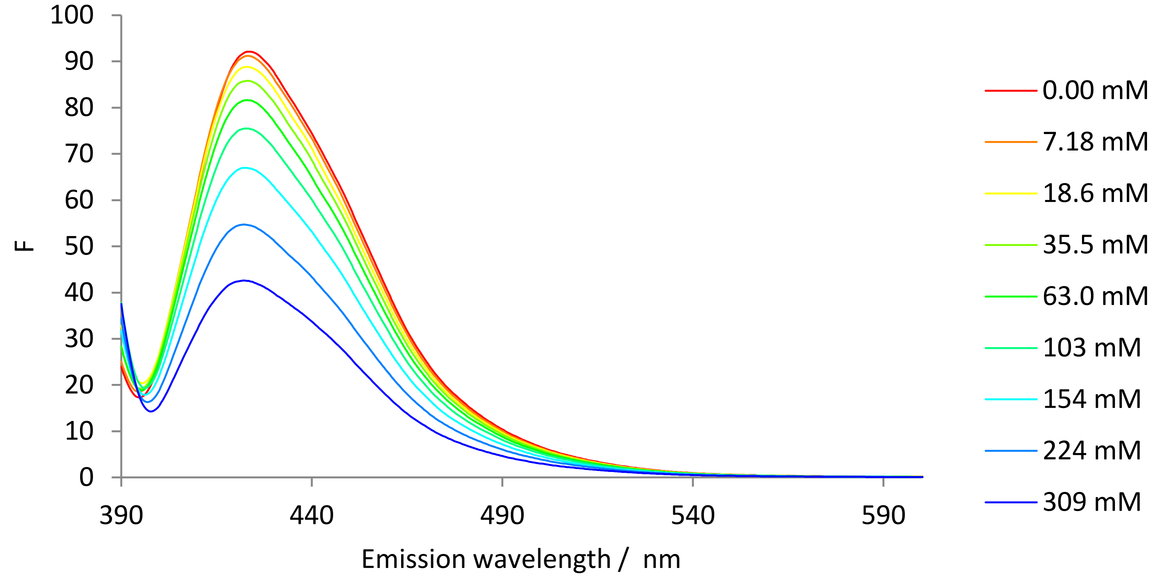

Figure S73. Fluorescence spectra from the binding study of receptor **16** (0.33 µM) titrated with D-glucose (862 mM) at pH 7 in H_2O at 298 K.

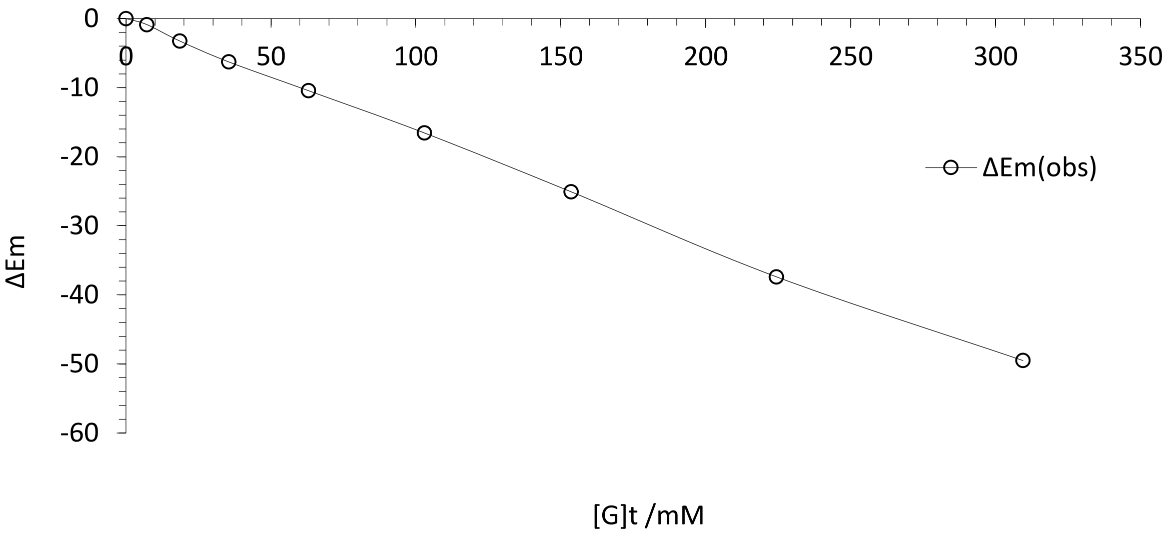

Figure S74. Data analysis for fluorescence binding study of receptor **16** titrated with D-glucose (see above). Plot of observed and predicted emission intensity (423 nm) against guest concentration (mM). Binding too low to be quantified.

Mannose

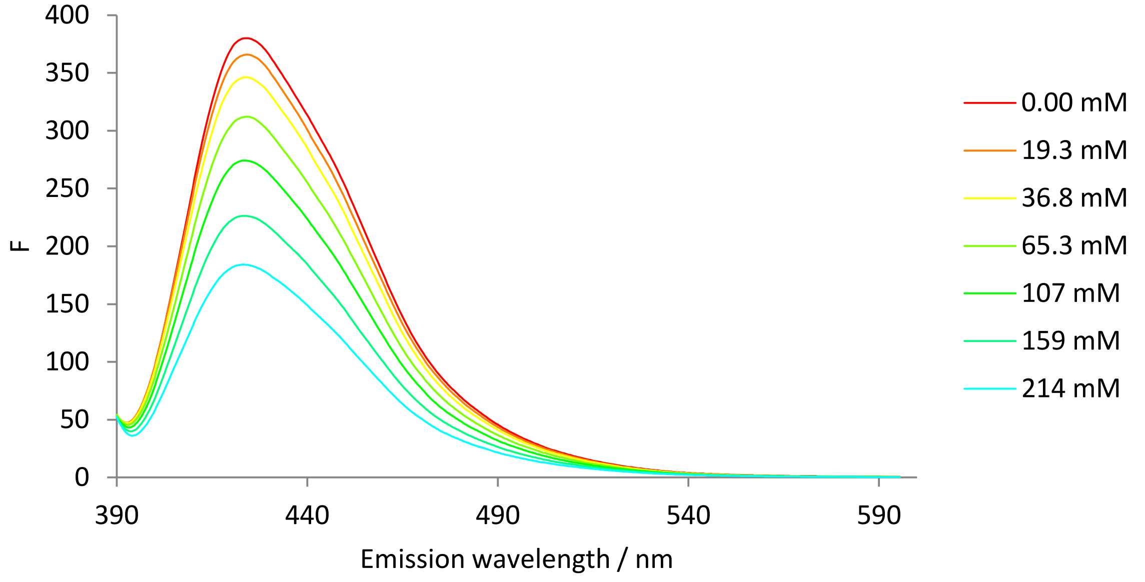

Figure S75. Fluorescence spectra from the binding study of receptor **16** (0.50 µM) titrated with D-mannose (892 mM) at pH 7 in H_2O at 298 K.

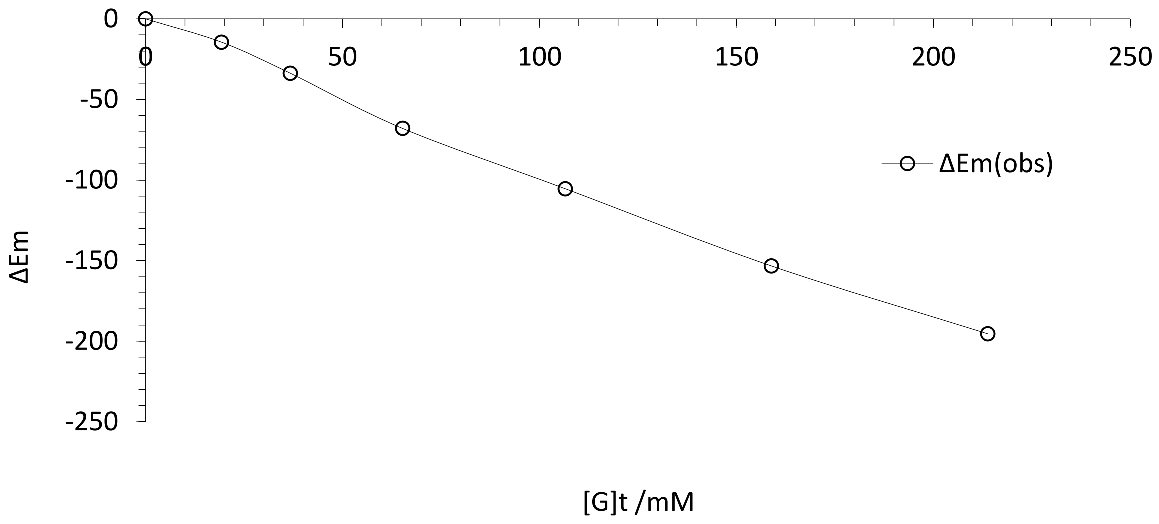

Figure S76. Data analysis for fluorescence binding study of receptor **16** titrated with D-mannose (see above). Plot of observed and predicted emission intensity (423 nm) against guest concentration (mM). Binding too low to be quantified.

Galactose

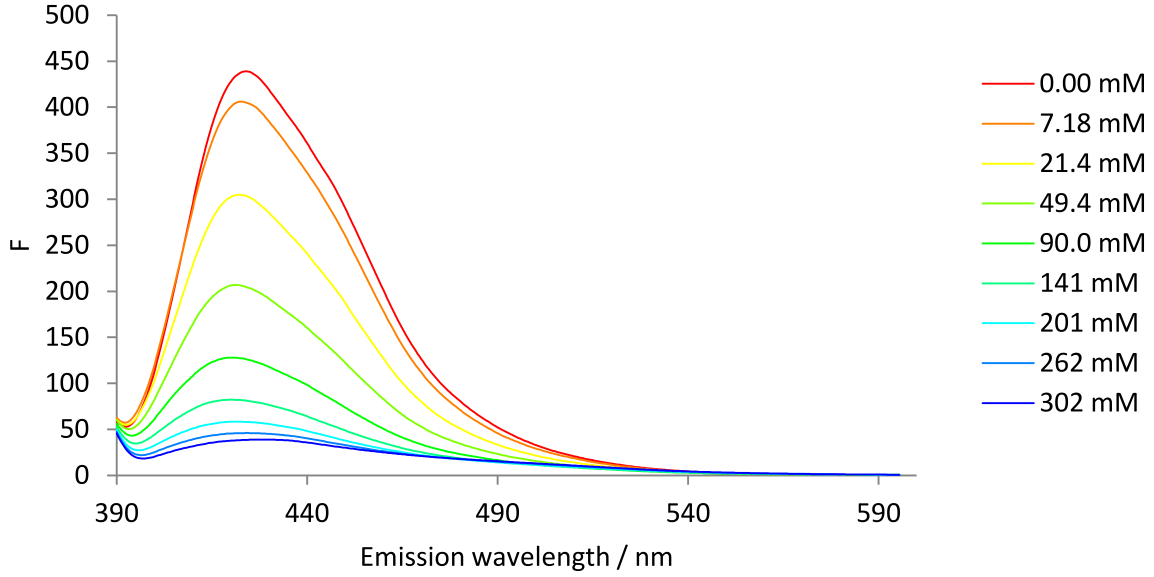

Figure S77. Fluorescence spectra from the binding study of receptor **16** (0.50 µM) titrated with D-galactose (862 mM) at pH 7 in H_2O at 298 K.

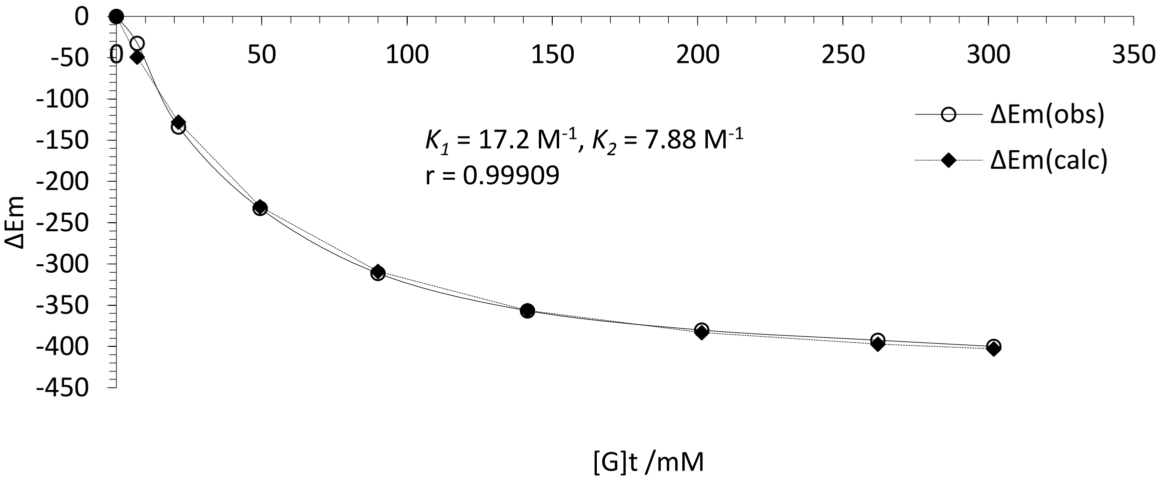

Figure S78. Data analysis for fluorescence binding study of receptor **16** titrated with D-galactose (see above). Plot of observed and predicted emission intensity (423 nm) against guest concentration (mM), in accordance with a receptor:substrate 1:2 binding model with K_1 = 17.2 M^{-1}, K_2 = 7.88 M^{-1}. Limiting values Em_{HG} = 12.8 and Em_{HG2} = 12.3. r = 0.99909.

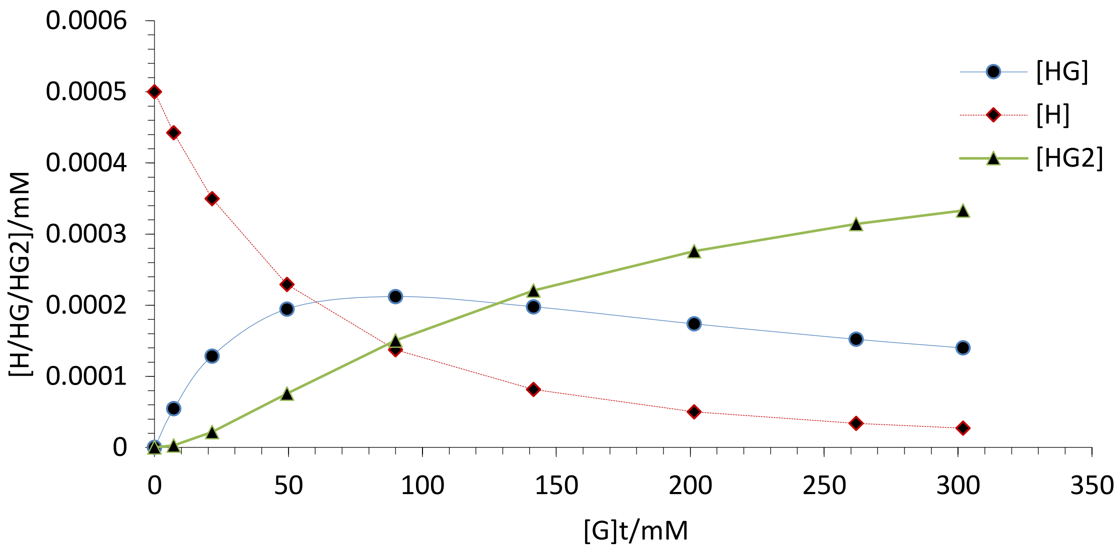

Figure S79. Species distribution resulting from analysis of the fluorescence binding study of receptor **16** titrated with D-galactose (see above).

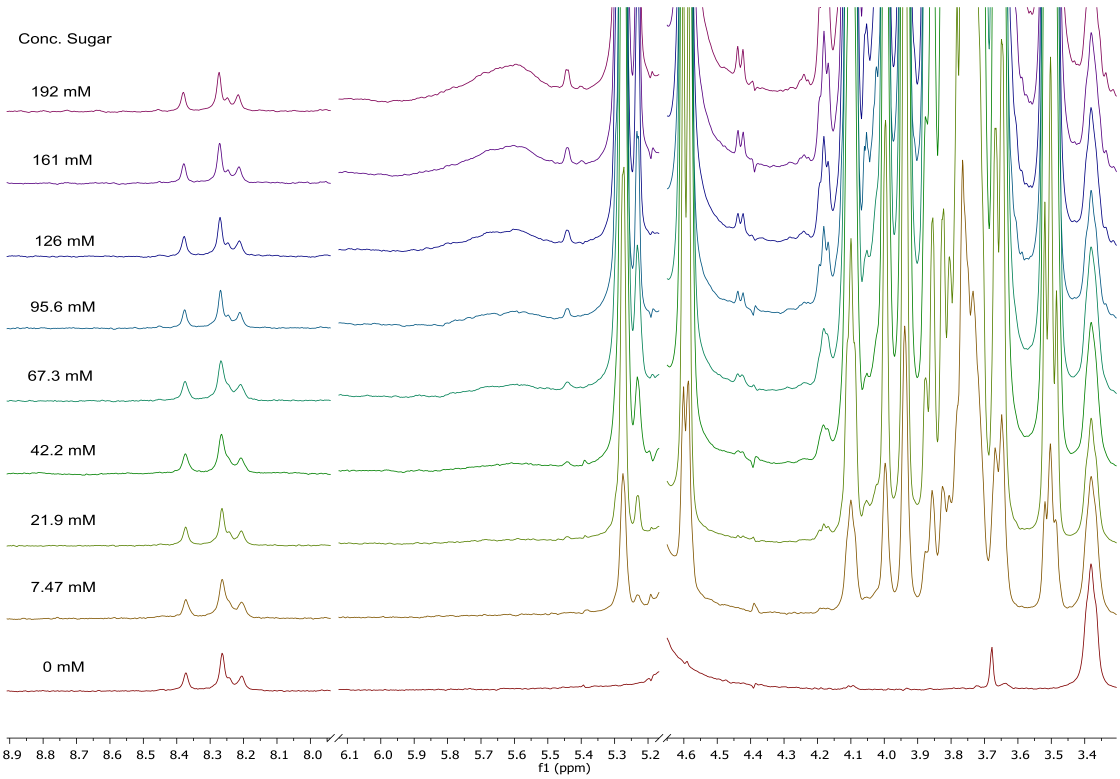

Figure S80. Partial ^{1}H NMR spectra from the binding study of receptor **16** (0.20 mM) titrated with D-galactose (605 mM) at pH 7 in D_2O at 298 K.

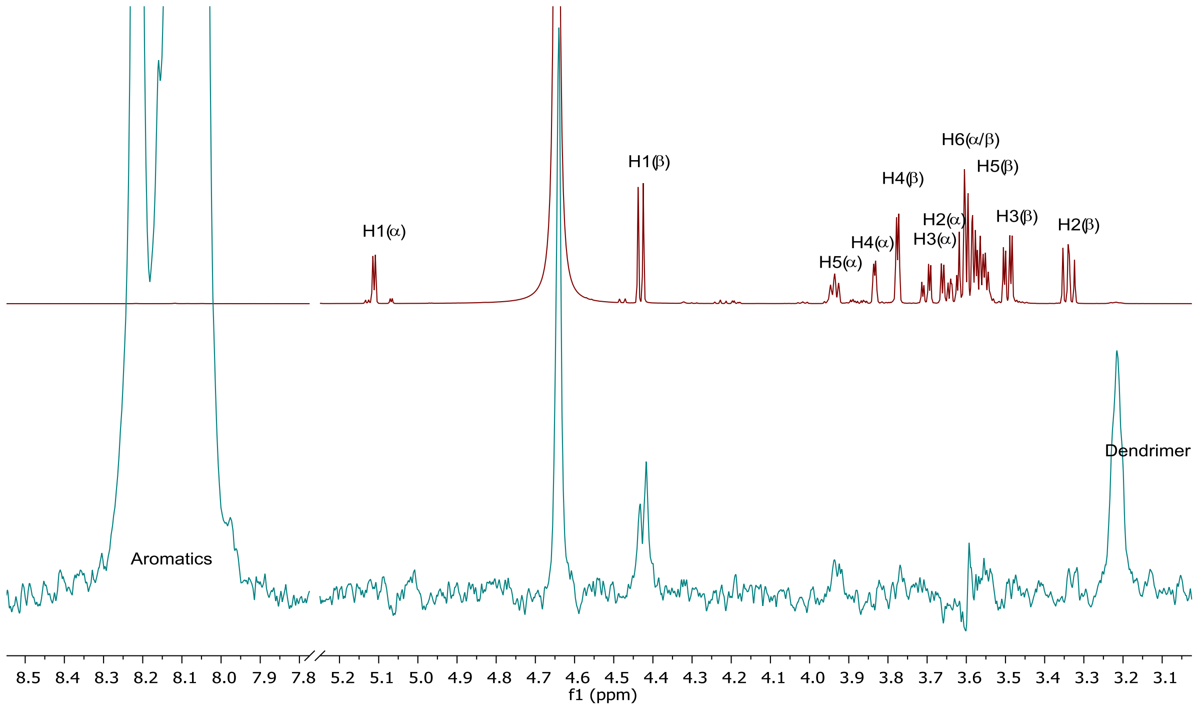

Figure S81. ^1H NMR (top) stacked with ^1H NOESY NMR spectra (bottom) of receptor **16** (0.20 mM) with D-galactose (192 mM) at pH 7 in D_2O at 298 K. Mixing time = 500 ms. Aromatic receptor signals excited, showing correlation with dendrimers and sugar peaks (specifically a strong correlation with H1 of the β-anomer is observed).

Sodium Acetate

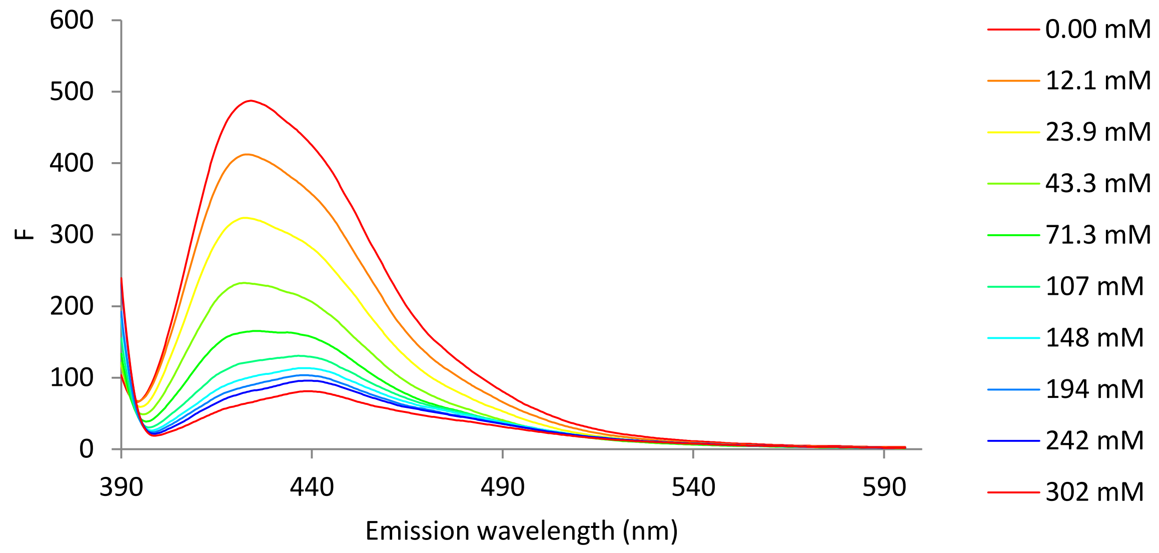

Figure S82. Fluorescence emission titration of receptor **16** (0.50 μM) with sodium acetate (604 mM) at pH 7 in H_2O at 298 K, excitation wavelength 380 nm.

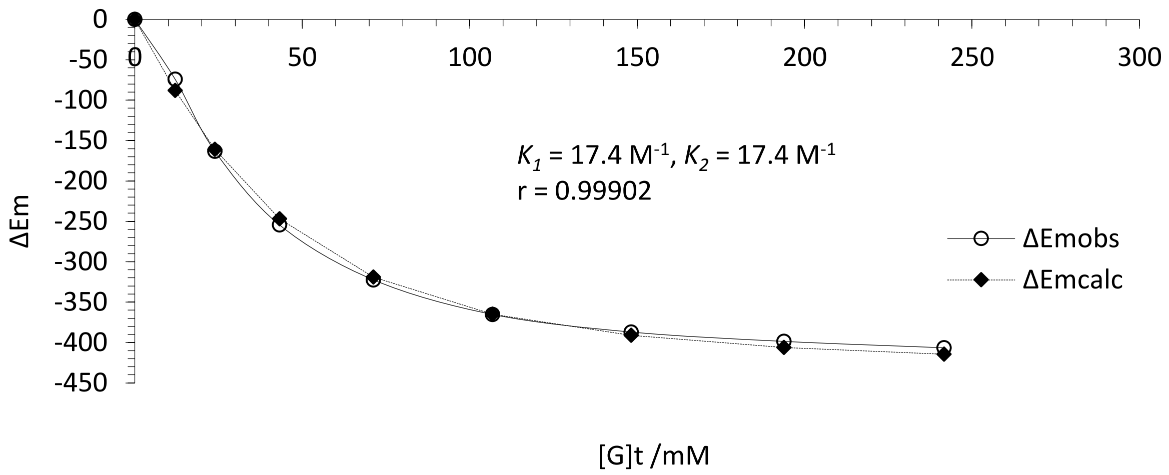

Figure S83. Data analysis for fluorescence binding study of receptor **16** with sodium acetate (see above), in accordance with a receptor:substrate 1:2 binding model with K_1 = 17.4 M^{-1} and K_2 = 17.4 M^{-1}. Limiting fluorescence at 423 nm is Em_{HG} = 53 and Em_{HG2} = 53. r = 0.99902.

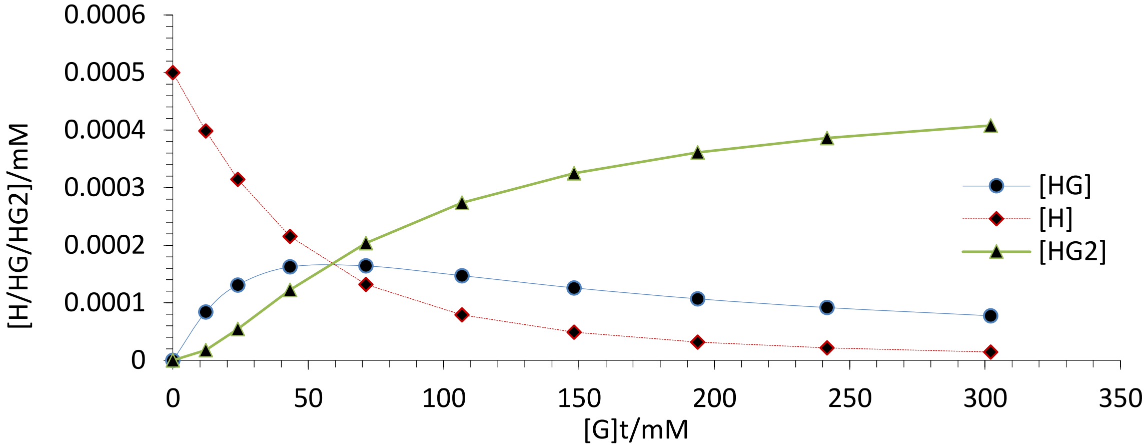

Figure S84. Species distribution resulting from analysis of the fluorescence binding study of receptor **16** titrated with sodium acetate (see above).

Molecular Modelling

Complexes were modelled with Maestro Version 10.4.018. Energy minimisations were performed using Batchmin V11.0, with the MMFFs force-field and aqueous GB/SA solvation. Constraints were used to arrange the complexes in conformations of interest, but were then removed before final minimizations. A model of receptor **9** + protonated mannosamine (**10**.H^+) (β-anomer) is depicted in Figure 2. In this case the conformation was adjusted to bring carboxylate and ammonium groups into proximity before the final minimisation. Figure S85 shows the result for a similar calculation on receptor **16** + methyl α-sialoside **14**. Again it is shown that the receptor can form hydrogen bonds to axial polar group (the carboxylate) while the hydrophobic region of the substrate is positioned close to the pyrene surface.

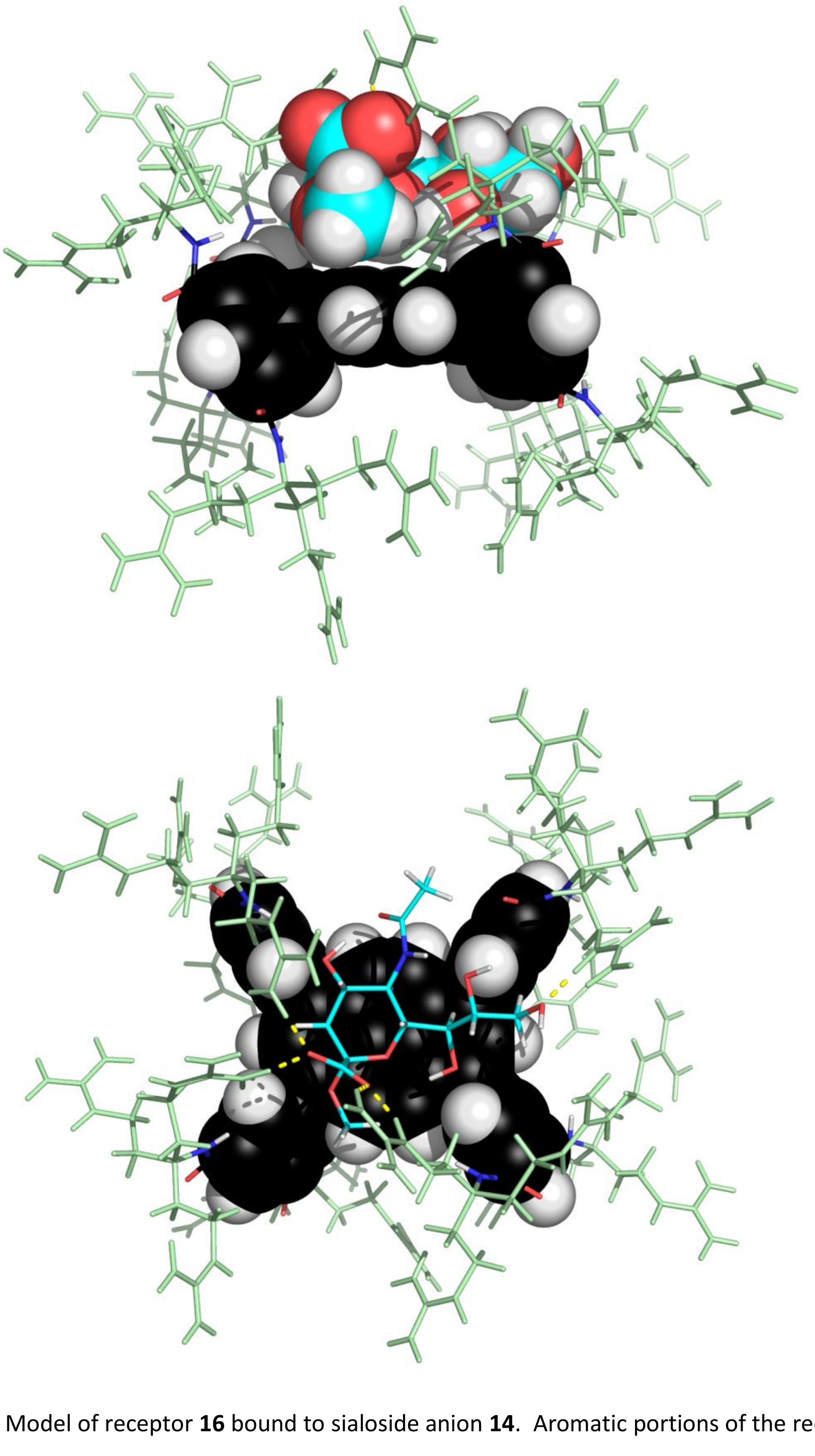

Figure S85. Model of receptor **16** bound to sialoside anion **14**. Aromatic portions of the receptor are shown in space-filling mode, side chains as pale green, sialoside carbons cyan. Top: View from the side, with sialoside in space-filling mode. Bottom: View from above, with sialoside in stick mode. Hydrogen bonds are shown in yellow.

References

1 G. Venkataramana, S. Sankararaman, *Eur. J. Org. Chem.* **2005**, 4162.

2. G. R. Newkome, C. D. Weis, *Org. Prep. Proced. Int.* **1996**, *28*, 495.

3. X. Y. Zhao, K. S. Schanze, *Chem. Commun.* **2010**, *46*, 6075.

4. H. Ogura, K. Furuhata, M. Itoh, Y. Shitori, *Carbohydr. Res.* **1986**, *158*, 37.

5. S. Sabesan, K. Bock, R. U. Lemieux, *Can. J. Chem.* **1984**, *62*, 1034.

6. P. Thordarson, *Chem. Soc. Rev.* **2011**, *40*, 1305.

7. A. Al-Soufi, P. R. Cabrer, A. Jover, R. M. Budal, J. V. Tato, *Steroids* **2003**, *68*, 43.
